# How climate, Indigenous people, and fire shaped Brazil’s Araucaria Forests through the Late Holocene

**DOI:** 10.1038/s41598-026-41607-y

**Published:** 2026-03-28

**Authors:** Oliver J. Wilson, Macarena L. Cárdenas, Claudio Latorre, Hermann Behling, Charlie A. Davies, José Iriarte, Francis E. Mayle

**Affiliations:** 1 School of Archaeology, Geography and Environmental Science, University of Reading, Wager Building, Pepper Lane, Whiteknights, Reading, RG6 6EJ UK; 2 Department of Environment and Geography, University of York, Heslington, YO10 5NG UK; 3 School of Natural Sciences, University of Lincoln, Lincoln, LN6 7TS UK; 4 UK Green Building Council, London, UK; 5 Departamento de Ecología & Centro UC Desierto de Atacama, Pontificia Universidad Católica de Chile and Institute of Ecology & Biodiversity (IEB), Santiago, Chile; 6 Department of Palynology and Climate Dynamics, Georg-August-Universität Göttingen, Göttingen, Germany; 7 Archaeology and History Department, University of Exeter, Exeter, UK

**Keywords:** Ecology, Ecology, Environmental sciences

## Abstract

For millennia, climate changes and Indigenous peoples have influenced Earth’s tropical and subtropical forests. Their relative importance affects our understanding of these ecosystems’ resilience to current anthropogenic changes, so is subject to intensive research and debate. South America’s Atlantic Forest, a global biodiversity hotspot, has been largely absent from this conversation. Here we focus on one of this region’s most iconic, ancient and threatened formations—southern Brazil’s highland mosaic of Araucaria Forest and Campos grasslands. Using novel integrations of palaeo-data and ecological modelling, we assess how climatic and human drivers shaped these landscapes, often through changes to fire dynamics, over the last 6,000 years. We show that climate changes made significant contributions to Araucaria Forest expansions over the last several thousand years, driven by non-linear responses of fire-forest feedback loops to minor climatic shifts. However, within Araucaria Forest areas that experienced more intense human use and occupation, Indigenous people cultivated crops, modified fire dynamics, and profoundly affected vegetation structure and composition. Our results challenge binary views of climate- versus human-driven past vegetation change. Climate, humans and fire all shaped these landscapes through space and time in complex and interacting ways, all of which must be considered to understand or effectively conserve them.

**Supplementary Information:**

The online version contains supplementary material available at 10.1038/s41598-026-41607-y.

## Introduction

Earth’s tropical and subtropical forests make disproportionately large contributions to global biodiversity, carbon sequestration, and climate regulation, and are disproportionately threatened by habitat loss, increasing temperatures and changing rainfall regimes^1,2^. Yet their contemporary ecosystems have been shaped by millennia of natural climatic variations and human impacts. Disentangling their influences can shed crucial light on the forests’ degree of resilience to ongoing anthropogenic disturbances. Compared to some other tropical regions (e.g^3^.), the contributions of pre-colonial Indigenous societies to biogeographic patterns have received little attention in South America’s Atlantic Forest, which covers > 3,000 km of Brazil’s coast and stretches inland to Argentina and Paraguay. Yet the Atlantic Forest, to which one in every 50 seed plant species on Earth is endemic^4^, is the most threatened biodiversity hotspot in the world^5^, endangered by climatic disruption and by deforestation which has already destroyed 63–96% of its natural vegetation^6,7^. The Atlantic Forest’s past has the capacity to shed important light on the factors which generated and maintain its biodiversity, as well as their degree of resilience to human disturbance and climate changes—a critical concern given the region’s precarious present.

Some of the most threatened ecosystems in the Atlantic Forest are found on its southern highlands, in some of the region’s highest and coldest conditions^8^. The area’s subtropical climate with warm summers and high year-round rainfall (1,400-2,200 mm annual rainfall and average annual temperatures of 12–18 °C) is disappearing as precipitation patterns are disrupted and temperatures rise^9^—a looming threat to the plateau’s unique forest-grassland mosaic, which is already endangered after over a century of intense logging and land use change^10^. High-quality fragments of Araucaria Forests—a phylogenetically distinct mixture of different floristic groups—cover as little as 5% of their original area, and over 25% of the old-growth Campos grasslands, a quarter of whose flora is endemic, were lost between 1985 and 2018^10–14^. The mosaic’s vegetation dynamics are being further destabilised by human-driven changes to fire regimes and grazing pressure which, under more natural conditions, maintain grassland areas by suppressing forest expansion^15^. This ancient landscape is characterised by ‘living fossil’ Araucaria trees (*Araucaria angustifolia*, also known as pinheiro or Paraná pine) which are among Earth’s most evolutionarily distinct and globally endangered trees^16^. Devastating 20th -Century logging left the species Critically Endangered, and its historic losses are being exacerbated by 21 st -Century anthropogenic climate changes^17^.

**Fig. 1:**
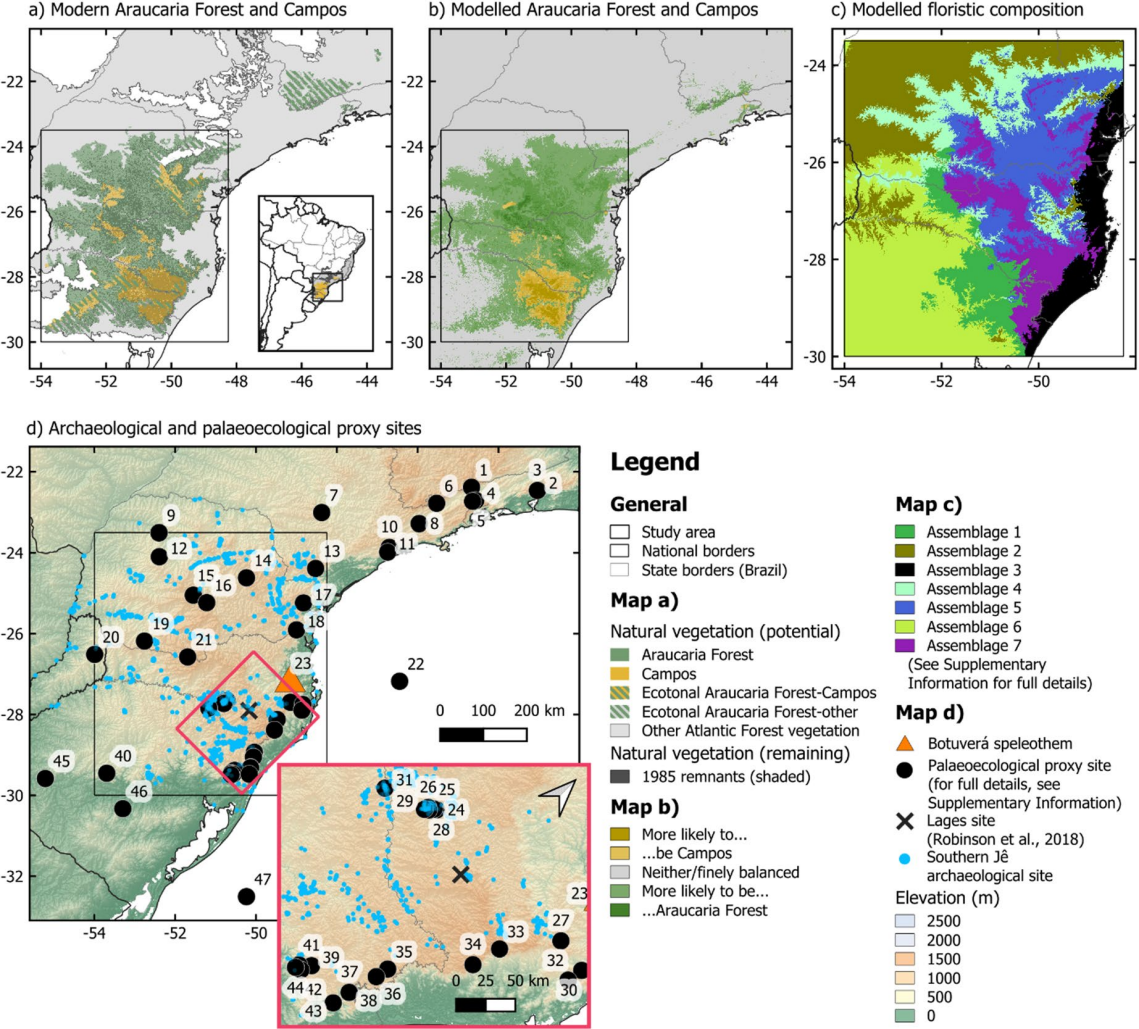
Maps showing (**a**) the natural potential extent of Araucaria Forest and Campos^38^, and their remnant natural vegetation from 1985^39^, (**b**) ecosystem-level ENM for the present-day (see S3.3), (**c**) modelled floristic composition for the study area (see S2.2.3 and S3.3), and (**d**) palaeoecological proxy sites synthesised for this study, with southern Jê archaeological sites. The new palaeoecological records in this study are sites 25 (Abreu e Garcia), 28 (Amaral) and 31 (Pinhal da Serra), in inset. For more information on the synthesised palaeoecological records, see S2.3. Figure prepared by OJW in QGIS 3.14.

Araucaria Forests and Campos grasslands have been part of the southern Atlantic Forest for over 1.5 million years^18^. A dense collection of palaeoecological vegetation proxy records shows how the mosaic developed in time and space, with notable forest expansion occurring around 4,000–3,000 and 1,500–500 years ago (^9^, Fig. 1, S3.1). For over 12,000 years, the landscape has been intertwined with Indigenous people, notably the southern Jê, a group which includes the contemporary Kaingang and Xokleng^19^. The southern Jê archaeological records on the highlands cover the last two millennia, with significant changes—population growth and the development of a highly structured monumental landscape—occurring in the last ca. 1,000 years (Fig. 3c)^20–22^. In pre-colonial times, the southern Jê were at least semi-sedentary, practising a mixed economy in which *A. angustifolia* was critical. In addition to cultural and ritual roles, large, nutritious, abundant, and consistently produced *A. angustifolia* seeds (pinhão) were a crucial and widely traded food resource, which complemented hunting and the cultivation of crops such as maize (*Zea mays*), squash (*Cucurbita* spp.) and beans (*Phaseolus* spp.)^23,24^. Unusually among culturally important tropical trees, *A. angustifolia*’s distinctive pollen, phytoliths and starch grains are clearly identifiable in both the palaeoecological and archaeological records^23,24^.

Through their multi-millennial association, Araucaria Forests played a significant role in southern Jê societies^25^, but to what extent did people influence these forests? Are the southern Atlantic Forest’s highland ecosystems principally cultural relics, previously domesticated landscapes? It is certainly plausible^26,27^, but although the question has been the subject of speculation for decades^26,28^, it has remained unresolved (S1). Three decades of palaeoecological studies closely connected the Late-Holocene expansion of Araucaria Forests with shifts towards more forest-favourable climatic conditions^29–31^, and linked millennia of (likely anthropogenic) enhanced fire activity with forest suppression^32,33^. Increasing evidence that the southern Jê expanded at the same time as Araucaria Forests, however, led to suggestions that humans may have influenced these vegetation changes, eventually contributing to a narrative that they were fundamentally anthropogenic^26,27,34^. Nevertheless, few studies have directly investigated pre-colonial human impacts on Araucaria Forests, and these have not produced definitive evidence (^34–37^, reviewed in S1). The southern Atlantic Forest’s rich palaeoenvironmental and archaeological datasets provide significant potential for resolving the roles of Holocene human land use versus climate changes in shaping contemporary biodiversity patterns in this globally important region^9^. These efforts could shed new and important light on Araucaria Forests’ potential for sustainable use and climate resilience in the 21 st Century, as well as on the roles of fire use and landscape management in the vegetation mosaic more widely.

**Fig. 2:**
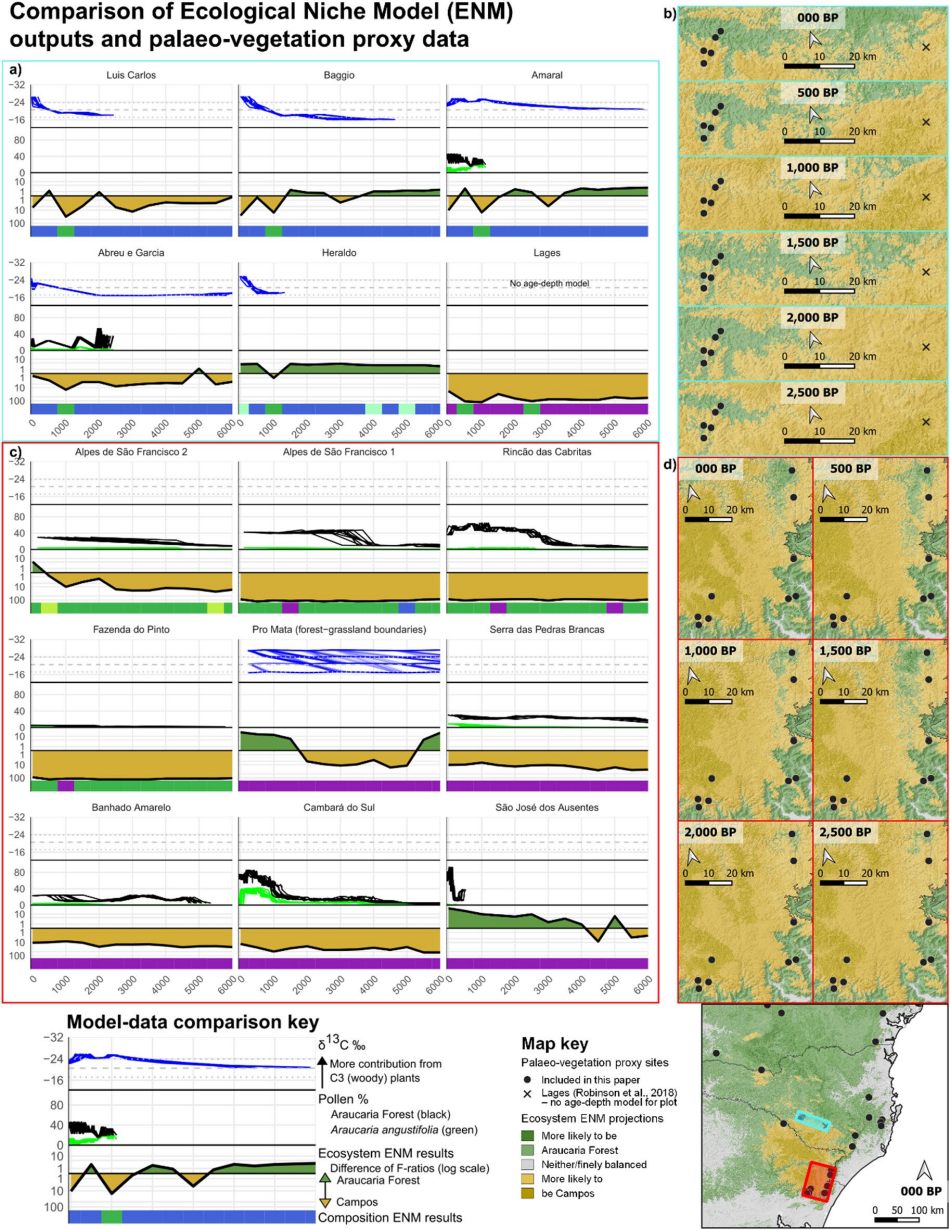
Panels (**a**,**c**) show comparisons between palaeo-vegetation proxy data and ENM projections (see key at bottom). Proxy data are δ^13^C ‰ (blue), and pollen % from Araucaria Forest (black) and Araucaria angustifolia (green), plotted against 10 random iterations of each site’s age-depth model (cal BP, x axis). ENM ecosystem results show changes in relative probability of Campos (in gold, below 0 line) and Araucaria Forest (in green, above zero line), using the difference in their F-ratios (predicted : expected ratio) at each time point, plotted on a log scale. ENM-projected changes in composition are shown in the coloured bar at the bottom of each plot—colours correspond to the ecosystems in Fig. 1c; more detail on the assemblages is available in S2.2. Panels (**b**,**d**) show spatial projections of ecosystem change over the last 2,500 years in the two areas with the most palaeoecological sites. Sites are in the same (reading) order in panels (a) and c) and in (b) and d). Note that Banhado Amarelo and Serra das Pedras Brancas are too close to be distinguished at this scale. Sites in the blue panel (left to right): Luis Carlos (site 29 in Fig. 1), Baggio (site 26), Amaral (site 28), Abreu e Garcia (site 25), Heraldo (site 24), and Lages (cross—proxy data not plotted as the record has only one date); all from this study or^34^. Sites in the red panel: Alpes de São Francisco 2 (site 44)^48^ and 1 (site 41)^44^, Rincão das Cabritas (site 42)^31^, Fazenda do Pinto (site 39)^49^, CPCN Pro Mata (the two lower dots, site 43)^45,50^, Serra das Pedras Brancas (site 38)^51^ and Banhado Amarelo (site 37)^44^(not distinguishable at this scale), Cambará do Sul (site 36)^30^, and São José dos Ausentes (site 35)^46^. Figure prepared by OJW in QGIS 3.14.

**Fig. 3:**
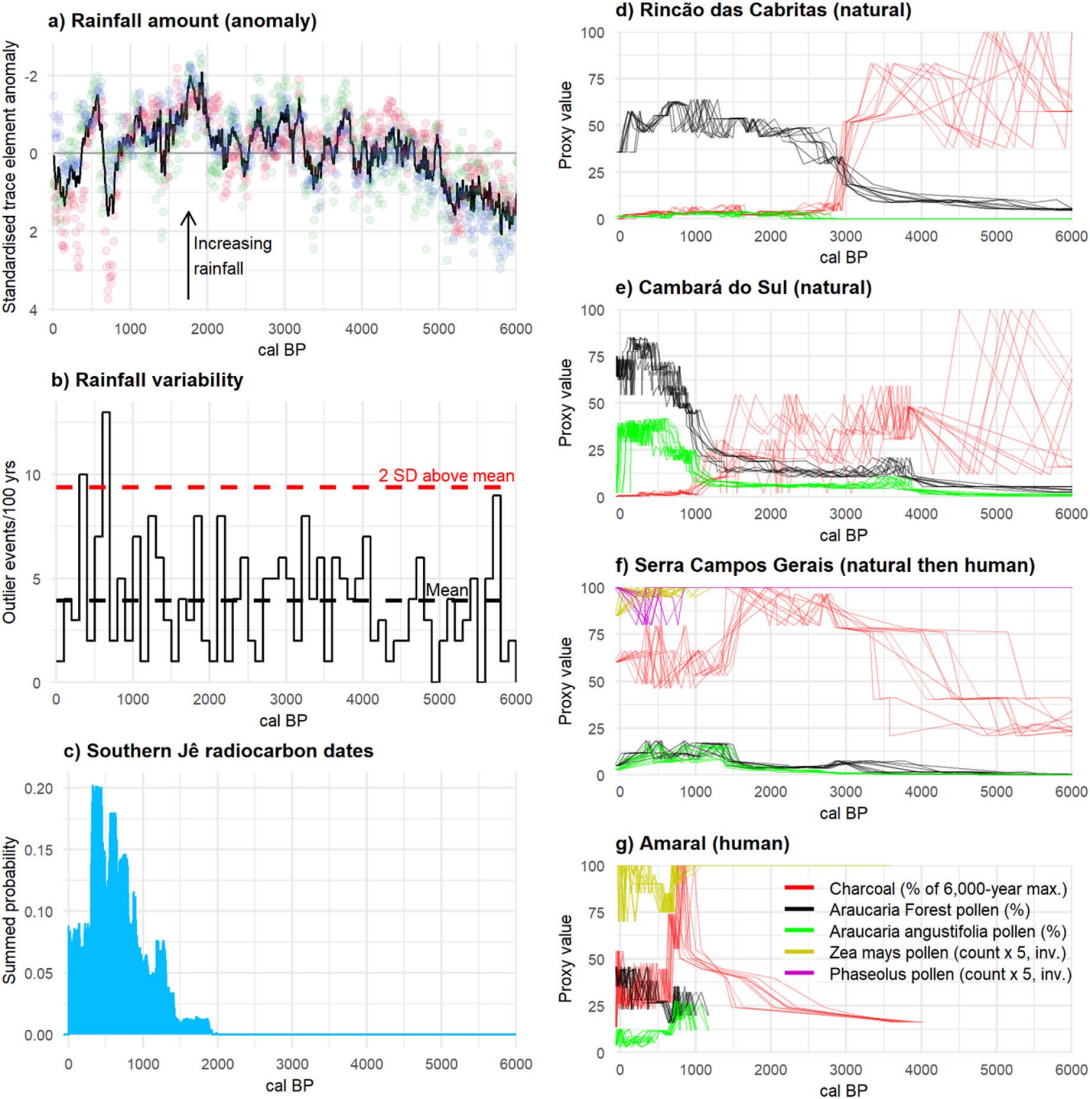
**a**,**b** Proxy record for past precipitation amount (**a**), Mg/Ca, Ba/Ca and Sr/Ca as red, green and blue circles, respectively, and their mean as the black line] and variability (**b**), MAD outlier events per 100-year bin] from Botuverá trace element ratios^40^. (**c**) Summed probability distribution (SPD) of 261 calibrated archaeological radiocarbon dates from southern Jê contexts^57^. (**d**–**g**) Multiproxy palaeoecological records illustrating pre-colonial changes driven by natural climate changes (Rincão das Cabritas^31^, site 42 in Fig. 1d, and Cambará do Sul^30^, site 36), human land use (Amaral, site 28) and a mixture of both (Serra Campos Gerais^52^, site 14). Proxy time series are plotted against ten random iterations of each site’s age-depth model. (Micro-)charcoal concentration is scaled to its 6,000-year maximum in each record. Cultigen pollen (maize, yellow, and beans, purple) is plotted inverted with 5x exaggeration.

**Fig. 4:**
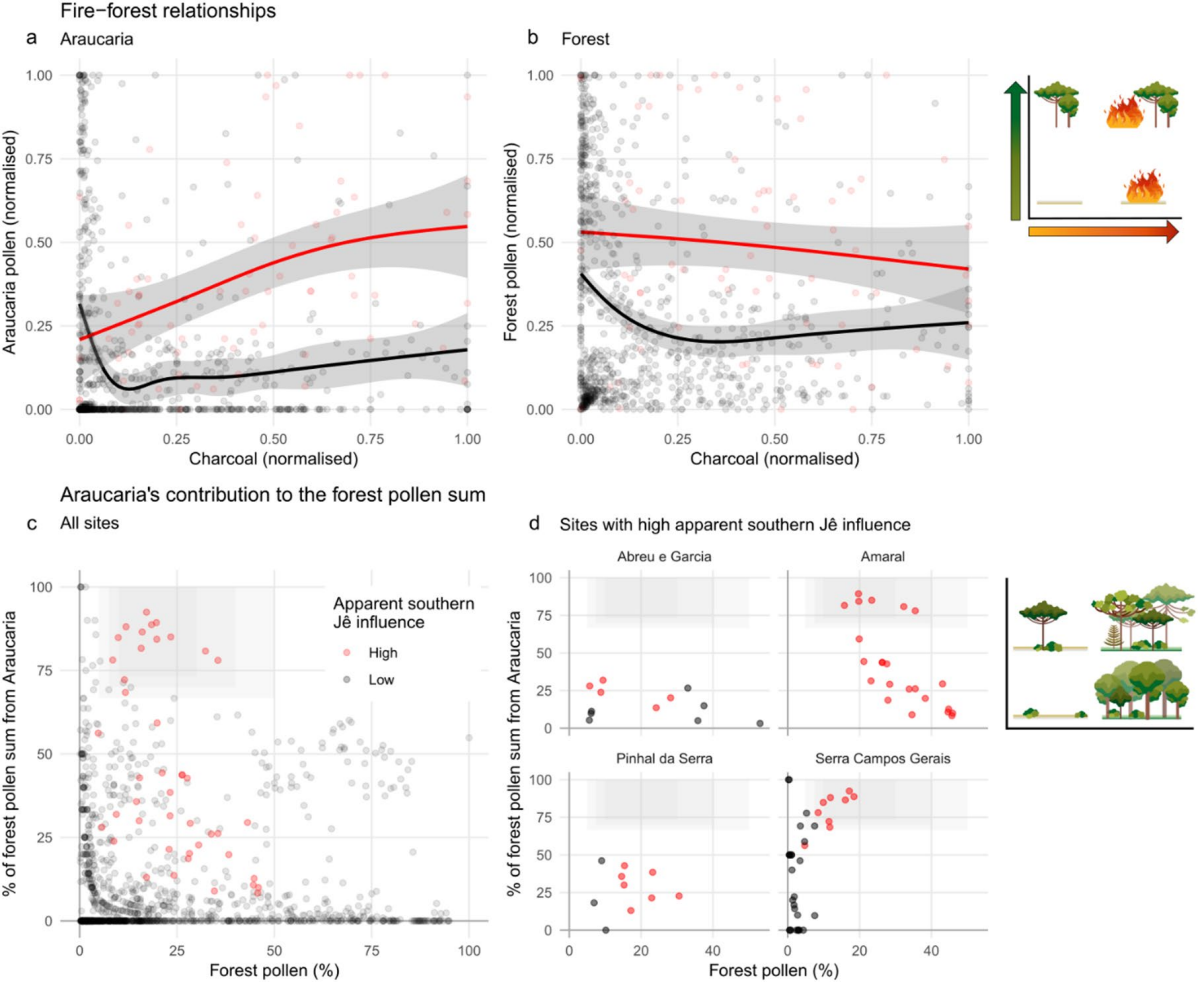
The differences between sites with high (red—last 1,200 years in Abreu e Garcia, Amaral, Pinhal da Serra, and Serra Campos Gerais) and low (black) apparent southern Jê influence. Top: Araucaria (**a**) and (Araucaria) forest (**b**) vegetation against charcoal concentrations. For each site, charcoal concentrations and pollen proportions were normalised before plotting; lines are generalised additive models fitted to the points with shaded 95% confidence intervals. Bottom: contribution of Araucaria pollen to (Araucaria) forest pollen sum for all sites (**c**) and those with high southern Jê influence (**d**). The shaded grey areas in panels (**c**,**d**) highlight subsamples with unusually Araucaria-enriched forest pollen. For per-site comparisons, see S3.1.

In this study we investigate when, where and how climatic changes, fire and human actions shaped Brazil’s Araucaria Forest-Campos mosaic over the last 6,000 years. To do this, we integrate five independent strands of evidence: palaeoclimate records, archaeological data, new and previously published palaeoecological sites, and ecological niche models (ENMs). We use ENMs to generate testable predictions of climate-driven changes to ecosystem distributions and compositions through space and time (Figs. 1b,c and 2; S3.3). We compare these with proxy data from 43 existing palaeoecological sites (Fig. 1d) which reveal observed changes in past Araucaria Forest-Campos dynamics and fire regimes (Figs. 2, 3d-g and 4, S3.1). Potential climatic drivers for these changes are further examined by analysing a high-resolution speleothem record (Fig. 3a-b) and 261 archaeological radiocarbon dates, which show the temporal dynamics of rainfall and human occupation across the landscape (Fig. 3c). Finally, we describe three new multiproxy palaeoecological records, all in close proximity to well-researched southern Jê archaeological sites—the region’s first fossil pollen and charcoal records designed to capture the effects of past human land use on vegetation (Figs. 1c and 3g, S3.2). Together, these different lines of evidence reveal the complex ways in which climatic changes, Indigenous communities and fire combined to shape Brazil’s Araucaria Forests through the Late Holocene.

## Results

Our multistranded approach reveals three main findings: (i) climatic change was a significant driver of Late-Holocene Araucaria Forest expansions over Campos grasslands; (ii) those expansions were transitions between alternative ecosystem stable states, caused by apparently minor climatic changes altering fire-forest feedbacks; and (iii) the southern Jê profoundly shaped the structure and floristic composition of the landscapes in which they lived.

### Natural causes for Araucaria Forest expansions

Various lines of evidence indicate that climatic changes made significant contributions to Late Holocene expansions of Araucaria Forest over Campos. The Botuverá speleothem record^40^ highlights periods of increased rainfall for much of the period between 4,000 and 2,500 cal BP [calibrated years before radiocarbon present, 1950 AD], and even more so from 2,000 to ca. 800 cal BP (Fig. 3a,b). These are the main periods in which palaeoecological records show increases in (Araucaria) Forest and/or *Araucaria angustifolia* pollen, and/or transitions to more C3-dominated (likely woody) vegetation (Figs. 2 and 3d–f, S3.1). Forest expansions in the Common Era tend to be larger and more pronounced than those in the preceding millennia, correlating with the relative magnitudes of the periods’ rainfall increases. With few exceptions, *A. angustifolia* pollen reaches its highest proportions in the last 2,000 years, though it is rarely abundant or an apparent driver of forest expansions; in a number of cases it only becomes a noticeable component of the pollen spectrum in the last two millennia (Fig. 2). (This may be partly due to *A. angustifolia’s* under-representation in some pollen records^41,42^).

These findings alone are insufficient to attribute Common Era expansions of Araucaria Forest and/or *A. angustifolia* to natural causes, since the timings and magnitudes of vegetation changes coincided with changes in both rainfall and the intensity of human occupation of the highlands (Fig. 3a-c). Our ecological niche models (ENMs), however, provide stronger evidence for the role of natural drivers. Driven by climatic variables alone, these ENMs tend to project relative increases in the probability of Araucaria Forest compared to Campos, and/or changes in floristic composition, at the same times and locations that palaeoecological records show vegetation changes (Fig. 2, S3.1, S3.3). These results strongly suggest that the observed changes were indeed largely a consequence of natural climatic fluctuations.

Several sites can be used to illustrate this. Serra das Pedras Brancas (site 38 in Fig. 1)^43^, Banhado Amarelo (site 37)^44^, and CPCN Pro Mata (site 43)^45^ all record increases in Araucaria Forest pollen or C3 vegetation in the Common Era, at locations and times when Araucaria Forest is also projected by our ENMs to increase its likelihood of occurring either in absolute terms or relative to Campos (Fig. 2a, c). Looking beyond the pixel-level locations of palaeoecological sites, we see areas around Cambará do Sul (site 36)^30^ and São Jose dos Ausentes (site 35)^46^ whose conditions are projected to have transitioned from favouring Campos to favouring Araucaria Forest in the last millennium, when both sites record increases in Araucaria Forest pollen (Fig. 2b, d). These sites close to the escarpment edge express these patterns more clearly than do records in the more archaeology-rich Campo Belo do Sul region (Fig. 2a, c; sites 24–26, 28, 29 and 31). However, it is notable that, from 1,000 cal BP onwards, our ENMs project increased suitability for Araucaria Forest at the expense of Campos and/or compositional changes around Campo Belo do Sul, as well as relative stability at more archaeology-free Lages (Fig. 2a-b). Campo Belo do Sul experienced increases in C3 vegetation in the last millennium alongside increased human occupation, whereas vegetation distributions at Lages changed little from ca. 7,500 cal BP onwards, a difference previously attributed to the spatial gradient in past southern Jê occupation (S1)^34^. Instead, our ENMs suggest that natural climatic changes contributed significantly to these differences: in Lages, Campos is projected to have remained much more likely than Araucaria Forest over the last 6,000 years, whereas the two ecosystems were more finely balanced in Campo Belo do Sul (Fig. 2a,b). We also find that the two areas have different projected floristic compositions which, with the results of the ecosystem-level ENMs, may help to explain the different spatial configurations of Araucaria Forest in the landscapes in both regions (Figs. 1c and 2a, S3.3)^34^.

These findings strongly suggest that climate changes were a major driver of Araucaria Forest expansions in the synthesised palaeoecological sites (Fig. 1d). Potential human contributions cannot be entirely ruled out, as these natural climate-driven trajectories of forest expansion could conceivably have been slowed (e.g., to maintain open areas for hunting) or accelerated (e.g., to promote forest resources) by human actions, which could also have resulted in other changes that did not impact Araucaria Forest’s geographic extent (e.g., structural or compositional changes; S4.2). However, our findings demonstrate that it is not necessary to invoke anthropogenic causes to explain any of the previously identified expansions of Araucaria Forest over Campos. Indeed, it is possible that the resources provided by climate-mediated expansions of Araucaria Forest contributed to the intensification of southern Jê occupations, in which new types of domestic and ceremonial architecture emerged around 1,000 years ago^20,34,47^(cf^25^.).

### Natural fire-forest feedbacks drove Araucaria Forest expansions

A number of proxy records show marked increases in Araucaria Forest pollen at certain time periods in the past 4,000 years (Figs. 2c and 3d–f, S3.1). Several of these occur before the first occurrence of southern Jê archaeology around 2,200 years ago (Fig. 3c–e). These relatively abrupt changes are not clearly associated with precipitation changes of similar magnitude in the Botuverá speleothem^40^, though they do occur at times of increased rainfall (Fig. 3a,b). Similarly, while our ENMs predict relative gains for forest at many of these sites and time periods, they nonetheless frequently project that Campos would have remained more likely than Araucaria Forest (Fig. 2c,d, S3.1). However, a clear, consistent and close negative relationship between fire and forest can be observed at almost all sites which record both pollen and charcoal (Figs. 3d–f and 4a,b, S3.1). High concentrations of charred particles are associated with low levels of forest pollen throughout the region, and in previously published cores, pollen from Araucaria Forest (and especially *A. angustifolia*) only increases after declines in charcoal concentrations. Three of the best exemplars of this pattern are Serra Campos Gerais (site 14 in Fig. 1) before about 700 cal BP^52^, Rincão das Cabritas (site 42)^31^, and Cambará do Sul (site 36)^30^ (Fig. 3d–f). In each, sharp declines in charcoal are accompanied by increases in Araucaria Forest in general and *A. angustifolia* in particular.

Fire is an important part of the Araucaria Forest-Campos mosaic^30,53^. Ecological and palaeoecological observations have highlighted that Campos and Araucaria Forest are alternative ecosystem stable states, with their boundaries maintained to a large extent by fires—both natural and, for several millennia, anthropogenic^15,46,54^. Campos species are generally resilient to burning but tree seedlings are fire-sensitive, so fires prevent forest expansions^54^. Fires rarely burn in established Araucaria Forest areas, however, and mature Araucaria trees may be resilient to fire^53,54^. The observed fire and forest dynamics can therefore be explained by a positive feedback loop: relatively minor climate changes (rainfall or temperature increases) slightly favoured tree species and depressed fires, which also helped promote some forest expansion; since forest areas are less susceptible to burning, larger forest areas meant fires became less frequent, which allowed further forest expansion, and so on^55^ (Fig. 6). Such fire-forest feedbacks neatly explain how small changes in rainfall or Araucaria Forest probability could, in places with suitable initial landscape configurations, lead to disproportionately fast and large increases in forest pollen once fires could no longer spread. Adding support to this hypothesis, the abrupt system changes in fire and forest proxies in Rincão das Cabritas, Cambará do Sul and Serra Campos Gerais are all slightly preceded by gentle increases in forest pollen (Fig. 3d–f). These initial responses to more forest-favourable conditions probably initiated the positive feedback loops, and may indicate that critical thresholds of forest cover were passed, causing fire frequencies to plummet^55^.

Climatic changes provide a more likely explanation for these changes than human land use. Conceivably, human actions which increased forest cover could have helped activate or accelerate the fire-forest feedbacks. Conversely, if fires earlier in the Holocene had been both anthropogenic and localised at forest edges, their reduction could also have spurred forest expansions. However, vegetation threshold shifts are more closely correlated with climatic changes than changes in the archaeological record (Fig. 3). Furthermore, pre-colonial human land use in forest ecosystems is most often correlated with increases in fire activity, not its decline (see^32^ and below, but also^56^). Overall, therefore, the simplest explanation for these observed pattens is that they were predominantly driven by shifts to more forest-suitable climate conditions.

### Indigenous communities shaped Araucaria Forests

The great majority of palaeo-proxy records we examined demonstrated a consistent negative relationship between charcoal concentrations and pollen from Araucaria Forest taxa (especially *A. angustifolia*), which fits well with their natural ecological dynamics (Fig. 4a,b, S3.1)^54^. Four sites, however, display systematic departures from these patterns: at Abreu e Garcia (site 25 in Fig. 1), Amaral (site 28), Pinhal da Serra (site 31), and Serra Campos Gerais (after about 700 cal BP; site 14), increases in charcoal co-occur with increases of pollen from *A. angustifolia* and have relatively little effect on Araucaria Forest pollen (Figs. 3f,g and 4a,b, S3.1). Abreu e Garcia, Amaral and Pinhal da Serra are all in very close proximity to known southern Jê archaeological sites (Fig. 1, S2.4.1), and although Serra Campos Gerais is not, all four sites have clear evidence of crop cultivation (pollen from *Zea mays* at all sites and from *Phaseolus* at Serra Campos Gerais) from the same time periods as these modified fire-forest relationships. In these sites, therefore, southern Jê fire use is associated with crop cultivation, increases in *A. angustifolia*, and no significant reductions in forest pollen.

**Fig. 5:**
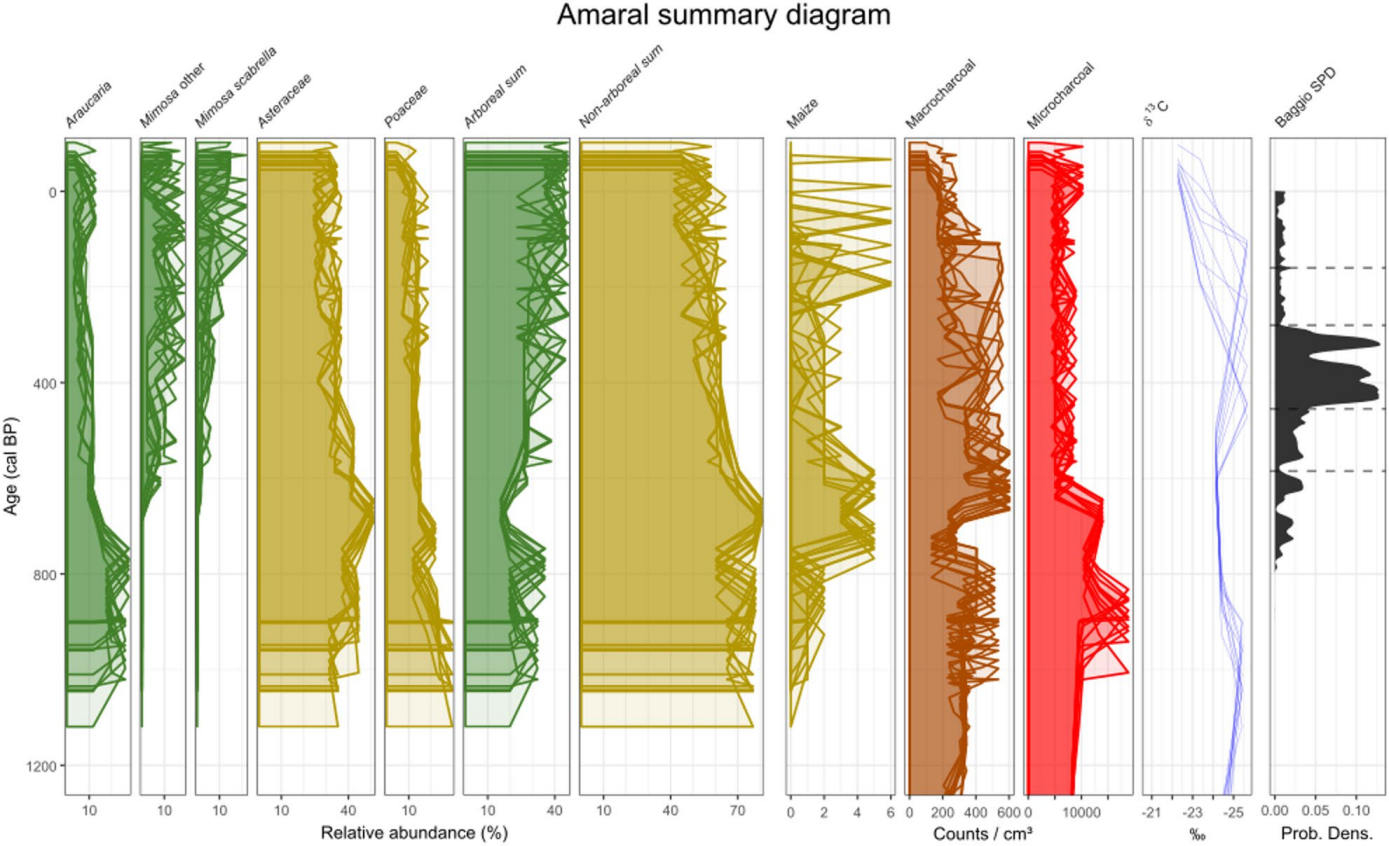
A summary diagram of proxy results from the Amaral record (site 28), with archaeological dates from the nearby Baggio 1 pit house settlement. Proxy values are plotted against ten random iterations of the Amaral age-depth model (S3.2.2), in order to convey chronological (un)certainty with depth. Dashed lines in the Baggio SPD delimit the main phases in the site’s occupation^47^.

In addition to their fire regimes, many sections of the records associated with human-modified landscapes are also distinguished by their forest pollen composition, with *Araucaria angustifolia* pollen generally relatively high and making up a greater proportion of the forest pollen than in other sites and/or periods (Fig. 4c,d). The clearest example of this potentially anthropogenic enrichment with Araucaria trees is found in the early part of the Amaral record (from about 950 to 770 cal BP), where *A. angustifolia* is 13–28% of the total terrestrial pollen (TTP) sum and 78–89% of the Araucaria Forest pollen (AFP) sum; despite declining subsequently, it remains > 40% of the AFP sum for another 350 years (Figs. 3g, 4d and 5). At Serra Campos Gerais, *A. angustifolia* pollen makes up 6–16% of the TTP, and 72–92% of the AFP, sums for almost the entire millennium from 1,300 cal BP (though other tree taxa are quite well represented in non-Araucaria Forest pollen groups; Figs. 3f and 4d^52^). *A. angustifolia* pollen makes up respectively 4–9% and 30–46% of the TTP and AFP sums at Pinhal da Serra between about 1,750 and 350 cal BP (Fig. 4d, S3.1, S3.2). The pattern is not clear in the more variable Abreu e Garcia record. Several other sites show components of this signal but in none of them can these be clearly linked with human land use (S3.1, S4.1); it is feasible that increased *Araucaria* pollen may result in part from natural floristic differences between these generally more westerly sites and the majority closer to the escarpment edge^14^.

The landscape at Amaral between about 950 and 770 cal BP is particularly notable, with pollen assemblages dominated by *A. angustifolia* (13–28%), Asteraceae (38–50%) and Poaceae (12–24%), a C3-dominant δ^13^C signature (−24 to −25‰^45,50^) and high charcoal concentrations (Fig. 5). Nothing similar has previously been observed (Fig. 4c-d, S3.2). The catchment around Amaral may therefore have been a type of parkland, with *A. angustifolia* canopy above Asteraceae shrubland or grassland, influenced by, and possibly kept open with, fire. The presence of other Araucaria Forest taxa cannot be ruled out, since many are poorly represented in the pollen record^41,42^, but most previous studies have more diverse tree pollen than this part of the Amaral record. Its unusual floristic composition and assumed structure, accompanied by maize pollen, high macro- and micro-charcoal, and independent evidence of nearby human activity—charcoal from a fire pit at the Baggio 1 site has been dated to this period^47^—all suggest that this landscape formed as a result of land use by southern Jê groups. This Araucaria parkland could have been used and/or managed for hunting game and harvesting pinhão (edible *A. angustifolia* seeds) as, according to Xokleng-Laklãnõ elders, these activities used to take place in forests that were ‘taller and more open’ before intense contact with colonial society at the beginning of the 20th Century^58^.

The Amaral record also shows a change in the local landscape between 720 and 620 cal BP (Figs. 3g and 5, S3.1, S3.2). Peaks in macro-charcoal and maize, marked declines in micro-charcoal and pollen from *A. angustifolia* and Araucaria Forest, and a temporary change to a more open landscape (as inferred from the δ^13^C record) all coincide with the founding of the Baggio 1 pit house settlement^47^ during a period of significantly variable rainfall (Figs. 3b and 5). This village was continuously occupied from the mid-14th Century to the late 18th Century CE, during which time the region around the site was burned less (lower micro-charcoal concentrations), local fires continued for domestic, ritual and/or small-scale land management purposes (sustained high macro-charcoal concentrations)^47,59^, and maize cultivation was maintained or intensified (increased counts of maize pollen) (Fig. 5). A marked change in forest composition from this time sees *A. angustifolia* begin to decline (though still > 10% of the TTP sum until ca. 350 cal BP), replaced by *Mimosa* and *M. scabrella* pollen. *M. scabrella* in particular is a short-lived early-successional tree species favoured by disturbances, especially fire, so much of its increase may have been incidental^60^. It does, however, produce much-valued firewood and material for construction and woodworking^61,62^, forming near-monodominant forest blocks under modern management^60^, so its proliferation could also have been encouraged and/or managed. Southern Jê use of anthropogenic secondary forest areas around villages for firewood has previously been inferred, and this part of the pollen record could be detecting such areas^63^. It is also possible that this signal may reflect an agroforestry system, similar to some in southern Brazil in which maize, beans and nitrogen-fixing *M. scabrella* trees are intercropped^60^. The steady decrease in the Amaral record’s macro-charcoal concentrations after about 260 cal BP may have been caused by Baggio 1 entering a period of decline (1670–1790 CE, 280 − 160 cal BP)^47^. This decline was part of wider regional changes and losses experienced by the southern Jê, which were most likely caused by the upheavals brought about by European colonisation elsewhere in southern Brazil^32,47,64^.

**Fig. 6:**
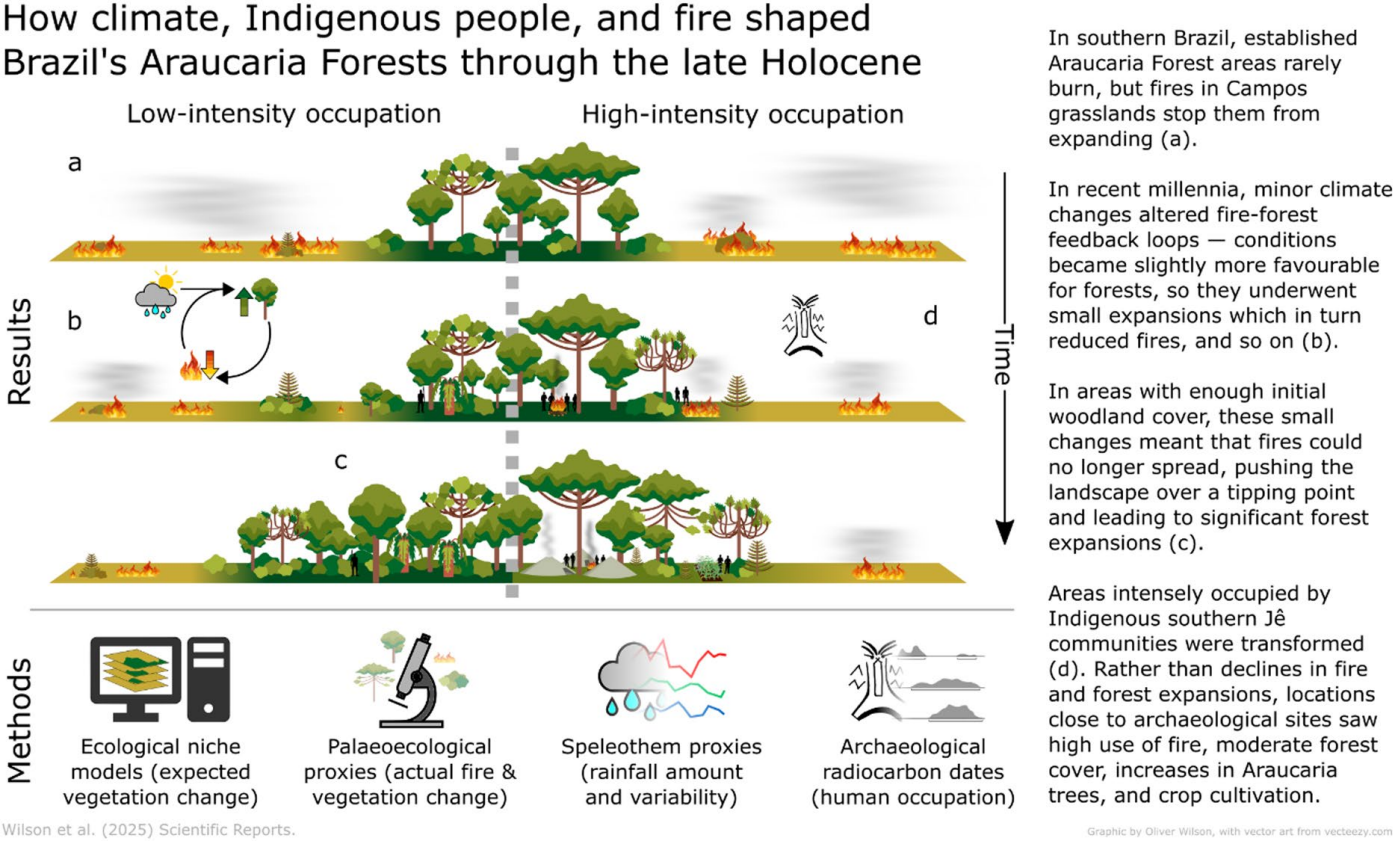
A schematic summary of our methods and main results.

## Discussion

The multidisciplinary approach used in this paper sheds unprecedented light on 6,000 years of natural and human impacts upon southern Brazil’s highland ecosystems. It allows us to compare predicted (modelled) and observed changes in both climate and vegetation, examine regional and local patterns, and probe differences across spatial and temporal gradients from intense to apparently light pre-colonial human impact. We find that climate and humans, each working through alterations to fire regimes, both played significant roles in shaping Araucaria Forest and Campos landscapes in the Late Holocene, and that these factors combined in complex, heterogeneous and interconnected ways (Fig. 6).

Our results show that parts of Brazil’s Araucaria Forests were significantly influenced by the southern Jê. Araucaria Forest generally has a negative relationship with fire, and while young *A. angustifolia* trees establish well in open and disturbed areas, they frequently struggle to reach maturity^65^. However, intense occupation by southern Jê groups—marked by increases in fire and crop cultivation, but not always associated with known archaeological sites—saw Araucaria Forest patches largely sustained and apparently enriched with culturally and economically important *A. angustifolia* trees (Figs. 4 and 5). With local variations, these impacts may have been widespread, as we observe them in four sites across two areas 350 km distant from one another (S4.1). Although there are challenges with applying ethnohistorical and ethnographic evidence to the archaeological record, well-evidenced continuities between southern Jê practices across these timeframes mean such comparisons can be made with care^22,66^. We therefore suggest that the proxy signatures identified above are signals of southern Jê land use and occupation comparable to the ‘casa’ (home) and ‘espaço limpo’ (clean space) domains in contemporary southern Jê ethno-landscapes, where villages and cultivated areas are found and daily activities occur^67,68^, (S4.2). The most extreme manifestations of these domesticated ecosystems can be found at Amaral, where two anthropogenic landscapes, unlike any others in the palaeoecological record and potentially consistent with an agroforestry system, were apparently maintained for a millennium (Fig. 3g).

In areas with less apparent and/or intense Indigenous land use, we find that Araucaria Forest-Campos dynamics—which some authors have attributed largely or entirely to interventions from the southern Jê—predominantly followed expected natural patterns (Fig. 2). These locations saw more pronounced Araucaria Forest expansions in the Common Era than areas with greater archaeological importance, associated with declines (rather than increases) in fire and changes to more forest-friendly climate conditions (Figs. 2 and 3). These patterns were originally considered the result of significant increases in rainfall or temperature, or reductions in the length of the dry season—climatic changes which directly hindered Campos and favoured Araucaria Forest^29,30^. However, the disproportionate speed and magnitude of these forest expansions are better explained by non-linear effects of relatively slight changes in these climatic conditions on fire-forest feedback loops^55^. Still, although natural causes appear to have been the principal drivers of the largest Araucaria Forest expansions, it is important to note that low-intensity Indigenous land use could feasibly have contributed: there are plausible mechanisms for such human activities influencing vegetation dynamics, the process has been observed in similar ecosystems elsewhere in the tropics, and Indigenous ecological knowledge suggests it may have occurred in southern Brazil’s highlands too (S4.2). This leaves open the question of pre-colonial human impacts in areas less intensely occupied or used by Indigenous people—areas perhaps corresponding to the ethno-landscape domain which contemporary southern Jê groups call ‘floresta virgem’ (a space for hunting, ritual activity and contact with spirits)^67^. Such low-intensity use is inherently difficult to detect in archaeological and palaeoecological data, but understanding it would further clarify the full extent to which Indigenous people shaped the Araucaria Forest-Campos mosaic across this large and heterogeneous area.

Our findings provide important historical context for conserving the Araucaria Forest-Campos mosaic in the face of acute present and impending threats. The destruction of the highland landscape through the 20th Century has generally been countered with the establishment of strictly protected areas^69^, though they cover far less of this region than in other parts of the Atlantic Forest^70^. These effectively conserve Araucaria Forest cover^10^, but an alternative paradigm of collaborative conservation management with a holistic socio-ecological focus could enhance the system’s general resilience^69^. Our results suggest that centuries of Indigenous occupation helped sustain Araucaria Forest and actively promoted *A. angustifolia*, so there are compelling historical grounds to view at least some modern Araucaria Forest areas as part of a long-established socio-ecological system. Such examples can be clearly seen in the many Indigenous territories which shelter important Araucaria Forest remnants today^10^. However, intense land use in the past occasionally led to marked changes in forest composition: the occupation history of these areas does not make them immune to negative impacts from radically different human activity in the present.

Effective conservation measures are especially important because natural, non-linear Araucaria Forest expansions over Campos in recent millennia demonstrate the mosaic’s vulnerability to anthropogenic climate change. Apparently minor increases in rainfall and/or temperature did, and will, significantly alter self-reinforcing (positive) feedbacks between fire and forest cover, pushing the landscape over a threshold between alternative stable states. Under Late Holocene climates, such transitions appear to be naturally irreversible^54^. The tipping points for these changes are extremely hard to predict and cannot be precisely identified from palaeoecological data^41,55,71^, but anthropogenic climate changes, increased atmospheric CO_2_ concentrations, and forest-focused fire suppression in protected areas will all bring them closer^15,32,53^. The benefits of fire for Campos are well known; our finding that Araucaria Forest and fire could largely coexist during pre-colonial southern Jê occupations is more novel. More past-, present- and future-focused research may help establish how these findings could be applied to management approaches with positive impacts for both Araucaria Forest and Campos, as well as for the Indigenous people who have helped shape this mosaic over millennia.

Are southern Brazil’s Araucaria Forests domesticated landscapes? Or were their Late Holocene dynamics principally driven by natural climate changes? The multiple strands of complementary evidence we present provide the most complete insight yet into how the southern Atlantic Forest was shaped over the last 6,000 years. Our results caution against sweeping generalisations: we have shown that the interplay between climate, humans, forests and fire is complex and variable through space and time. Apparently minor climate changes can have outsized impacts on the landscape. There is no neat, single correlation between more intense southern Jê land use and greater abundance or expansion of Araucaria Forest. Intense human impacts on the landscape can be found far from known archaeological sites, but floristic differences across spatial gradients of human land use can still have natural causes. And the ways in which the southern Jê interacted with their land were not uniform, differing within, between and beyond archaeological sites. Ultimately, the Late Holocene formation of southern Brazil’s Araucaria Forest-Campos mosaic is a tale of climate and Indigenous people, fire, forests and grasslands. Without considering each element’s contributions, and the complex ways they are woven together, this iconic landscape can be neither fully understood nor successfully conserved. These findings and the approaches we used to uncover them—combinations of generating and synthesising multiproxy palaeoecological data, considering chronological uncertainty, local and regional archaeological overviews, palaeoclimate data analysis, and modelling ecological niches through space and time—can be applied to similar questions in ecosystems throughout the world. Such insights from the past must play an important role in the urgent quest to understand the degrees of resilience and vulnerability of tropical and subtropical landscapes to climate and land use changes in the present and future.

## Methods

### Overview

We used several complementary techniques to investigate the changes in climate, vegetation, fire, and human occupation in southern Brazil’s highlands over the last 6,000 years. The following sections describe our analysis of independent rainfall proxy data from the Botuverá speleothem; the synthesis of southern Jê archaeological dates; ecological niche modelling of Araucaria Forest, Campos, and their species compositions; the synthesis of existing palaeo-vegetation proxy data; and the generation of multi-proxy data from three new palaeoecological records in close proximity to well-known archaeological sites. Additional detail is available in S2.

### Palaeoclimate data

Data on past rainfall comes from the Botuverá speleothem^40^, whose trace element ratios (Sr/Ca, Mg/Ca, Ba/Ca) relate to rainfall quantity^72^. Botuverá data are available from https://www.ncdc.noaa.gov/paleo-search/study/21060. Each record of trace element ratios was standardised so that its mean over the last 6,000 years was 0 and its standard deviation was 1; the mean rainfall anomaly was then taken from the three standardised ratios. To quantitatively assess the variability of southern Brazil’s rainfall since the mid-Holocene, we calculated the Median Absolute Deviation (MAD) of Botuverá’s trace element ratios (aggregated to decadal resolution), following^73^ (see S2.1 for further detail).

### Archaeological data synthesis

We constructed a summed probability distribution (SPD) to summarise the trends in radiocarbon dates associated with southern Jê archaeological sites. Radiocarbon dates are potentially useful but imperfect proxies for past demographic changes^21,74^, so the SPD is used here simply to provide a high-level overview of temporal dynamics in the known southern Jê archaeological record. We used dates from^57^ which were designated as belonging to the Taquara-Itararé, the southern Jê’s material correlate^20^. We excluded dates that had also been excluded by ^57^, those with no laboratory code, and dates with standard errors above 100 years^34^. We constructed the non-normalised SPD in Fig. 3c from the resulting 261 dates using the R package ‘rcarbon’^75^ following^21^, aggregating dates from the same site into 200-year bins. The Baggio SPD in Fig. 5 was produced from the radiocarbon dates for the sites Valmor Baggio 1 and 2 from^57^, without applying binning.

### Ecological niche modelling

#### Overview

We undertook two modelling sub-studies using ecological niche models (ENMs): ecosystem-level distribution changes in Araucaria Forest and Campos in response to high-resolution (100 m) topoclimatic change, and compositional change in the region’s vegetation (based on 40 forest and grassland species) in response to mesoclimate alone. Further details on our ENM methods are found in section S2.2.

#### Locality data

Locality data for both sets of models were drawn from the area 20–33°S and 43–58°W. Ecosystem-level models used a regular 0.1°-spaced grid of points; locations with remnant natural grassland or forest (MapBiomas 1985^39^ in Brazil, Forest Landscape Integrity Index^76^ in Argentina) in areas of potential Araucaria Forest or Campos (Mapeamento de Recursos Naturais do Brasil^38^ or WWF ecoregions^77^) were assigned as presence records for their relevant ecosystem. Absence records for Random Forest (RF) models were either all points not meeting the criteria for presence records (‘all absence’), or only points with other natural vegetation or which fell outside the ecosystem’s potential natural area (‘natural absence’—shown in main text)—see details in S2.2.1 and results from other approaches in S3.3. To model compositional change, we identified 40 key species from Araucaria Forest (20 spp.), Campos (10 spp.), Atlantic Rainforest and Seasonally Deciduous Forest (5 spp. each) (S2.2.1). Locality data for tree species were taken from the Neotropical Tree Communities database^78^ via^79^; Campos species data came from SiBBr/ALA (https://bit.ly/2PqTd5X) with their coordinates cleaned using the ‘Coordinatecleaner’ R package^80^. Following^81^, we thinned these occurrence records so that only one coordinate remained within every 20 km radius, using the R package ‘spThin’^82^. Presence data cover only a subset of each entity’s potential natural extent, principally as a result of habitat loss and logging from the late 19th Century onwards. However, since areas with lost habitat do not have an obvious, systematic association with climatic conditions, this should not unduly bias the ENM outputs. Using both ‘natural absences’ and ‘all absences’ for the RF models further mitigates against any effect from erroneously considering human-transformed areas to be climatically unsuitable.

#### Environmental data

Environmental data are drawn from two sources: CHELSA-TraCE21k downscaled palaeoclimate data (10.16904/envidat.211)^83,84^ (both species- and ecosystem-level ENMs) and the ASTER Global Digital Elevation Model (v3)^85^ (ecosystem-level only). ASTER data were aggregated from 30 m to 100 m resolution, then used to produce a topographic position index (TPI) following^34^, and a measure of exposure to peak insolation following^17,86–88^. Species-level ENMs used CHELSA-TraCE21k data at 30” (ca. 800 m) resolution; for ecosystem-level ENMs these were bilinearly interpolated to 100 m resolution. CHELSA-TraCE21k temperature data (at 500-year intervals from 6,000 BP to the present) were debiased using CHELSA’s modern (1979–2013) climatology^89^. From these data we calculated ecologically relevant bioclimatic variables, using variance inflation factors to select eight: bio2 (annual mean diurnal temperature range), bio4 (temperature seasonality), bio5 (maximum temperature of the warmest month), bio8 (average temperature of the wettest quarter), bio9 (average temperature of the driest quarter), bio12 (annual precipitation), bio14 (precipitation of the driest month), and bio18 (precipitation of the warmest quarter). Further details on these steps are in S2.2.2. Edaphic variables were not considered since the distributions of Araucaria Forest, Campos, and *A. angustifolia* are all better explained by climatic variables alone^54,90^.

#### Modelling

We used three modelling approaches. For ecosystem-level ENMs of Araucaria Forest and Campos, we employed random forest (RF) algorithms, one set with all-absence locality data and one using natural absences only (see above/S2.2.2), as well as the presence-background Maxent algorithm. Only Maxent was used for species-level modelling, since species-level absence data were not available.

RF models used the R package ‘biomod2’^91^, with 25% of the occurrence data set aside for model evaluation and the remainder used for training (75%) and cross-validation (25%). We ran 50 iterations of the RF algorithm, evaluated them using the AUC and TSS metrics, and selected the model run which had the highest overall score across both metrics and evaluation approaches. For the Maxent models we used the R package ‘ENMeval’^92^, tuning model settings with an approach adapted from^81^. We set 10,000 random background points within the training area and split training presence and background data into four spatial blocks (cf.^93^). For each species we evaluated 50 candidate models, based on ten regularisation multiplier settings (at intervals of 0.5 between 0.5 and 5.0) and five feature class combinations (linear; linear and quadratic; hinge; linear, quadratic and hinge; linear, quadratic, hinge and product). We chose the model with the best combination of high AUC and low omission rate (mean of AUC and 1-OR).

For both ecosystem- and species-level models, we evaluated the selected model runs using the Boyce Index (equivalent to the Spearman rank correlation coefficient) from the ‘ecospat’ package^94^, which varies from − 1 to 1 with 0 representing a random prediction. The Boyce Index also converts model habitat-suitability outputs into predicted-to-expected ratios (i.e. F-ratios—see S2.2.3)^95^. To plot Campos and Araucaria Forest projections together, we recoded all areas with an F-ratio below 1 (less than marginally suitable) to 0 (absent), incremented each ecosystem’s F-ratio by one, log_10_ transformed them, then subtracted the value for Campos from that of Araucaria Forest. Negative values, therefore, represent areas more topoclimatically suitable for Campos, positive values are more suitable for Araucaria Forest, and values at or around zero mostly mean absence (though can occasionally indicate that the two ecosystems are finely balanced). To combine species-level ENMs into floristically similar assemblages, we performed k-means clustering (k = 10) on each time slice in SAGA-GIS, then aggregated these using hierarchical clustering in the R package ‘pvclust’ (S2.2.3). One of the four resulting statistically significant floristic groups was further subdivided to show ecologically meaningful change. See S2.2.3 for information on the clusters’ compositions and ecosystem affiliations, and S3.3 for additional ENM results.

#### Palaeo-vegetation proxy synthesis

We synthesised every available palaeo-vegetation proxy record from southern Brazil’s highlands which included data from the last 6,000 years, had continuous sedimentation (i.e. discontinuous soil carbon isotope records were not included), had radiocarbon date depths and proxy diagrams (e.g. pollen percentages) available, and had at least two dates. We made one exception to the two-date cut-off to include the record from Caçapava do Sul^96^, which has one date but covers only 500 years, so is adequately constrained with a surface date. The 43 included palaeo-vegetation proxy records make this synthesis the most complete to date in the region. For records for which raw data were unavailable, we digitised relevant data for as many of the following as were available: *Araucaria* pollen, the sum of forest pollen or the pollen group to which *Araucaria* was assigned (collectively referred to here as ‘Araucaria Forest’), cultigen counts or percentages, δ^13^C values, and fire proxy values. Using information from the records’ original publications, we constructed new age-depth models for all sites using the R package ‘rbacon’^97,98^ and the SHCal20^99^ and Marine20^100^ calibration curves. For more details on these processes, see S2.3.

The impact of chronological uncertainty has received little attention in studies attempting to link past vegetation, climate, and human changes, but its impact can be significant, severely weakening correlations between ecosystem changes and their potential drivers. We incorporate it here by plotting proxy values against ten random iterations of a record’s age-depth models (S2.3, S3.1, Fig. 3d–g). This approach helps to avoid the false impression of chronological precision that can arise when simply plotting proxy values against mean or median dates, providing a more accurate—if less precise—picture of the timings of vegetation changes and their potential causes.

We used a combination of proxy signals to identify four records which had evidence of high southern Jê influence—the new records Amaral, Abreu e Garcia, Pinhal da Serra (see below), and the previously published Serra Campos Gerais site^52^ (see Results). We apply the label of ‘high apparent southern Jê influence’ to samples in these records from 1,200 cal BP or later, based on dates from local^22,47,59,66^ and regional (Fig. 3c) archaeological sites. For further discussion of changes within sites, see S4.1.

#### New palaeoecological data

We present new multiproxy palaeoecological data from three sites: Amaral (site 28 in Fig. 1d), Abreu e Garcia (site 25), and Pinhal da Serra (site 31) (additional details are in S2.4.1). Each is located within 1 km of previously studied southern Jê archaeological sites in the archaeologically rich Canoas-Pelotas basin^59^. The three new records were studied using pollen, macro- and microscopic charcoal, and stable carbon isotopes (δ^13^C) to reconstruct past vegetation and land use dynamics. For chronological control, seven samples from each site were submitted for radiocarbon dating to Beta Analytic, with age-depth models produced using ‘rbacon’ and the SHCal20 calibration curve (details in S2.4)^97–99^.

For pollen analysis, 1 cm^3^ subsamples were processed using standard methods: deflocculation using 10% KOH, acetolysis and HF^101^. Samples were subsequently coarse-sieved to concentrate large pollen grains, such as *Zea mays* and other cultigens, following^102^. Fine and coarse pollen samples were mounted in silicone oil and counted at 400× and 1000× magnification using a Leica DME binocular microscope. Pollen identification used regional pollen atlases^103–107^ and the University of Reading’s Tropical Palaeoecology Research Group modern pollen reference collection, assembled in part with specimens from the Museu Botânico Municipal (Curitiba, Brazil) and CENA (University of São Paulo, Brazil). Aquatic taxa and spores were counted but excluded from the terrestrial pollen sum. Due to poor preservation, especially in the Pinhal da Serra core, it was not possible to count all samples to the standard sum of 300 terrestrial pollen grains (Abreu e Garcia min. 285, mean 331; Amaral min. 247, mean 310; Pinhal da Serra min. 61, mean 111).

Macroscopic charcoal was examined for Abreu e Garcia and Amaral records. Subsamples of 1 cm^3^ were extracted from the sediment cores and soaked in 10% KOH for at least 24 h, then washed through sieves at 100 and 180 μm mesh size. The sieve residues were placed in petri dishes and the charcoal particles were counted at 20x magnification following^108^.

Stable carbon isotope analyses (δ^13^C) were performed on bulk sediment samples at the Laboratory of Geochemistry and Applied Stable Isotopes (LABASI) at the Departamento de Ecología, Catholic University of Chile, Santiago, Chile. Samples were analysed using a Thermo Delta V continuous flow Isotope Ratio Mass Spectrometer coupled to a Flash 2000 elemental analyser via a Conflo IV. Reported analytical sample precision is better than 0.2‰.

## Supplementary Information

Below is the link to the electronic supplementary material.

Supplementary Material 1

Supplementary Material 2

## Data Availability

Synthesised palaeoecological proxy data and all modelling results are available in the supplementary information. New palaeoecological proxy data will be publicly archived upon publication.
