# Supplementary material for: How climate, Indigenous people, and fire shaped Brazil’s Araucaria Forests through the Late Holocene
Source: Sci Rep. 2026 Mar 28;16:10810. doi: 10.1038/s41598-026-41607-y (PMC13039178; doi:10.1038/s41598-026-41607-y)
Supplement: Supplementary file 1 — Supplementary Material 1 [file 41598_2026_41607_MOESM1_ESM.docx]

How climate, Indigenous people and fire shaped Brazil’s Araucaria Forests through the Late Holocene

Supplementary information

Wilson, O.J.*, Cárdenas, M.L., Latorre, C., Behling, H., Davies, C.A., Mayle, F.E.

[*owilson@lincoln.ac.uk](mailto:*owilson@lincoln.ac.uk), [oliver.wilson@york.ac.uk](mailto:oliver.wilson@york.ac.uk). <https://orcid.org/0000-0002-1834-7542>

Contents

[S1. Reviewing the evidence for pre-colonial influence on Brazil’s Araucaria Forests 2](#_Toc213421485)

[S2. Supplementary methods 6](#_Toc213421486)

[S2.1. Palaeoclimate data 6](#_Toc213421487)

[S2.2. Ecological niche modelling 6](#_Toc213421488)

[S2.2.1. Locality data 6](#_Toc213421489)

[S2.2.2. Environmental data 9](#_Toc213421490)

[S2.2.3. Modelling 11](#_Toc213421491)

[S2.3. Palaeo-vegetation proxy synthesis 12](#_Toc213421492)

[S2.4. New palaeoecological data 15](#_Toc213421493)

[S2.4.1. Site overviews 16](#_Toc213421494)

[S2.4.2. Radiocarbon dating 16](#_Toc213421495)

[S3. Supplementary results 17](#_Toc213421496)

[S3.1. Additional palaeo-vegetation synthesis results 17](#_Toc213421497)

[S3.2. Additional results from Abreu e Garcia, Amaral, and Pinhal da Serra 26](#_Toc213421498)

[S3.2.1. Abreu e Garcia 26](#_Toc213421499)

[S3.2.2. Amaral 28](#_Toc213421500)

[S3.2.3. Pinhal da Serra 30](#_Toc213421501)

[S3.3. Additional ENM results 31](#_Toc213421502)

[S4. Supplementary discussion 34](#_Toc213421503)

[S4.1. Identifying evidence of pre-colonial Indigenous impacts on Araucaria Forest 34](#_Toc213421504)

[S4.2. Potential Araucaria Forest transformations from low-intensity pre-colonial land use 36](#_Toc213421505)

[References 37](#_Toc213421506)

# S1. Reviewing the evidence for pre-colonial influence on Brazil’s Araucaria Forests

The description of Araucaria Forests as cultural landscapes, heavily shaped by beneficial human actions in the past, has grown in popularity over recent years. ‘Human-made’ (Bogoni et al., 2020a, p. 2) Araucaria Forest has been characterised as spreading ‘beyond its natural geographic boundaries’ (Robinson et al., 2018, p. 1) in recent millennia, ‘mainly driven by human dispersal’ (Clement et al., 2021, p. 19); ‘pre-Columbian people fostered the expansion’ (Sühs et al., 2021, p. 5) which was ‘induced by past paleoindian populations through human seed dispersal’ (Bogoni et al., 2020b, p. 7), and Araucaria trees only reached their modern limits because ‘humans played an important role in expanding [their] distribution’ (Lauterjung et al., 2018, p. 1). In short, ‘human practices appear to have played an important role in the expansion and formation of Araucaria forests’ (dos Reis et al., 2014, p. 5).

There are indeed good reasons to think that Araucaria Forests could have been domesticated by Indigenous people, but as yet there has been little firm evidence to definitively show that they were. The same small set of studies ultimately underpins most claims, and for various reasons they are unable to fully support the contention of Indigenous domestication of Araucaria Forest, Araucaria trees, or the wider landscape. In this section, we examine 18 studies which either aim to provide, or have been cited as providing, evidence for the idea that contemporary Araucaria Forests are largely the product of pre-colonial Indigenous land use (Bauermann et al., 2008; Behling et al., 2004; Behling and Pillar, 2007; Bitencourt and Krauspenhar, 2006; Bogoni et al., 2018; de Oliveira Portes et al., 2018; dos Reis et al., 2018, 2014; dos Santos et al., 2009; Kern, 1998; Lauterjung et al., 2018; Mello and Peroni, 2015; Noelli, 2000; Pereira Cruz et al., 2020; Robinson et al., 2018; Scheel-Ybert and Boyadjian, 2020; Vasconcellos et al., 2024; Zeder, 2016).

Three of these studies (each cited once) do not in fact deal with the question of anthropogenic Araucaria Forests (Kern, 1998; Scheel-Ybert and Boyadjian, 2020; Zeder, 2016). Three palaeoecological studies (Bauermann et al., 2008; Behling et al., 2004; Behling and Pillar, 2007) have also been cited once each as evidence of human-driven fire coinciding with *Araucaria angustifolia* expansions, though their results do not support this interpretation: Behling and Pillar (2007) is a review drawing heavily on Behling et al. (2004), which records forest expansions occurring only when fire declines, and the record in Bauermann et al. (2008) has very little forest pollen and even less from *A. angustifolia*. A more recent paper from de Oliveira Portes et al. (2018) does not yet appear to have been cited in discussions of deep cultural traces in Araucaria Forests, although it provides clear evidence that Indigenous people used fire to maintain the Araucaria Forest-Campos mosaic in south-eastern Brazil’s Serra da Bocaina. However, it shows neither anthropogenic forest expansion nor notable impacts of humans on forest composition, and *Araucaria* pollen is rare throughout. Several studies which have been used have a focus on human land use and/or domestication processes in modern Araucaria Forest landscapes, finding that some tree populations and forest areas have been significantly shaped by human-environment relationships over recent centuries (Bogoni et al., 2018; dos Reis et al., 2018; dos Santos et al., 2009; Mello and Peroni, 2015). However, all of them look at the actions and land relations of contemporary smallholders and rural populations, without attempting the delicate task of relating these patterns to Indigenous or pre-colonial systems. None of these studies, therefore, provide direct evidence that pre-colonial Indigenous societies significantly altered Araucaria Forests.

Two of the studies that have been cited most frequently in support of anthropogenic Araucaria Forests are dos Reis et al. (2014) and Bitencourt and Krauspenhar (2006). Both bring together disparate strands of existing data – dos Reis and colleagues from ecology, archaeology, palaeoecology and ethnobotany, Bitencourt and Krauspenhar focusing more closely on palaeoecology and archaeology – to identify correlations suggestive of human influence on the ecosystems. dos Reis et al. (2014) highlight a range of reasons why South America’s two Araucaria forests should be expected to have high cultural value, clearly demonstrating why it is reasonable – even sensible – to suppose that the southern Jê significantly manipulated Brazil’s Araucaria Forests. The same conclusions were drawn by Noelli (2000) from archaeological and ethnographic evidence. In many ways, dos Reis et al. (2014) builds on the more focused observations of Bitencourt and Krauspenhar (2006), who were among the first to highlight the accumulating archaeological and palaeoecological evidence that could indicate a potential human role in Araucaria Forest expansions in the Common Era. Such a relationship had been hypothesised decades previously (Aubreville, 1948), but the authors were careful to note that they were only identifying correlations, writing cautiously of “**Possible** prehistoric anthropogenic effect on *Araucaria angustifolia* […] expansion during the late Holocene” (emphasis added). Dos Reis et al. (2014), Noelli (2000) and Bitencourt and Krauspenhar (2006) provide ample evidence demonstrating the eminent plausibility of the southern Jê having shaped Araucaria Forests in the pre-colonial period, but they do not prove that it occurred.

Studies focusing on genetics have contributed to the debate around pre-colonial human impacts on Araucaria Forests. Vasconcellos et al. (2024) use DNA analyses and ecological niche modelling to describe the current spatial genetic structure of *A. angustifolia* and infer its history over 130,000 years. They, like Lauterjung et al. (2018) and Stefenon et al. (2019) before them, identify marked genetic homogeneity in the southern population over long distances, which could have been caused by rapid recent expansions from refugia and/or human seed dispersal. Vasconcellos et al. (2024) note the difficulty of explaining such widespread patterns via natural seed dispersal alone, though whether they could be feasibly explained by plausible pre-colonial human population patterns is not explored. In their phylogeographic study, Lauterjung et al. (2018) attempt to demonstrate that pre-colonial human actions are needed to explain *A. angustifolia’s* contemporary distribution, and accordingly this study is quite frequently cited to this effect. Focusing on a putative forest refugium in the highlands, the authors note that the area’s palaeoecological records only show forest expansion in the last 4,000 years, and calculate that this would not have been long enough for Araucaria trees to travel from there to their westernmost extent without human assistance. However, fossil pollen evidence shows that *A. angustifolia* populations have been present in the west of their contemporary range for most of the last 12,000 years (Bertoldo et al., 2014), and ecological niche modelling has cast doubt on the idea of the species having been confined to a single refugium at any point in millennia (Vasconcellos et al., 2024; Wilson et al., 2021). In sum, while these studies identify patterns in *A. angustifolia* populations that may well be genetic legacies of pre-colonial human management, their true causes – and the mechanisms by which they could have arisen – have not been clearly attributed.

Pereira Cruz et al. (2020) set out to evaluate floristic legacies of past Southern Jê and Guarani land use in Southern Brazil. The authors modelled the potential distributions of archaeological sites from each tradition, then compared these predictions against floristic data from the Santa Catarina Forest Floristic Inventory (Vibrans et al., 2020, IFFSC; 2010). However, the study compares floristic patterns between – rather than within – the two archaeological groups, so the results simply reflect the spatial overlap of the studied groups with different vegetation types. Southern Jê sites and Araucaria Forests are most associated with the highlands, Guarani sites and Atlantic Rainforest are most associated with the coastal lowlands, and major rivers, with associated Seasonally Deciduous Forest, had secondary importance for both groups. Consequently, the gradient of species associated with southern Jê and Guarani archaeological sites (Pereira Cruz et al., 2020, fig. 7) almost perfectly mirrors the IFFSC’s lists of the most important species in Araucaria Forest (southern Jê), Atlantic Rainforest (Guarani) and Seasonally Deciduous Forest (shared) (Schorn et al., 2012). In short, this study shows which forest ecosystems the southern Jê and Guarani were most likely to live among, but it does not – and by design cannot – show any lasting floristic legacies associated with their occupations.

The final study in this review, Robinson et al. (2018), provides by far the strongest direct evidence to date for pre-colonial Indigenous expansion of/influence on Araucaria Forests. It is, accordingly, one of the studies most frequently cited to support claims of Araucaria Forest domestication.

The authors focus on two areas (shown in fig. S1.1 and main text fig. 1d): Lages, which has little known archaeological evidence of human occupation, and Campo Belo do Sul, where past southern Jê occupation was intense. (These are the first palaeoecological records from the latter area.) In Lages, forest areas occur along valley bottoms and on shaded slopes; forests not matching this pattern only made up 6% of the area in 1966, whereas they cover 33% of the Campo Belo do Sul area. To try and explain these differences in forest distribution, the authors examine stable carbon isotopes (δ^13^C) from transects of soil pits across plateaus and valleys in both areas. In Lages there is little change from the beginning of the record at about 7,500 cal BP – the shaded slope and riparian area are dominated by C3 vegetation (most likely forest) and the exposed slope and plateau by C4 plants (grassland). In Campo Belo do Sul, transitions from C4-dominated to C3-dominated vegetation occur, in all parts of the transects, starting (where dates are available) around 1,000-1,200 cal BP (our S3.1). Noting that an independent rainfall proxy record (Bernal et al., 2016) shows this period saw no major change (certainly no increase) in precipitation (our fig. 3a), but that it did coincide with increases in archaeological evidence (our fig. 3c), the authors attribute these expansions – and Campo Belo do Sul’s out-of-niche forest areas – to the southern Jê.

The key assumption on which these conclusions rest is that the patterns at Lages and Campo Belo do Sul should be the same in the absence of pre-colonial human influence. This assumption may not be valid. The Campo Belo do Sul and Lages sites are approximately 60 km apart and Lages is at considerably higher elevation (ca. 1100 m compared to approximately 750-980 m; fig. S1.1). Whereas Lages sits well within relatively intact Campos vegetation, Campo Belo do Sul is closer to or within areas of Araucaria Forest that were originally continuous but which have seen significant habitat loss (and had already done so by 1966; fig. S1.1). The vegetation model trained on forest and grassland distributions at Lages may not therefore be transferrable to these different conditions in Campo Belo do Sul, and the aerial photos used to assess forest distribution in the landscape in 1966 did not capture their near-natural state. The Campo Belo do Sul sites are also the first palaeoecological proxy records in this part of the plateau, so it is unclear whether they should be expected to exhibit the same changes through time as Lages or other sites. The Lages valley has a clear and consistent pattern of forest in riparian areas and shaded slopes, and grassland on exposed slopes and plateau. By contrast, no studied part of the Campo Belo do Sul valleys is forested in the earliest (undated) parts of the records, and all parts of the area’s Mata Queimada and Heraldo valleys start off as grassland and become more C3-dominated through time. This may mean that the expansion of Araucaria Forest onto the plateau tops in Campo Belo do Sul – interpreted by the authors as a clear signal of human landscape transformation – was instead part of a general trend for all parts of this area’s landscape to move from grassland to forest.

These caveats do not necessarily invalidate Robinson et al.’s (2018) findings or the implications they have for pre-colonial impacts on Araucaria Forests by southern Jê people. They do, however, mean that the study should not be interpreted as delivering the definitive evidence that fully resolves the issue of southern Jê alteration of Araucaria Forests. An important next step is to account for potential natural differences between Lages and Campo Belo do Sul – both in their spatial distribution of Araucaria Forest and in their vegetation histories – to help clarify the extent to which the observed patterns can be confidently attributed to human actions.


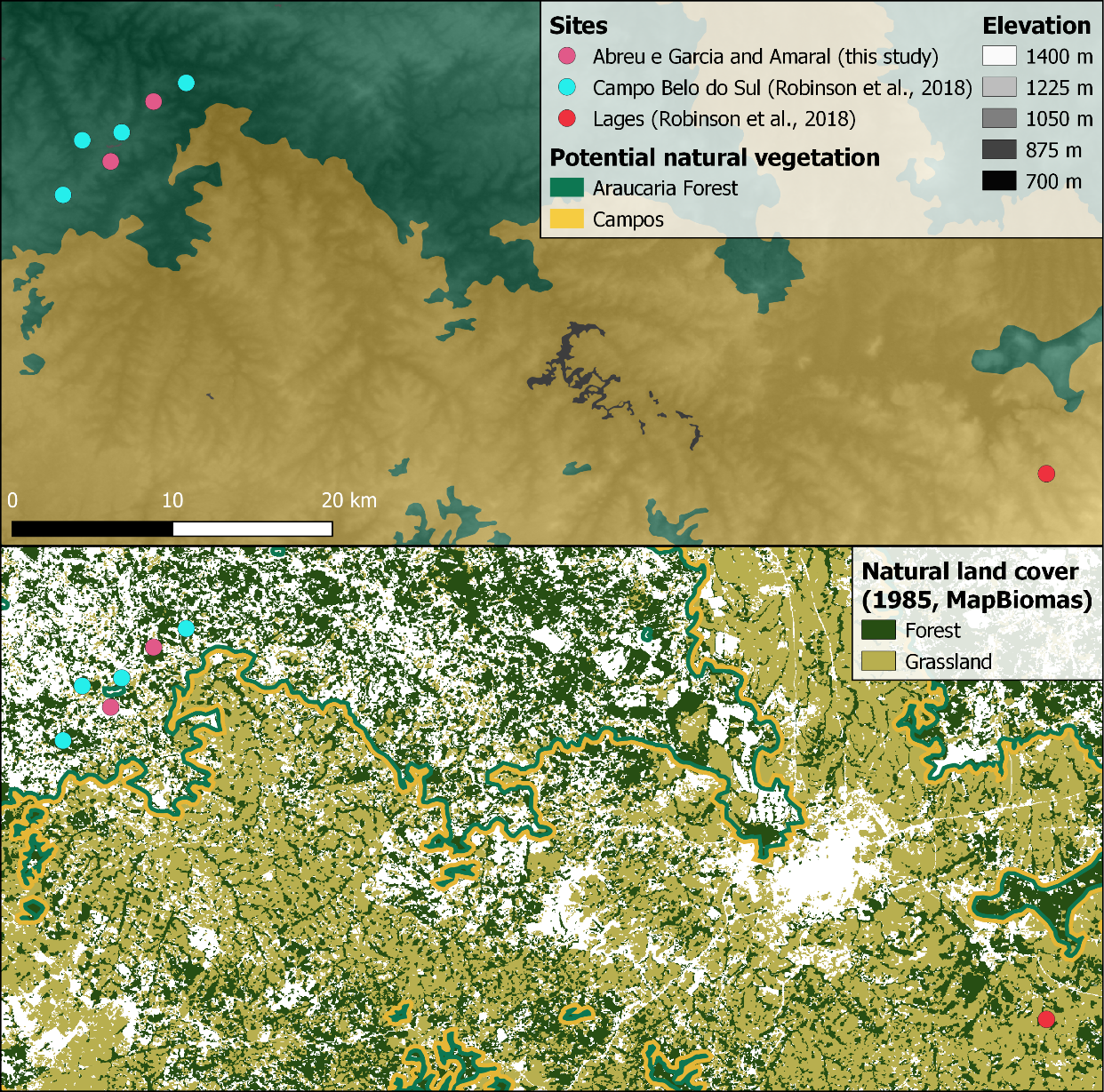


*Fig. S1.1: The environmental context for the Campo Belo do Sul and Lages study areas: their elevation and potential natural vegetation (top) and the distribution of modern vegetation remnants (below, using natural grassland and forest in 1985 from MapBiomas* (Souza et al., 2020)*).*

Taking these studies together it becomes clear that, although the view of modern Araucaria Forests as pre-colonial Indigenous cultural landscapes is increasingly widespread, the direct evidence underpinning it remains equivocal. Believing that such evidence will emerge remains a fundamentally well-reasoned stance, for all the reasons highlighted by dos Reis et al. (2014) and others, and it is plausible – perhaps even probable – that Indigenous people had widespread, significant and enduring impacts on Araucaria Forest. But our review of the current evidence undoubtedly shows that such connections have yet to be definitively proven. The findings of Robinson et al. (2018) come closest to settling the debate, but the uncertainties we highlight around the study’s core assumption emphasise how further research can help better account for potential natural drivers of the observed changes, integrate a wider array of the existing data, and provide clarity on when, where, how, and to what extent the southern Jê shaped Brazil’s Araucaria Forests over recent millennia. The research we present in our study aims to do just this.

# S2. Supplementary methods

## S2.1. Palaeoclimate data

Median Absolute Deviation (MAD) is a robust measure of climate (here, precipitation) variability, identifying sudden or large-amplitude deviations from a moving normal. Sub-annual trace element ratio data from Botuverá (Bernal et al., 2016; https://www.ncdc.noaa.gov/paleo-search/study/21060) was first aggregated to decadal resolution, since MAD is sensitive to the number of observations in a time window. (The record’s relatively coarse, multi-decadal stable isotope ratios were therefore not used.) Observations that were at least three times above or below the 100-year moving median proxy value were treated as outliers; Riris and Arroyo-Kalin (2019) identify this as a conservative threshold for extreme outliers. The anomaly count in 100-year bins was summed across the three trace element proxies, and this anomaly count was compared to the standard deviation of the dataset mean. Riris and Arroyo-Kalin (2019) suggest time bins with an anomaly count more than two standard deviations above the mean represent periods with significantly more variable rainfall than normal.

## S2.2. Ecological niche modelling

### S2.2.1. Locality data

For ecosystem-level models, a regular grid of points spaced 0.1^o^ apart was set within the training data extent, and this was compared with data on remnant natural vegetation areas. For this, we used data from MapBiomas collection 4 (Souza et al., 2020) (actual vegetation) and Mapeamento de Recursos Naturais do Brasil (MRNB; IBGE - Instituto Brasileiro de Geografia e Estatística, 2018) (potential natural vegetation). The MapBiomas project is a multi-institutional initiative to generate annual land use land cover maps based on automatic classification processes applied to satellite images (the complete project description can be found at <http://mapbiomas.org>). We used MapBiomas data from 1985, since it includes greater areas of natural vegetation than more recent data points (though still much reduced from pre-colonial baselines), aggregated from 30m to 100m resolution. Grid points were considered ‘present’ if they: a) occurred in the Atlantic Forest region; b) were classed as ‘natural forest’ (for Araucaria Forest) or ‘natural grassland’ (for Campos) in the MapBiomas data; and c) occurred in relevant areas (including ecotones) under the MRNB classification – E/EM/EN/M for Campos and E/EM/M/NM/OM/SM for Araucaria forest, where ‘E’ is ‘estepe’ (Campos), ‘M’ is ‘floresta ombrófila mista’ (Araucaria Forest), and letter pairs are ecotones with other vegetation types. Araucaria Forest’s natural distribution extends into north-eastern Argentina, which was not covered by either MRNB or MapBiomas for 1985; here we considered points within the Araucaria Moist Forests WWF ecoregion (Olson et al., 2001) with medium or high forest landscape integrity (Forest Landscape Integrity Index >6) (Grantham et al., 2020) as presences.

We used two different approaches of translating non-presence grid points to absences for the modelling algorithms that required them (fig. S2.2.1.1, S2.2.3). The ‘all absences’ approach interpreted any point that did not qualify as a presence as a true absence. This included points that lack natural vegetation because of recent human-caused habitat change, rather than topoclimatic unsuitability, so could introduce inaccuracies. We therefore also employed a ‘natural absences’ approach, where the only non-presence points interpreted as absences were those that fell outside the relevant MRNB vegetation region or occurred in areas of other natural vegetation in the MapBiomas data. In contrast to the all-absence approach, the natural-absence approach is less affected by anthropogenic habitat loss, but does omit points where the absence of Campos or Araucaria Forest is the result of real (and informative) topoclimatic preference. We include both strict and permissive (respectively) approaches, acknowledging that each likely captures a different subset of ‘true’ absences and that the ‘correct’ answer, if it could be found, likely lies somewhere between the two.


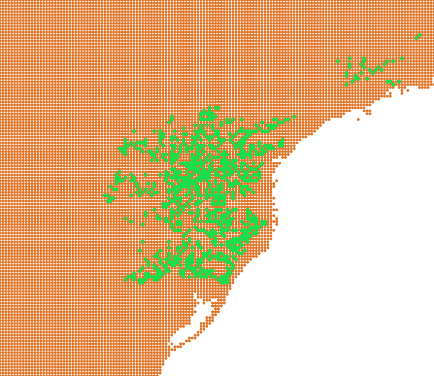

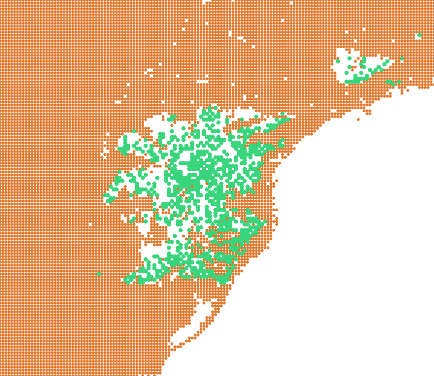

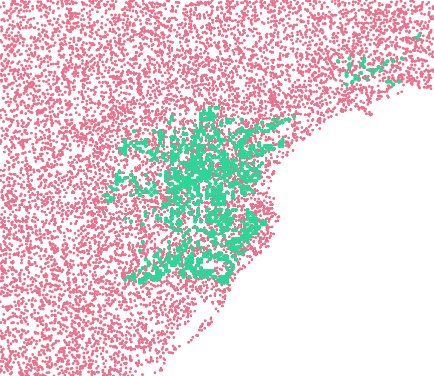


*Fig. S2.2.1.1: Maps showing the approaches to presence (green), absence (orange) and background (purple) data points, illustrated using Araucaria Forest: all absences (left), natural absences (centre), and random background (right). The presence-absence approaches (left and centre) were used to train ecosystem-level Random Forest models, and the presence-background approach (right) was used for the Maxent algorithm in both ecosystem- and species-level models (but note that ecosystem- and species-level Maxent models used different sources of presence points).*

For the modelling of key species, we identified 10 important Campos species (Andrade et al., 2019) and 20 tree species with high importance in different parts of Araucaria Forest’s range (Klein, 1975; Oliveira-Filho et al., 2014; Roderjan et al., 2002; Schorn et al., 2012; Universidade Federal de Santa Maria, 2001) (table S3.2.1.1). Additionally, we elected to model five further highly important species from each of Atlantic Rainforest and Seasonally Deciduous Forest (Schorn et al., 2012) since, in the present day, these forests sit on a floristic continuum rather than being sharply divided and their compositions (and therefore distinctions between them) may have differed significantly in the past (Wilson et al., 2021). Analysing this suite of species allows us to discern changes both within and between Campos, Araucaria Forest, and other formations, including the development of forest communities that may not fit well with modern forest classifications. Pre-processed locality data for tree species was taken from Zweiner et al. (2017), derived in turn from the Neotropical Tree Communities database (de Lima et al., 2015). Locality data for Campos species was downloaded from SiBBr/ALA (<https://bit.ly/2PqTd5X>) and coordinates were cleaned using the ‘CoordinateCleaner’ R package (Zizka et al., 2019). Following Brown et al. (2020), we thinned these occurrence records so that only one coordinate remained within every 20km radius, using the R package ‘spThin’ (Aiello-Lammens et al., 2015).

| **Species** | **Pres. Pts.** | **AUC** | **Boyce Index (Spearman correlation)** | **Seasonally Deciduous Forest** | **Araucaria Forest** | **Campos** | **Atlantic Rainforest** |
| --- | --- | --- | --- | --- | --- | --- | --- |
| *Alchornea triplinervia* | 184 | 0.807 | 0.986 |  |  |  | Y |
| *Alsophila setosa* | 74 | 0.827 | 0.945 |  |  |  | Y |
| *Andropogon lateralis* | 86 | 0.854 | 0.891 |  |  | Y |  |
| *Araucaria angustifolia* | 79 | 0.851 | 0.980 |  | Y |  |  |
| *Baccharis crispa* | 234 | 0.821 | 0.949 |  |  | Y |  |
| *Blepharocalyx salicifolius* | 173 | 0.805 | 0.969 |  | Y |  |  |
| *Casearia sylvestris* | 370 | 0.754 | 0.949 | Y |  |  |  |
| *Cedrela fissilis* | 211 | 0.758 | 0.954 | Y |  |  |  |
| *Chaetogastra gracilis* | 66 | 0.734 | 0.807 |  |  | Y |  |
| *Cinnamodendron dinisii* | 35 | 0.874 | 0.831 |  | Y |  |  |
| *Clethra scabra* | 166 | 0.837 | 0.985 |  | Y |  |  |
| *Cupania vernalis* | 238 | 0.770 | 0.949 | Y | Y |  |  |
| *Cyathea phalerata* | 98 | 0.889 | 0.871 |  |  |  | Y |
| *Dicksonia sellowiana* | 104 | 0.880 | 0.962 |  | Y |  |  |
| *Gamochaeta americana* | 50 | 0.833 | 0.855 |  |  | Y |  |
| *Hieronyma alchorneoides* | 57 | 0.906 | 0.922 |  |  |  | Y |
| *Ilex paraguariensis* | 180 | 0.831 | 0.974 |  | Y |  |  |
| *Lithrea brasiliensis* | 79 | 0.819 | 0.909 |  | Y |  |  |
| *Luehea divaricata* | 241 | 0.756 | 0.975 | Y | Y |  |  |
| *Machaerium stipitatum* | 156 | 0.766 | 0.904 | Y |  |  |  |
| *Matayba elaeagnoides* | 194 | 0.719 | 0.994 |  | Y |  |  |
| *Mimosa scabrella* | 120 | 0.837 | 0.919 |  | Y |  |  |
| *Nectandra lanceolata* | 191 | 0.811 | 0.938 | Y |  |  |  |
| *Nectandra megapotamica* | 279 | 0.798 | 0.993 | Y | Y |  |  |
| *Ocotea porosa* | 103 | 0.901 | 0.907 |  | Y |  |  |
| *Ocotea puberula* | 226 | 0.773 | 0.959 | Y | Y |  |  |
| *Ocotea pulchella* | 269 | 0.765 | 0.970 |  | Y |  |  |
| *Parapiptadenia rigida* | 166 | 0.788 | 0.965 | Y |  |  |  |
| *Paspalum notatum* | 128 | 0.569 | 0.879 |  |  | Y |  |
| *Paspalum plicatulum* | 98 | 0.685 | 0.801 |  |  | Y |  |
| *Paspalum pumilum* | 50 | 0.894 | 0.952 |  |  | Y |  |
| *Piptochaetium montevidense* | 64 | 0.905 | 0.565 |  |  | Y |  |
| *Podocarpus lambertii* | 84 | 0.795 | 0.879 |  | Y |  |  |
| *Psychotria vellosiana* | 180 | 0.867 | 0.877 |  |  |  | Y |
| *Schizachyrium microstachyum* | 88 | 0.787 | 0.897 |  |  | Y |  |
| *Schizachyrium tenerum* | 50 | 0.887 | 0.851 |  |  | Y |  |
| *Sebastiania commersoniana* | 142 | 0.793 | 0.963 |  | Y |  |  |
| *Sloanea lasiocoma* | 73 | 0.836 | 0.924 |  | Y |  |  |
| *Syagrus romanzoffiana* | 100 | 0.815 | 0.812 | Y |  |  |  |
| *Vernonanthura discolor* | 83 | 0.894 | 0.976 |  | Y |  |  |

*Table S2.2.1.1: the 40 modelled species, the number of presence points used for training the Maxent models, their model evaluation scores, and the ecosystems in which each species has the most phytosociological importance.*

### S2.2.2. Environmental data

Environmental data is drawn from two sources: CHELSA-TraCE21k downscaled palaeoclimate data ([www.doi.org/10.16904/envidat.211](http://www.doi.org/10.16904/envidat.211)) (Karger, 2021; Karger et al., 2017; Yannic et al., 2020) (both species- and ecosystem-level modelling) and the ASTER Global Digital Elevation Model (v3) (NASA et al., 2019) (ecosystem-level modelling only). The 30m ASTER data was aggregated to 100m resolution, then used to produce two layers, a topographic position index (TPI) and a measure of exposure to peak insolation. The topographic position index was calculated following Robinson et al. (2018), taking the difference between a cell’s elevation and the average of its neighbours in a five-cell (500m) radius then standardising the output so it had a mean of 0 and standard deviation of 1. Positive TPI values indicate relatively high topographic position (e.g. ridges and plateaus) and negative values indicate depressions and valleys. Exposure to peak insolation (azimuth of 315°; McCune, 2007; McCune and Keon, 2002) was calculated as in Ashcroft et al. (2008) and Wilson et al. (2019).

For ecosystem-level modelling of Araucaria Forest and Campos, CHELSA-TraCE21k data was downscaled to 100m resolution through bilinear interpolation. Species-level modelling used CHELSA-TraCE21k data at its native 30” (ca. 800m) resolution. The CHELSA-TraCE21k palaeoclimate dataset consists of palaeoclimate data downscaled using the CHELSA algorithm (Karger et al., 2017; Karger and Zimmermann, 2019) from 2.5° resolution to 30” (ca. 800m) (Karger et al., 2021). We projected models to 13 time slices covering 500-year intervals from 6,000 BP to the present; each period is averaged over 100 years. Compared to the CHELSA climatology (1979-2013), the CHELSA-TraCE21k data underestimates some sharp, ecologically important temperature gradients in our study area (such as across the highlands’ eastern escarpment) by up to 2°C, with these deviations remaining consistent through the last 6,000 years (fig. S2.2.2.1). We used a change factor approach to correct for these discrepancies, generating a correction layer (calculated as climatology/TraCE) for each monthly modern-day temperature layer and multiplying this with past CHELSA-TraCE21k temperature layers. Because they are less directly linked to elevation, precipitation layers were not corrected.


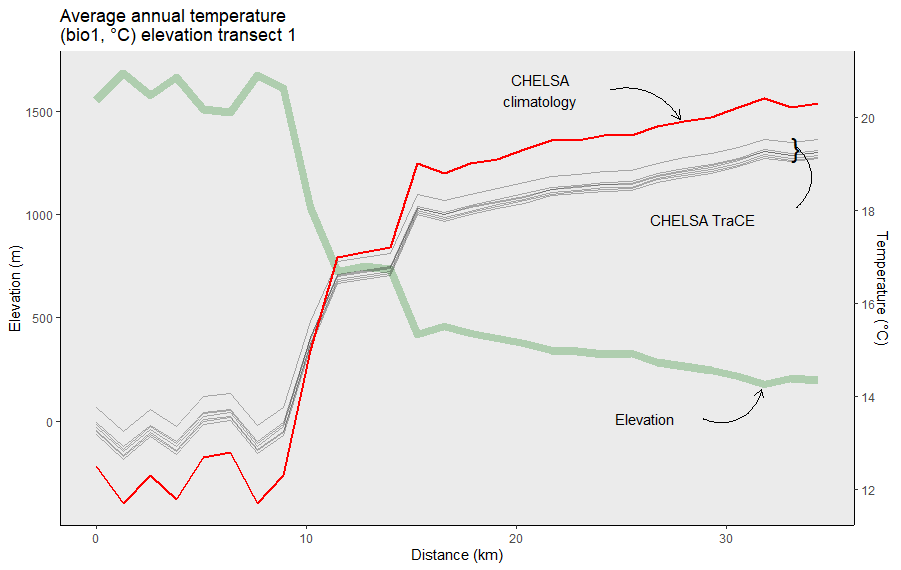

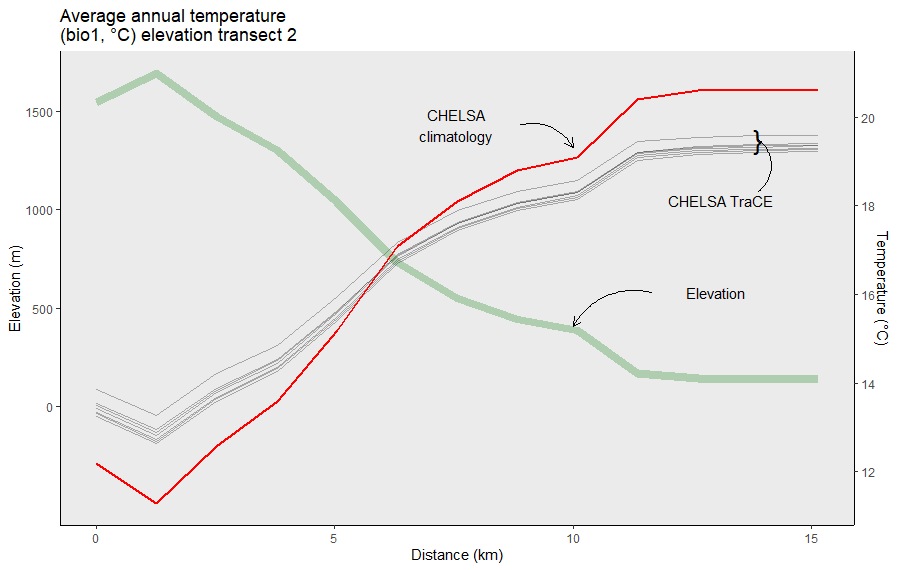


*Fig. S2.2.2.1: CHELSA-TraCE21k systematically underestimates temperature differences across steep elevation gradients (green) at two locations on the highlands’ eastern escarpment. Average annual temperatures from CHELSA-TraCE21k from 6,000 BP to present (black) are compared to those from CHELSA’s 1979-2013 climatology (red).*

Bioclimatic variables were then generated from monthly precipitation and corrected temperature layers using the ‘dismo’ R package. Best practice in species distribution modelling requires that variables are biologically relevant and not closely correlated (Fourcade et al., 2017). We evaluated the 19 bioclimatic variables’ multicollinearity using variance inflation factors, using the ‘vifcor’ function from the ‘usdm’ R package (Naimi et al., 2014) and a correlation threshold of 0.75. This yielded eight ecologically relevant variables which describe the general trends and extremes of rainfall and temperature in our study area: bio2 (annual mean diurnal temperature range), bio4 (temperature seasonality), bio5 (maximum temperature of the warmest month), bio8 (average temperature of the wettest quarter), bio9 (average temperature of the driest quarter), bio12 (annual precipitation), bio14 (precipitation of the driest month), and bio18 (precipitation of the warmest quarter).

### S2.2.3. Modelling

The F-ratios from the Boyce Index evaluation describe the predicted-to-expected ratios of different habitat suitability classes in model outputs. An F-ratio of 0.5 indicates there are half as many presence points in a predicted habitat suitability class as would be expected by chance (i.e. low-suitability habitat), an F-ratio of 1 suggests there are as many presences as would be expected (marginal habitat), an F-ratio of 2 indicates there are twice as many as would be expected (good habitat), etc. To compare our modelled predictions of Araucaria Forest and Campos, we converted habitat suitability values to F-ratios, reclassified areas with F-ratios <1 to zero (i.e. sub-marginal habitat to absences), incremented all values by 1 and log10 transformed them. We then subtracted the Campos values from those of Araucaria Forest to show changes in their relative suitability through time and space; this approach has particular value because large parts of our study area (including most palaeoecological proxy sites) show little change in their predictions of the presence or absence of either ecosystem over the last 6,000 years.

To analyse the changing compositions of southern Brazil’s ecosystems through the last 6,000 years, we clustered the outputs of our individual species models into a smaller number of predicted assemblages (cf. Wilson et al., 2021). We first used k-means clustering in SAGA-GIS (k=10) on each time slice’s 40 species-level habitat suitability maps, which resulted in 130 predicted assemblages (13 time slices with 10 clusters each). Because each time slice was clustered independently, floristically similar clusters are likely to have been identified at different times. To combine these similar clusters/assemblages, we used the R package ‘pvclust’ to perform hierarchical clustering with p-values calculated over 10,000 bootstrap replications.

Four statistically significant clusters were identified (96-100% support), one of which encompassed most of the range of Araucaria Forest and Seasonally Deciduous Forest; this we split into four further subgroups (assemblages 1, 2, 4 and 6; 70-100% support) to better analyse ecologically meaningful compositional changes. The modelled assemblages relate to: Atlantic Rainforest (assemblage 3); Seasonally Deciduous Forest (assemblage 2); Araucaria-Seasonally Deciduous Forest ecotones (assemblage 4); Araucaria Forest with some Campos (assemblage 1); Campos with (higher-elevation) Araucaria Forest (assemblage 5); and high-elevation Araucaria Forest and Campos, with some more cold-adapted Atlantic Rainforest species (assemblage 7). Assemblage 6 is mostly representative of Araucaria Forest, containing elements of Araucaria Forest, Campos and Seasonally Deciduous Forest, but it is the cluster least well represented by our modelled species – in the modern day much of its distribution lies outside the Atlantic Forest region our study focuses on. The assemblages, their modern distributions and their species compositions are illustrated in Fig. S2.2.3.1.


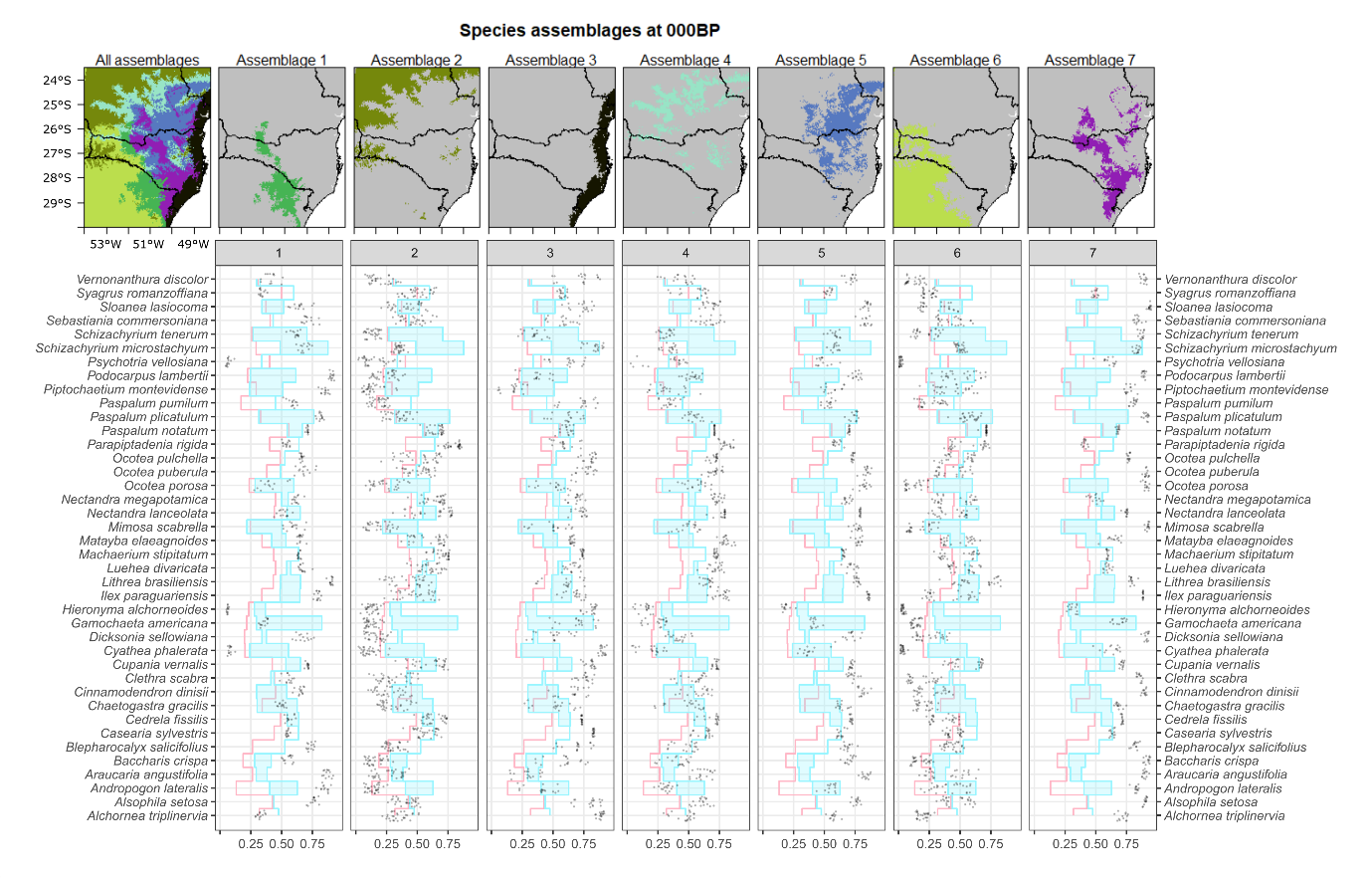


*Fig. 2.2.3.1: the predicted assemblages produced by the clustering analysis, their distributions in the present day, and their species-level compositions. In the lower part of the plot, dots show the modelled habitat suitability values (from low to high, left to right) for each species (rows) within each assemblage; red lines show the values above which 90% of species presence locations were found; and blue lines/zones indicate marginal habitat (the lowest and highest values with an F-ratio of 1). Species with more dots further to the right (left) of the red and blue lines/zones are more likely to be present in (absent from) a given assemblage.*

## S2.3. Palaeo-vegetation proxy synthesis

Where possible, we used raw data on proxy values. In some cases these were included in original publications or in data repositories (specificallyhttps://doi.pangaea.de/10.1594/PANGAEA.872561 <https://doi.pangaea.de/10.1594/PANGAEA.872851>, <https://doi.pangaea.de/10.1594/PANGAEA.872855>, <https://doi.pangaea.de/10.1594/PANGAEA.888010>, <https://doi.org/10.17632/7gxn9g87cf.1>, <https://doi.org/10.21233/n39h81>, <https://doi.org/10.21233/n3jm87>, <https://doi.org/10.21233/n33q4q>, <https://doi.org/10.21233/n34924>, <https://doi.org/10.21233/n3848x>, <https://doi.org/10.25921/mrjz-sa96>), and co-author HB provided raw data for several additional sites (see table S2.3.1 for all data sources). For the region’s remaining records, we digitised proxy diagrams using Fiji/ImageJ (Schindelin et al., 2012) to extract: subsample depths; the percentages of *Araucaria* pollen and the most relevant forest pollen group; counts or percentages of any potential cultigen pollen (e.g. *Zea mays*); δ^13^C isotope values; and counts or concentrations of charcoal or charred particles. We constructed new age-depth models for all records using the ‘rbacon’ package (Blaauw et al., 2020; Blaauw and Christen, 2011) and the SHCal20 (Hogg et al., 2020) and Marine20 (Heaton et al., 2020) calibration curves for terrestrial and marine sites, respectively. Where sites had no date within the last 1,000 years, we followed Wilson et al. (2021) and added a surface date of -54 ± 15 cal years BP (i.e. 1991-2019 AD), a period which encompasses the extraction dates of almost every core in our synthesis. We used rbacon’s suggested values for memory and accumulation priors, and included hiatuses where original publications indicated that these were present.

The requirement for included records to have two dates represents a comparatively low bar for chronological quality (Flantua et al., 2016; Smith and Mayle, 2017; Wilson et al., 2021), but even so several records (mainly δ^13^C isotope records or sites from older studies) failed to meet it. Raising the requirement to three dates would have excluded 10 more sites, including some with the most significant and relevant findings in the region. The resulting temporal uncertainty in many records of past Araucaria Forest-Campos dynamics has been little considered in the past, yet has a major bearing on our ability to interpret the timings of vegetation changes and their links with potential human and climatic drivers. To address this and visualise the uncertainty in their age-depth models, we plotted records’ proxy values against ten random iterations of their age-depth models. This approach helps to avoid the false impression of chronological precision that can arise when simply plotting proxy values against mean or median dates, and provide a more accurate – if less precise – picture of the timings of vegetation changes.


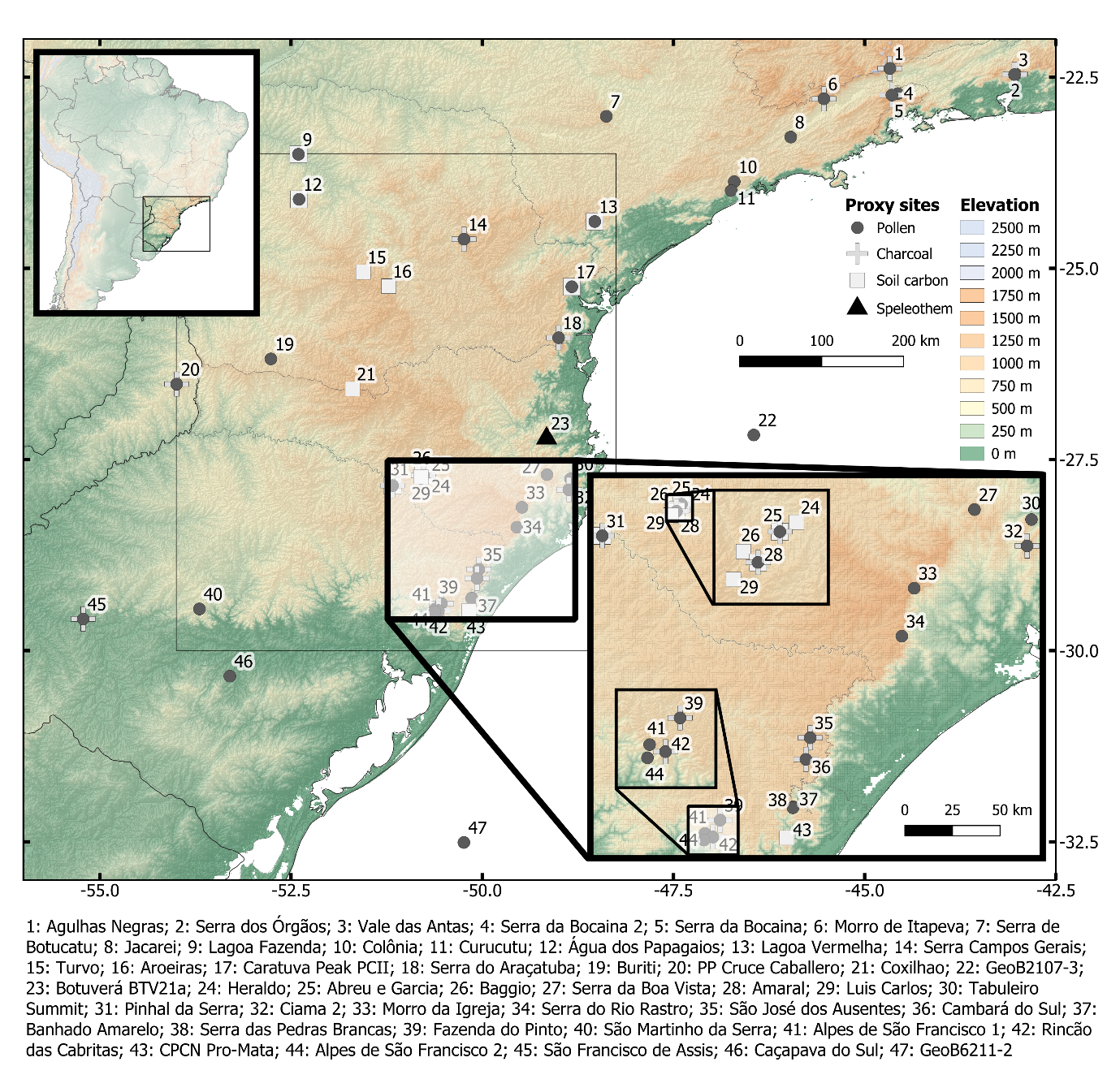


*Fig. S2.3.1. Map of the synthesised palaeoecological sites and the proxies they record.*

| **Site no.** | **Site name** | **Site code** | **Source(s)** | **Latitude** | **Longitude** | **Source** |
| --- | --- | --- | --- | --- | --- | --- |
| 1 | Agulhas Negras | AguNe | (Behling et al., 2020) | -22.385 | -44.670 | HB |
| 2 | Serra dos Órgãos | SdOrg | (Behling and Safford, 2010) | -22.458 | -43.028 | HB |
| 3 | Vale das Antas | ValAn | (de Oliveira Portes et al., 2020) | -22.466 | -43.041 | Digitised |
| 4 | Serra da Bocaina 2 | SdBc2 | (Behling et al., 2007) | -22.714 | -44.567 | HB |
| 5 | Serra da Bocaina | SdBoc | (de Oliveira Portes et al., 2018) | -22.734 | -44.644 | Digitised |
| 6 | Morro de Itapeva | MdIta | (Behling, 1997a) | -22.783 | -45.533 | Neotoma |
| 7 | Serra de Botucatu | SdBot | (Bissa and de Toledo, 2015) | -23.012 | -48.378 | Digitised |
| 8 | Jacarei | Jacar | (Garcia et al., 2004) | -23.283 | -45.967 | Digitised |
| 9 | Lagoa Fazenda | LaFaz | (Fernandes, 2009) | -23.509 | -52.404 | Paper/ digitised |
| 10 | Colônia | Colon | (Ledru et al., 2009) | -23.867 | -46.706 | Pangaea |
| 11 | Curucutu | Curuc | (Pessenda et al., 2009) | -23.983 | -46.746 | Paper/ digitised |
| 12 | Águas dos Papagaios | AgPap | (Ladchuk et al., 2016; Luz et al., 2019) | -24.098 | -52.396 | Paper/ digitised |
| 13 | Lagoa Vermelha | LaVer | (Saia, 2006) | -24.388 | -48.529 | Paper/ digitised |
| 14 | Serra Campos Gerais | SCaGe | (Behling, 1997b) | -24.619 | -50.240 | Neotoma |
| 15 | Turvo | Turvo | (Rasbold et al., 2016) | -25.049 | -51.556 | Paper |
| 16 | Aroeiras | Aroei | (Silva, 2018) | -25.235 | -51.224 | Digitised |
| 17 | Caratuva Peak PCII | CarP2 | (Scheer et al., 2014) | -25.241 | -48.829 | Paper/ digitised |
| 18 | Serra do Araçatuba | SdAra | (Behling, 2006) | -25.908 | -49.002 | HB |
| 19 | Buriti | Burit | (Bertoldo, 2010; Bertoldo et al., 2014) | -26.185 | -52.764 | Digitised |
| 20 | PP Cruce Caballero | CruCa | (Gessert et al., 2011) | -26.515 | -53.996 | HB |
| 21 | Coxilhao | Coxil | (Silva, 2018) | -26.578 | -51.695 | Digitised |
| 22 | GeoB2107-3 | GeB23 | (Gu et al., 2017) | -27.180 | -46.450 | Pangaea |
| 23 | Botuverá | Botuv | (Bernal et al., 2016) | -27.223 | -49.158 | NOAA |
| 24 | Heraldo | Heral | (Robinson et al., 2018) | -27.659 | -50.722 | Paper |
| 25 | Abreu e Garcia | AbGar | This study | -27.671 | -50.743 | This study |
| 26 | Baggio | Baggi | (Robinson et al., 2018) | -27.696 | -50.788 | Paper |
| 27 | Serra da Boa Vista | SdBoV | (Behling, 1995, 1993) | -27.700 | -49.154 | Neotoma |
| 28 | Amaral | Amarl | This study | -27.709 | -50.770 | This study |
| 29 | Luis Carlos | LuiCa | (Robinson et al., 2018) | -27.730 | -50.801 | Paper |
| 30 | Tabuleiro Summit | TabSu | (Behling and de Oliveira, 2018) | -27.754 | -48.844 | HB |
| 31 | Pinhal da Serra | PindS | This study | -27.842 | -51.173 | This study |
| 32 | Ciama 2 | Ciam2 | (Jeske-Pieruschka et al., 2013) | -27.897 | -48.868 | HB |
| 33 | Morro da Igreja | MdIgr | (Behling, 1995, 1993) | -28.127 | -49.481 | Neotoma |
| 34 | Serra do Rio Rastro | SdRRa | (Behling, 1995, 1993) | -28.387 | -49.548 | Neotoma |
| 35 | São José dos Ausentes | SJdAu | (Jeske-Pieruschka et al., 2010) | -28.938 | -50.044 | HB |
| 36 | Cambará do Sul | CamSu | (Behling et al., 2004) | -29.055 | -50.068 | Pangaea |
| 37 | Banhado Amarelo | BanAm | (Scherer and Lorscheitter, 2014) | -29.313 | -50.137 | Digitised |
| 38 | Serra das Pedras Brancas | SPedB | (Spalding and Lorscheitter, 2015) | -29.318 | -50.138 | Digitised |
| 39 | Fazenda do Pinto | FazdP | (Behling et al., 2001) | -29.385 | -50.533 | HB |
| 40 | São Martinho da Serra | SMadS | (Bauermann et al., 2008) | -29.456 | -53.698 | HB/ digitised |
| 41 | Alpes de São Francisco 1 | AdSF1 | (Scherer and Lorscheitter, 2014) | -29.457 | -50.616 | Digitised |
| 42 | Rincão das Cabritas | RidCa | (Jeske-Pieruschka and Behling, 2012) | -29.476 | -50.573 | HB |
| 43 | CPCN Pro Mata | ProMa | (Dümig et al., 2008; Silva and Anand, 2011) | -29.481 | -50.172 | Digitised |
| 44 | Alpes de São Francisco 2 | AdSF2 | (Leonhardt and Lorscheitter, 2010) | -29.493 | -50.622 | Digitised |
| 45 | São Francisco de Assis | SFdAs | (Behling et al., 2005) | -29.587 | -55.217 | HB |
| 46 | Caçapava do Sul | CacSu | (Behling et al., 2016) | -30.333 | -53.300 | HB |
| 47 | GeoB6211-2 | GeB62 | (Gu et al., 2018) | -32.510 | -50.240 | Mendeley Data |

*Table S2.3.1. Information on the sites used in the palaeoecological synthesis: their number in figs. 1 and S3.3.1; name; code in the Supplementary Data; original publication; coordinates; and data source (paper = tables or supplementary information in the original publication; HB = raw data from Hermann Behling; NOAA, Neotoma and Pangaea, see links above). In some instances, published coordinates have been corrected based on map or image data in the original studies.*

## S2.4. New palaeoecological data

We present new multiproxy palaeoecological data from three sites toward the centre of southern Brazil’s highland plateau. These records provide critical insights into previously un(der)investigated aspects of Araucaria Forest-Campos dynamics in two main ways. Firstly, the most intensely studied area in southern Brazil’s highland region is its south-eastern corner, where a dozen sites lie within 20 km of the plateau’s edge; the new sites are >130 km from the eastern escarpment – further from core Campos areas, closer to more contiguous Araucaria Forest, and in areas predicted to have experienced more dynamic past vegetation change (Wilson et al., 2021). Secondly, the new locations are all extremely close (<1 km) to well-studied archaeological sites in the Canoas-Pelotas basin, which has the most archaeological evidence for pre-colonial southern Jê occupation of anywhere in the highlands (de Souza et al., 2016b). Conversely, the great majority of other palaeo-vegetation proxy records come from areas with far fewer archaeological sites, and no other pollen record has a close link with archaeological evidence.

### S2.4.1. Site overviews

The three new sites – Abreu e Garcia, Amaral, and Pinhal da Serra – are peat bogs in small depressions, which were specifically selected to provide vegetation histories from landscapes known to have been occupied by southern Jê communities.

Abreu e Garcia (27.6710° S, 50.7431° W, 931 m elevation) is a 200 x 40 m bog within a shallow depression, located on a promontory >50 m above the surrounding landscape, and approximately 20 m away from two southern Jê funerary mound and enclosure complexes (MECs) which have been dated to between 500 and 200 years ago (Robinson et al., 2017). The surrounding catchment has gentle slopes ca. 5-10 m high, which are deforested and currently used for cattle pasture and a vineyard. The bog is oriented N-S and drains southward down a steep 30 m escarpment covered in mixed Araucaria woodland. A 50 cm core was collected with a Russian corer.

Amaral (27.7090° S, 50.7702° W, 867 m elevation) is a small 20 x 40 m bog within a shallow depression covered with grasses, sedges, and small woody shrubs. It is located about 800 m from a ‘large, dense, and well-planned’ pit house settlement which was occupied without major abandonment for about 500 years until the late 18^th^ Century (ca. 595-160 cal BP) (de Souza et al., 2016b; de Souza, 2018). The bog is located at the head of a valley and fed by small streams from the surrounded slopes, which are covered in a plantation of non-native pines (*Pinus* sp.). A 65 cm sequence of overlapping cores was collected with a Russian corer.

Pinhal da Serra (27.8421° S, 51.1728° W, 920 m elevation) is a small, circular, 50m diameter bog, located in a horseshoe-shaped basin surrounded by 10 m high slopes and with a single out-flowing stream. The bog is surrounded by agricultural fields and lies approximately 40 km south-west of the Abreu e Garcia and Amaral sites. It is in an archaeologically important area which was occupied from ca. 1,200 cal BP to 300 cal BP, with the site located only 200 m away from a pit house village and 500 m away from an MEC (de Azevedo and Scheel-Ybert, 2020; Iriarte et al., 2013). A 200 cm sequence of overlapping cores was collected with a Russian corer.

### S2.4.2. Radiocarbon dating

| **Site** | **Lab code** | **Depth (cm)** | **Uncalibrated age BP/pMC** | **Calibrated age BP 2σ** |
| --- | --- | --- | --- | --- |
| Abreu e Garcia | Beta-436684 | 4-4.5 | 100.9 pMC ± 0.3 | Modern |
|  | Beta-457741 | 6.5-7 | 890 ± 30 | 728-906 |
|  | Beta-441944 | 10-10.5 | 2,070 ± 30 | 1,942-2,118 |
|  | Beta-457740 | 15-15.5 | 2,370 ± 30 | 2,338-2,490 |
|  | Beta-441945 | 20-20.5 | 4,200 ± 30 | 4,620-4,884 |
|  | Beta-441947 | 30-30.5 | 5,320 ± 30 | 5,997-6,257 |
|  | Beta-436685 | 34-34.5 | 4,930 ± 30 | 5,594-5,720 |
| Amaral | Beta-457742 | 9.5 | 106.7 pMC ± 0.4 | Modern |
|  | Beta-441947 | 15 | 690 ± 30 | 562-678 |
|  | Beta-457743 | 17 | 880 ± 30 | 723-905 |
|  | Beta-457744 | 27 | 1,520 ± 30 | 1,314-1,515 |
|  | Beta-441948 | 30 | 3,710 ± 30 | 3,933-4,150 |
|  | Beta-457745 | 34.5 | 3,220 ± 30 | 3,376-3,482 |
|  | Beta-420641 | 60 | 7,853 ± 30 | 8,547-8,767 |
| Pinhal da Serra | Beta-461673 | 10 | 107.2 pMC ± 0.4 | Modern |
|  | Beta-411674 | 30 | 1,000 ± 31 | 796-959 |
|  | Beta-401728 | 45 | 2,220 ± 30 | 2,146-2,334 |
|  | Beta-405341 | 90 | 9,800 ± 40 | 11,176-11,266 |
|  | Beta-405342 | 140 | 22,720 ± 90 | 26,486-27,278 |
|  | Beta-401729 | 192 | 34,200 ± 270 | 38,535-40,000 |
|  | Beta-405343 | 195 | 34,770 ± 200 | 39,470-40,466 |

*Table S2.4.2.1: radiocarbon dates obtained for the new sites in this study (pMC = percent modern carbon; BP = before radiocarbon present, i.e. 1950 AD).*

Age-depth models were constructed in ‘rbacon’ using the SHCal20 calibration curve and following the approach set out in S2.3 (Blaauw et al., 2020; Blaauw and Christen, 2011; Hogg et al., 2020). A present surface date (see S2.3) was added to constrain the upper parts of the models and prevent overshoot (S3.2).

# S3. Supplementary results

## S3.1. Additional palaeo-vegetation synthesis results

The grids below show each synthesised proxy site’s records. Proxy time series are plotted against ten random iterations of each record’s age-depth model. Rows are in approximate latitudinal order (north to south), and sites are in approximate longitudinal order (west to east) within each row; sites in the same column are generally not found at similar longitudes. Each site is found at the same position in each grid, except for CPCN Pro Mata’s multiple δ^13^C records which occupy several neighbouring panels in the grid.


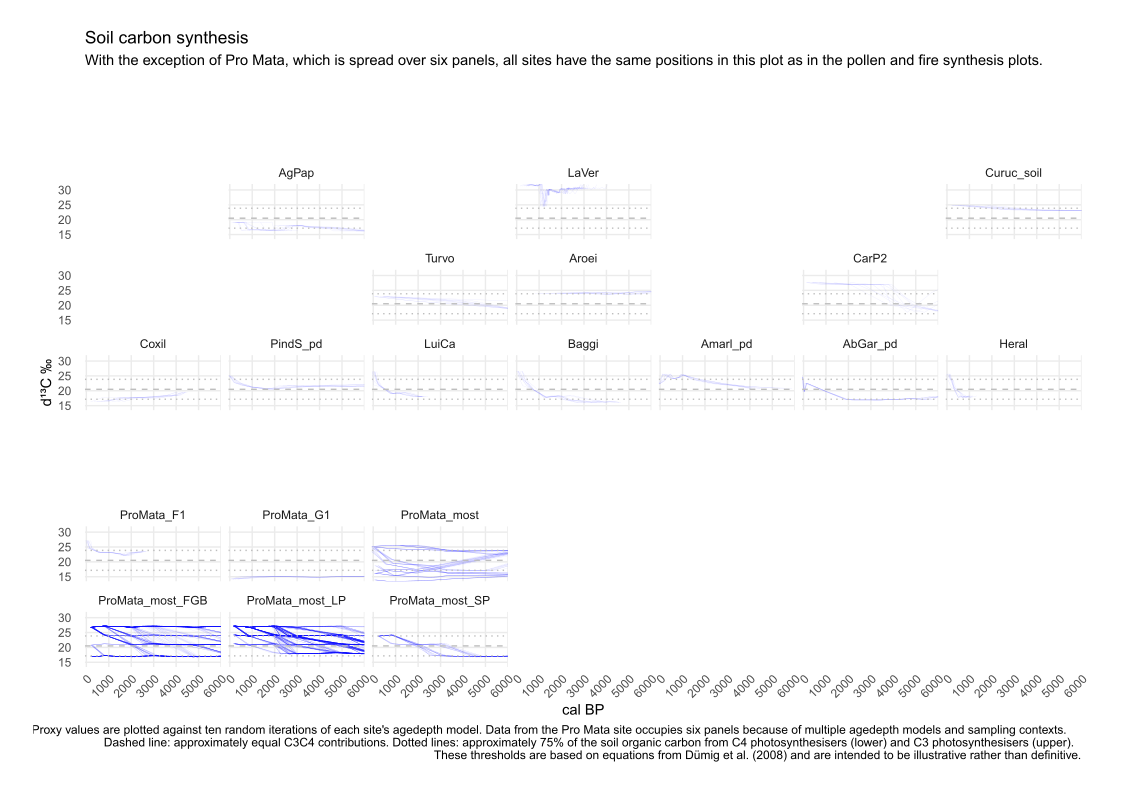


*Fig. S3.1.1. Synthesised δ^13^C records. The dashed line indicates approximately equal C3-C4 contributions, with the dotted lines illustrating values where approximately 75% of the soil organic carbon is derived from Campos species/C4 photosynthesisers (lower) and Araucaria Forest species/C3 photosynthesisers (upper). These values are based on equations from Dümig et al.* (2008) *and are intended to be illustrative rather than definitive.*

*
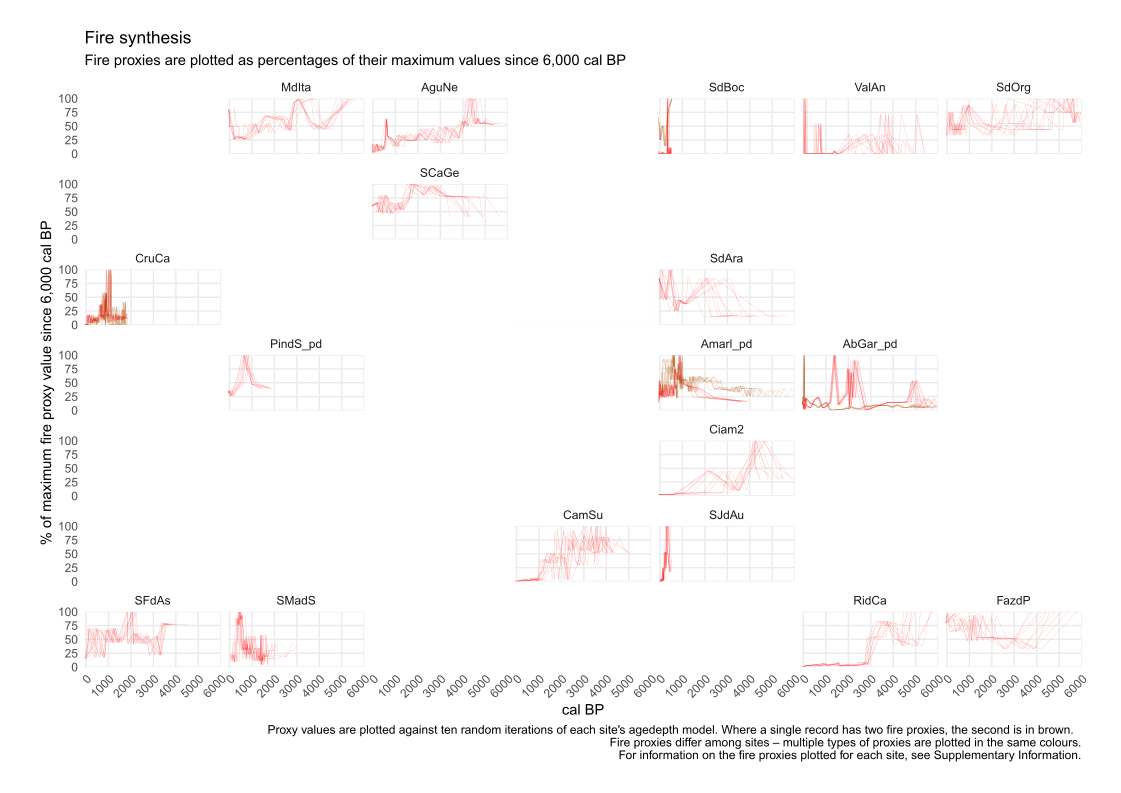
*

*Fig. S3.1.2. Synthesised fire records. Where sites have two records, the second is shown in brown. The sizes of charcoal particles counted are often not specified, and even when specified they vary between studies, so plots in the same colour are not necessarily counting the same thing. Proxy values are shown as percentages of their maximum value since 6,000 cal BP.*

*
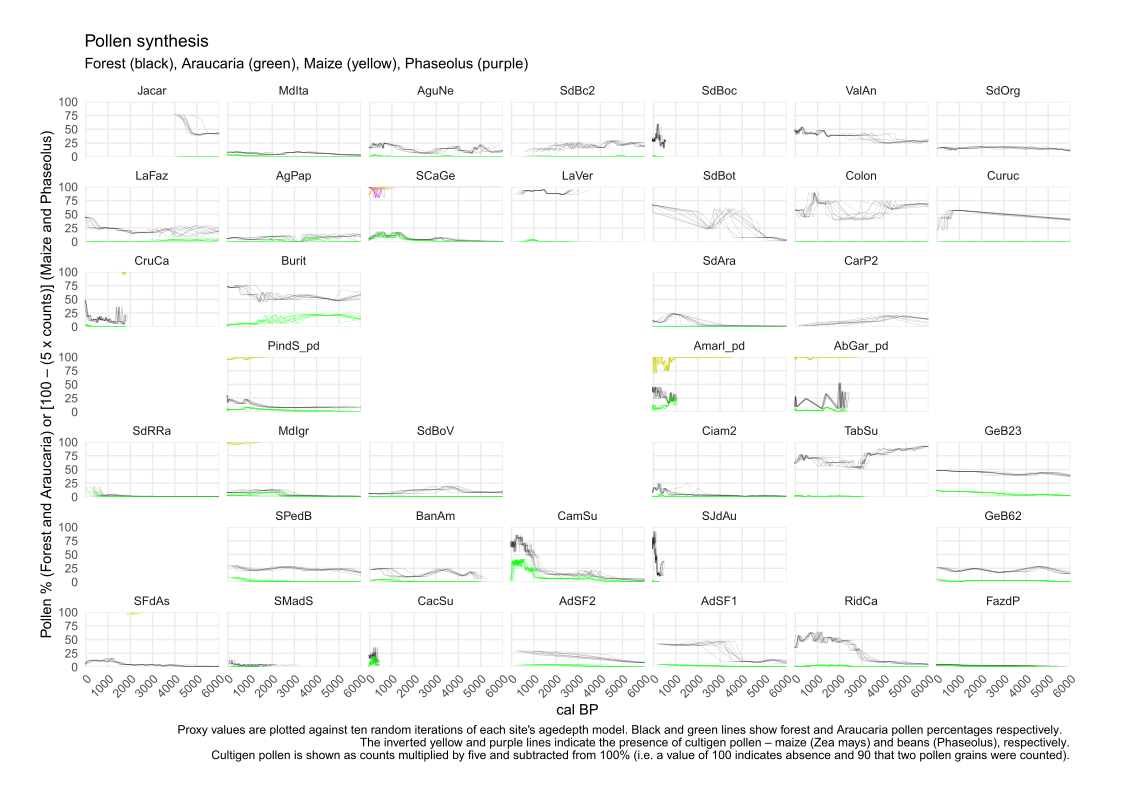
*

*Fig. S3.1.3. Synthesised pollen records. Black and green lines show forest and Araucaria pollen percentages respectively. The inverted yellow and purple lines indicate the presence of cultigen pollen – maize (Zea mays) and beans (Phaseolus), respectively. Cultigen pollen is shown as counts multiplied by five and subtracted from 100% (i.e. a value of 100 indicates absence and 90 that two pollen grains were counted).*

*
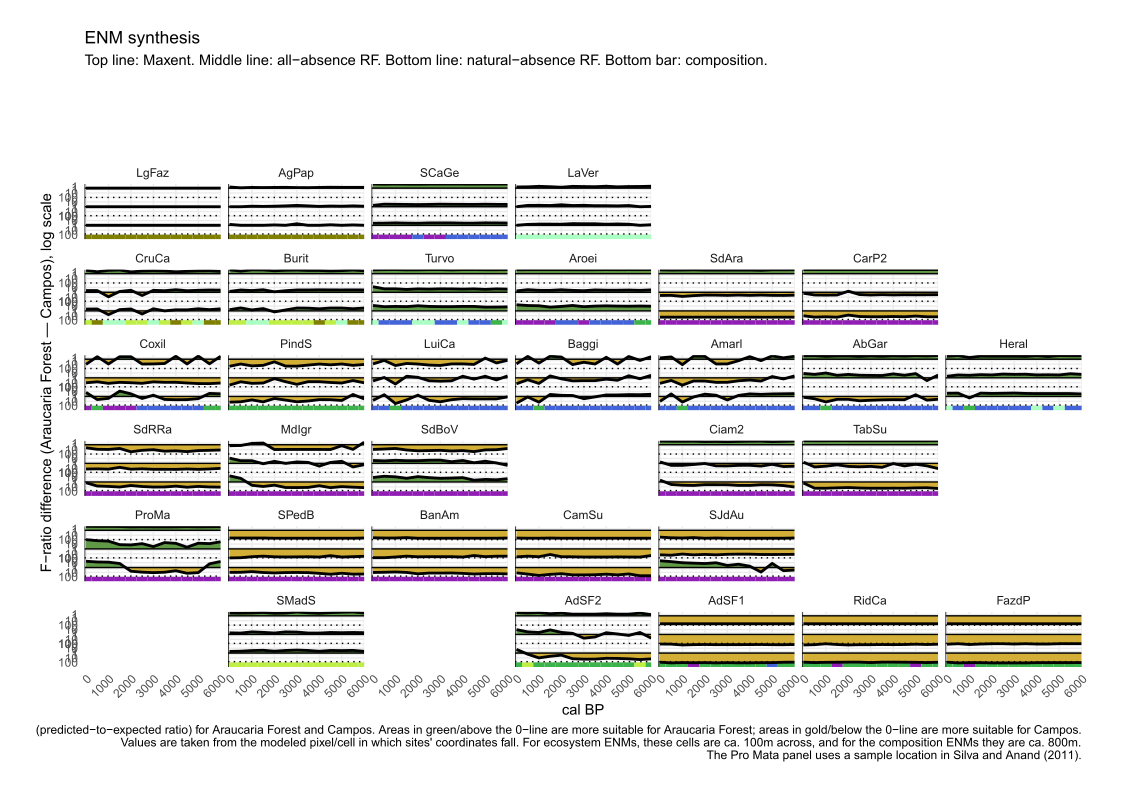
*

*Fig. S3.1.4. ENM results for the pixels containing the proxy sites. Dark green lines represent Araucaria Forest, gold lines represent Campos, and bright green lines represent Araucaria angustifolia (Maxent only). The top row in each panel shows results from Maxent models, the middle row from all-absence RF models, and the bottom row natural-absence RF models. Y-axes are F-ratios (see S2.2.3) on a logarithmic scale; an F-ratio of 1 (dotted lines) can be considered the threshold of marginal habitat suitability. The coloured bars along the bottom of each panel show modelled composition (see S2.2.3 for interpretation).*

There is a striking difference in vegetation-fire relationships between the four sites identified as having high apparent southern Jê influence and the other sites for which pollen and charcoal data are both available (main text fig. 4). Fig. S3.1.5 (below) breaks down the broader trends by site. The ‘high apparent southern Jê influence’ label is applied to subsamples from 1,200 cal BP or later from Abreu e Garcia, Amaral, Pinhal da Serra, and Serra Campos Gerais; earlier subsamples in these records do not have this label. In the Fazenda do Pinto, Serra do Araçatuba and São Francisco de Assis records, the relationships do not clearly follow the wider negative relationship between fire and forest, despite not having significant known southern Jê influence. The Fazenda do Pinto site records no significant forest expansion, with Araucaria Forest pollen not exceeding 5%. Maize pollen was found in the São Francisco de Assis record so its pattern could conceivably be anthropogenic, but it is very unlikely to be connected to the more distant Araucaria Forests or southern Jê. Although *Araucaria* pollen at Serra do Araçatuba never exceeds 1.2%, its positive past relationship between fire and forest cannot be easily explained – it may in fact reflect human influence, but more evidence is needed to clarify this.

*
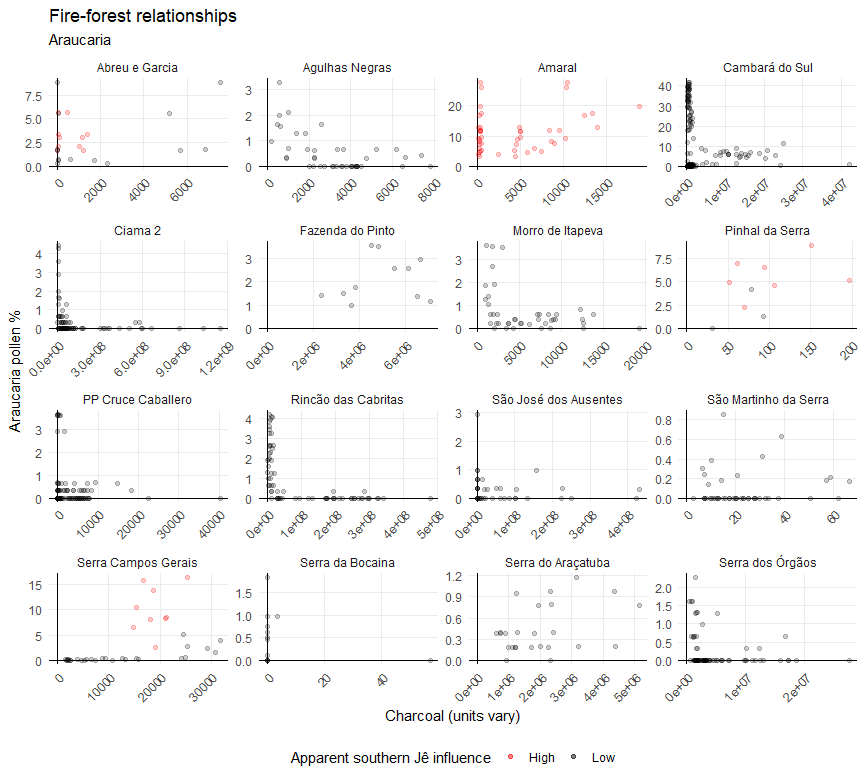

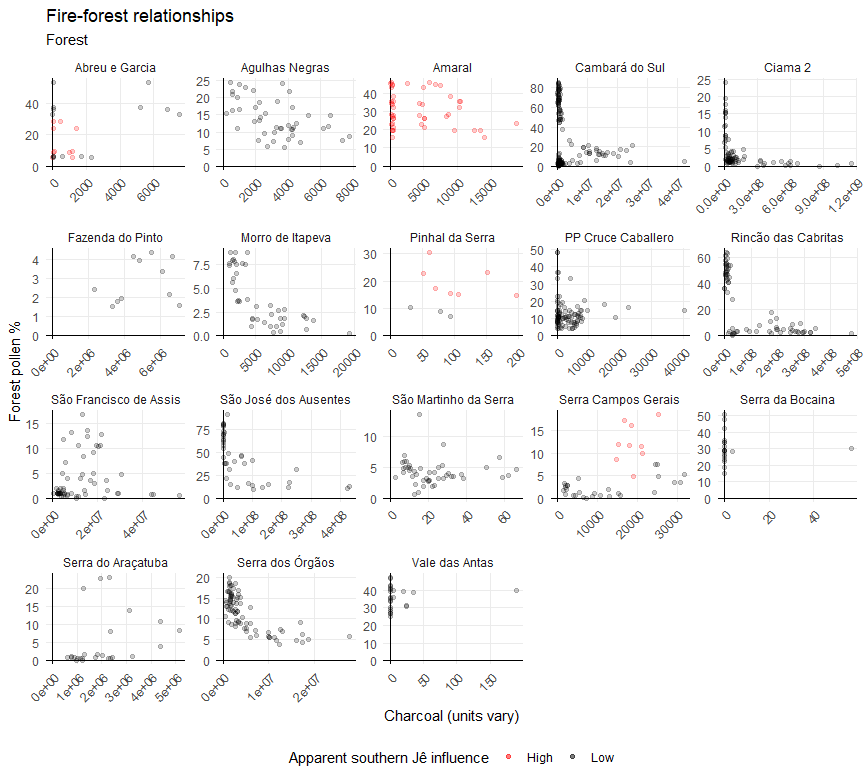
*

*Fig. S3.1.5. Site-level comparisons of Araucaria (top) and forest (bottom) pollen proportions against charcoal concentrations.*

*
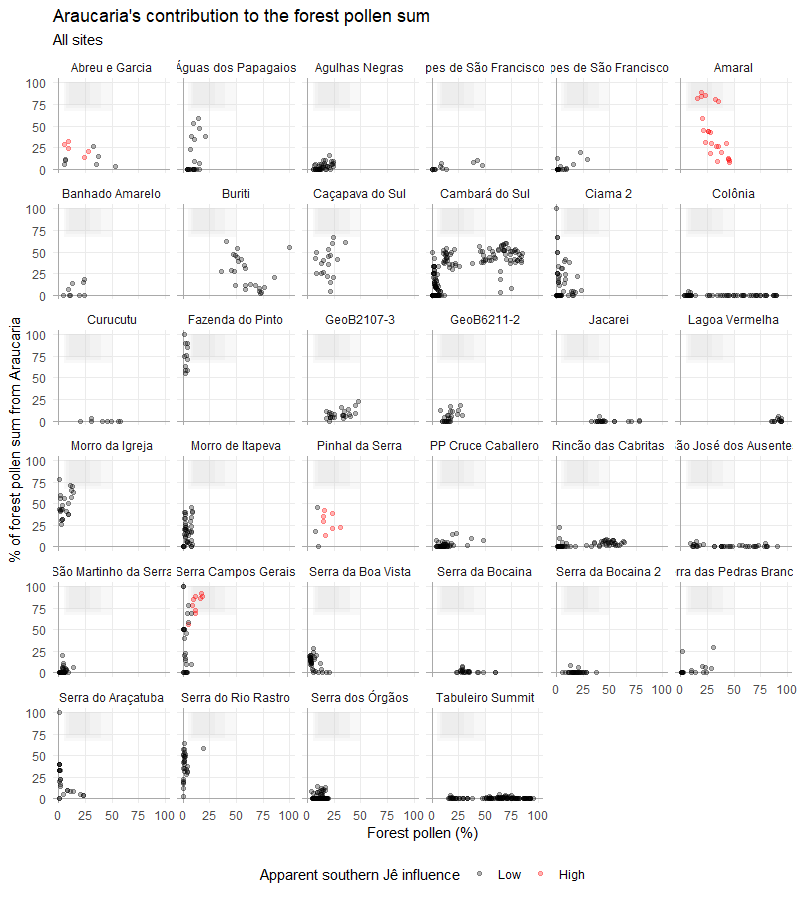
Fig. S3.1.6. Site-level comparisons of (Araucaria) forest pollen and Araucaria’s contribution.*

## S3.2. Additional results from Abreu e Garcia, Amaral, and Pinhal da Serra

### S3.2.1. Abreu e Garcia


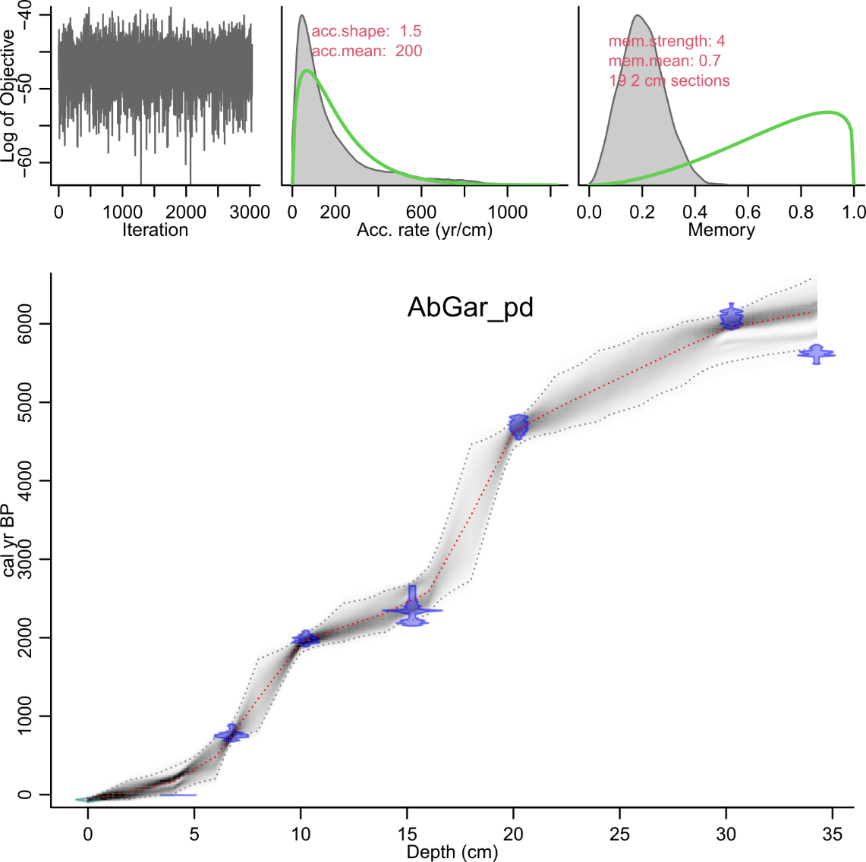


*Fig. S3.2.1.1. Age-depth model for Abreu e Garcia*

*
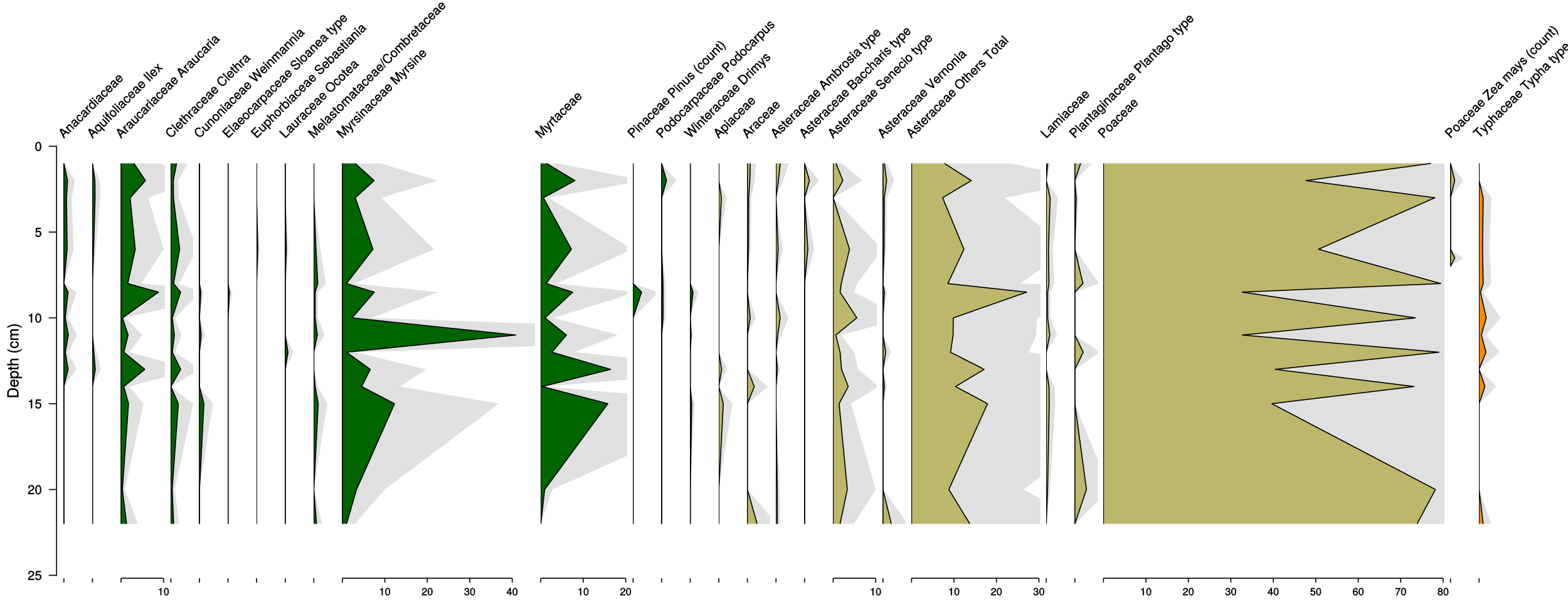

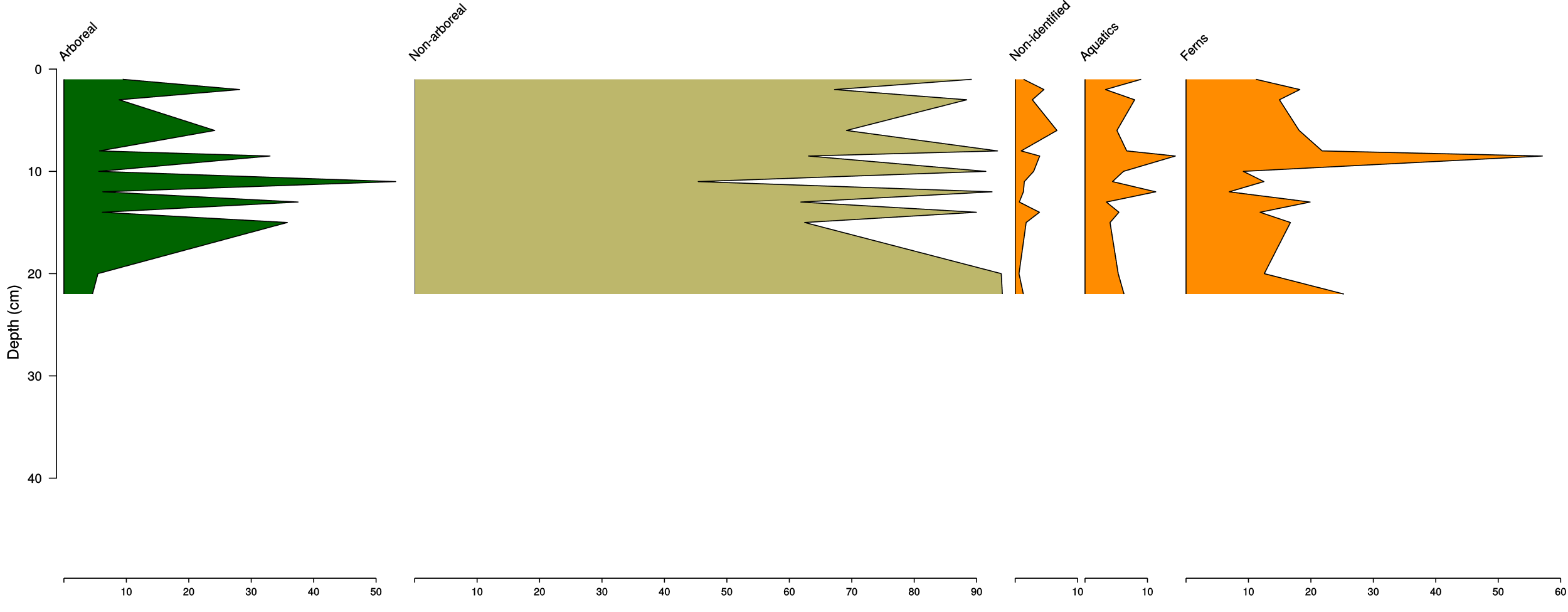

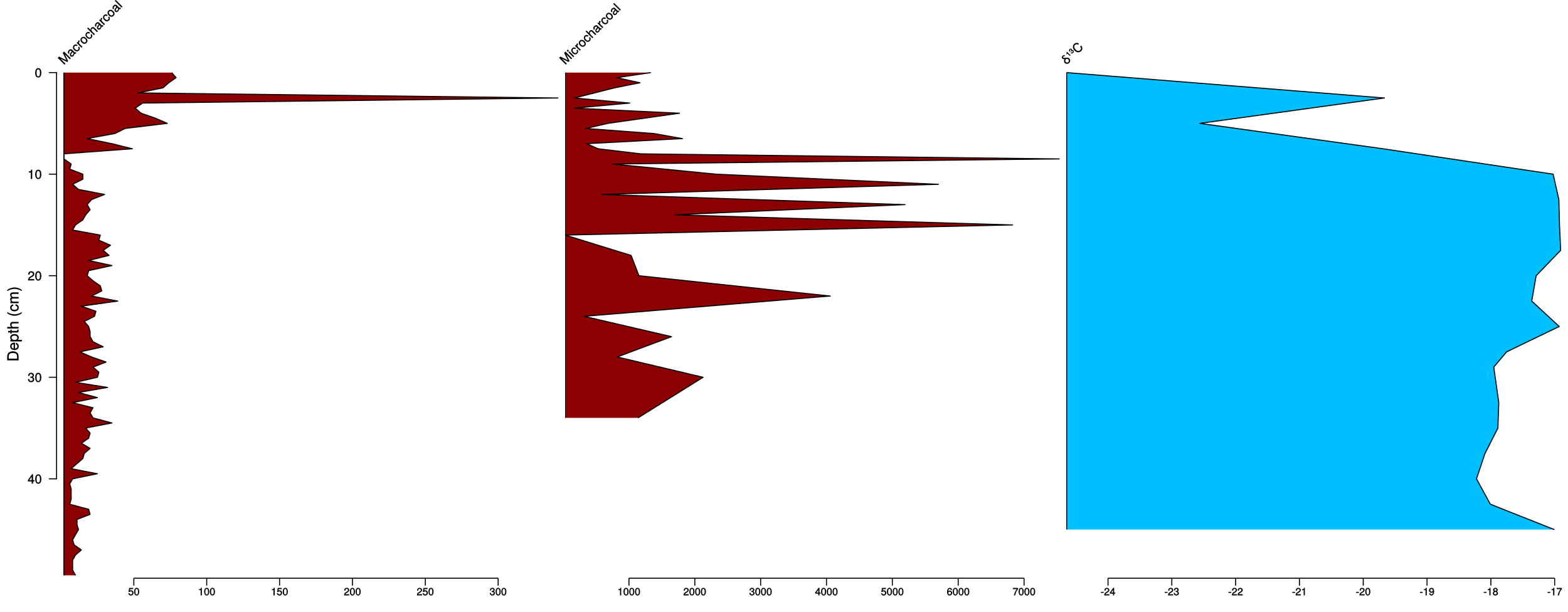
*

*Fig. S3.2.1.2. Expanded proxy diagrams from Abreu e Garcia. All pollen taxa which exceed 1% of the terrestrial sum are shown. Exaggeration is 3x. Aquatics (including Typha) and ferns are plotted as percentages of the terrestrial pollen sum (arboreal + non-arboreal + non-identified). Pine and maize pollen are counts, not percentages.*

### S3.2.2. Amaral


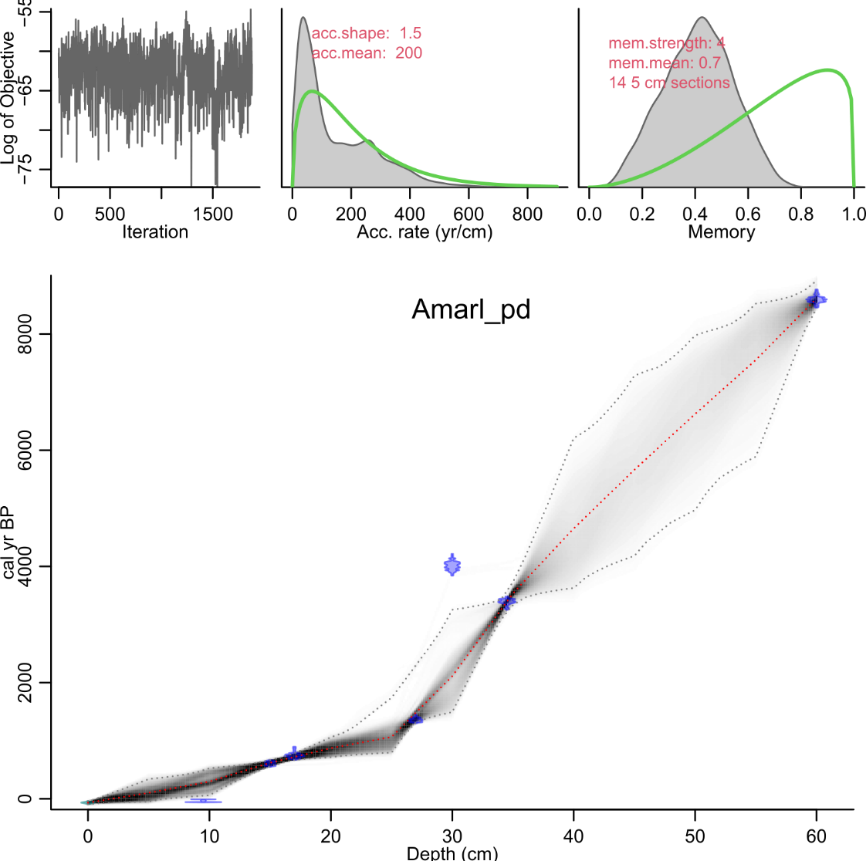


*Fig. S3.2.2.1. Age-depth model for Amaral.*


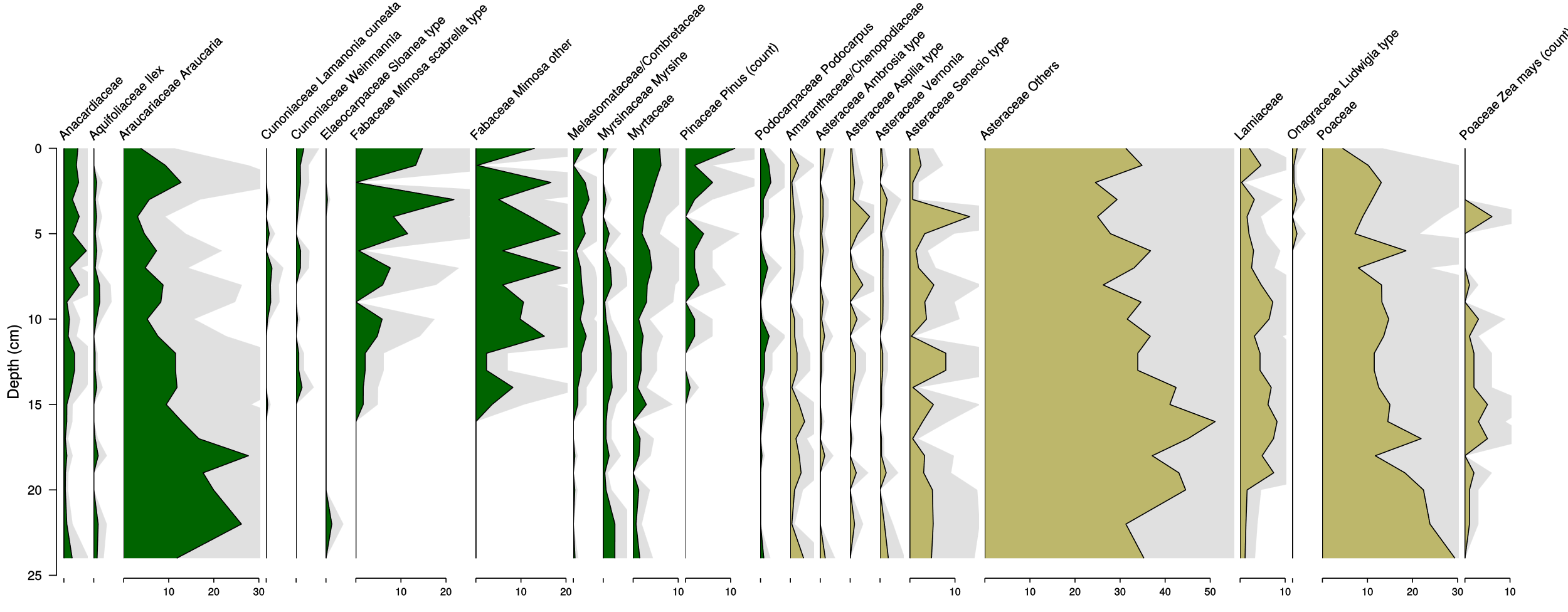


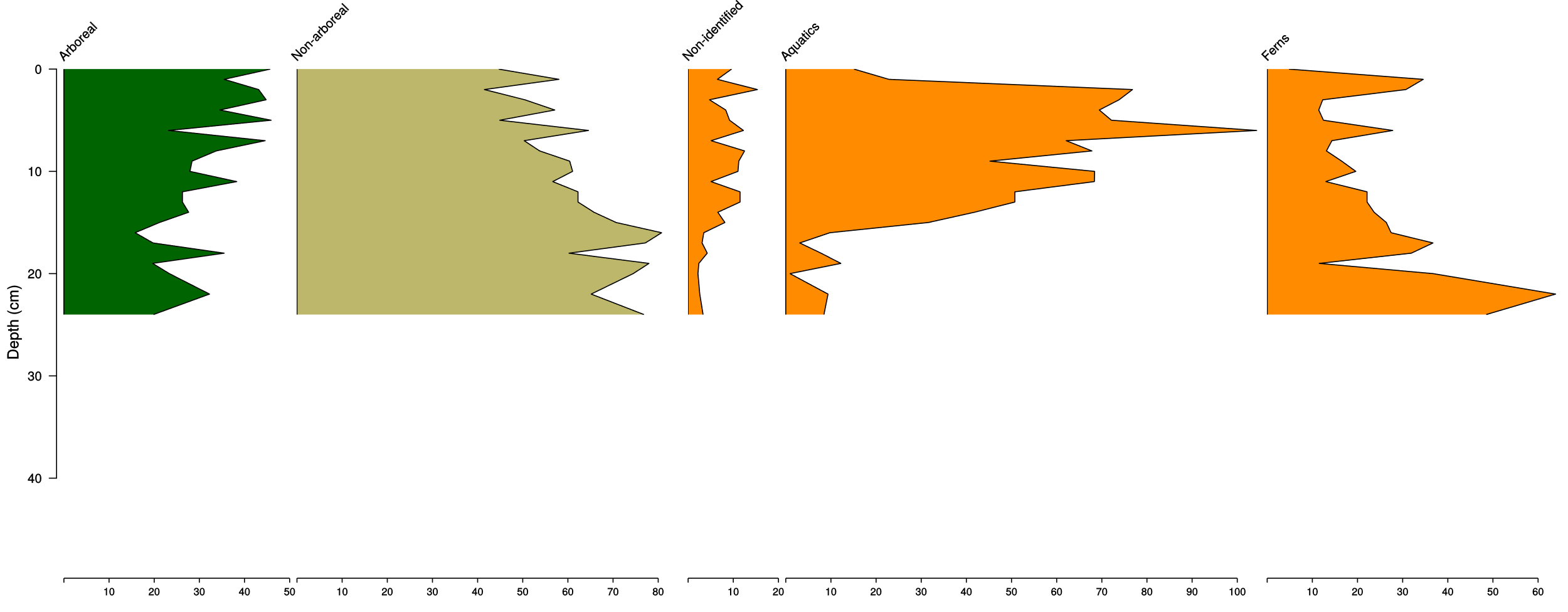

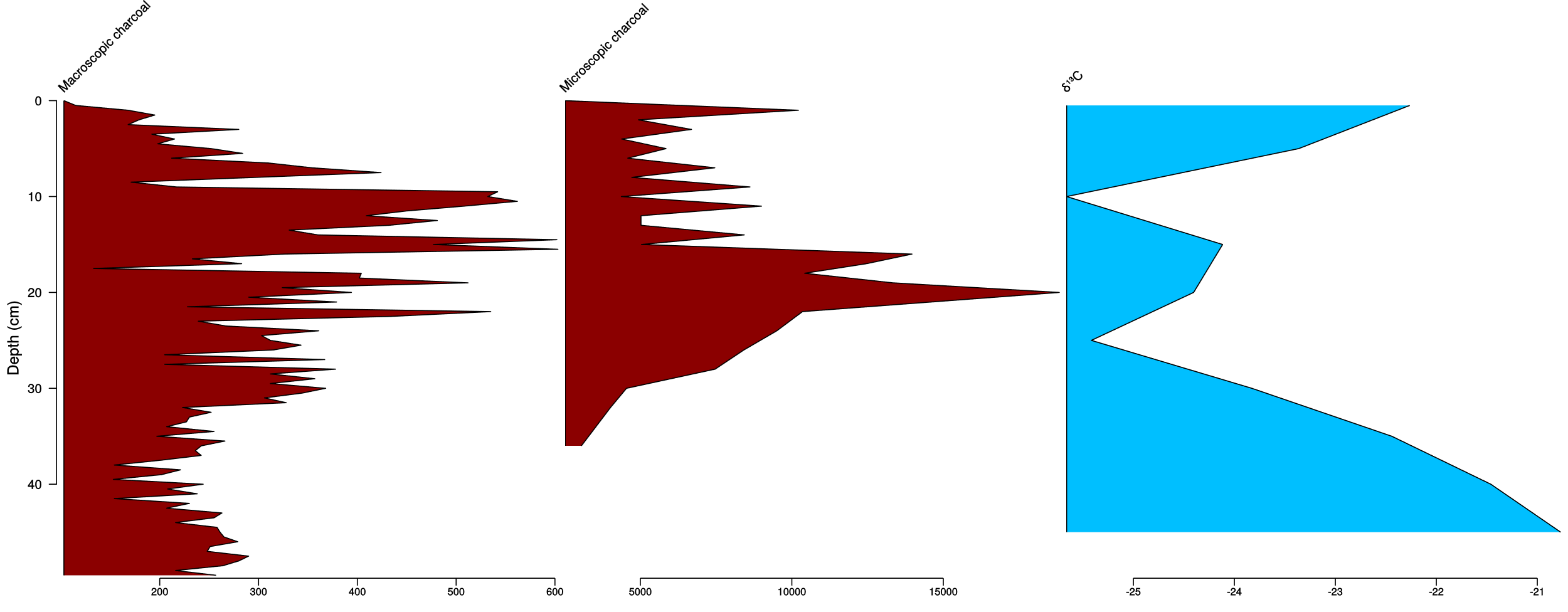


*Fig. S3.2.2.2. Expanded proxy diagrams from Amaral. All pollen taxa which exceed 1% of the terrestrial sum are shown. Exaggeration is 3x. Aquatics and ferns are plotted as percentages of the terrestrial pollen sum (arboreal + non-arboreal + non-identified). Pine and maize pollen are counts, not percentages.*

### S3.2.3. Pinhal da Serra


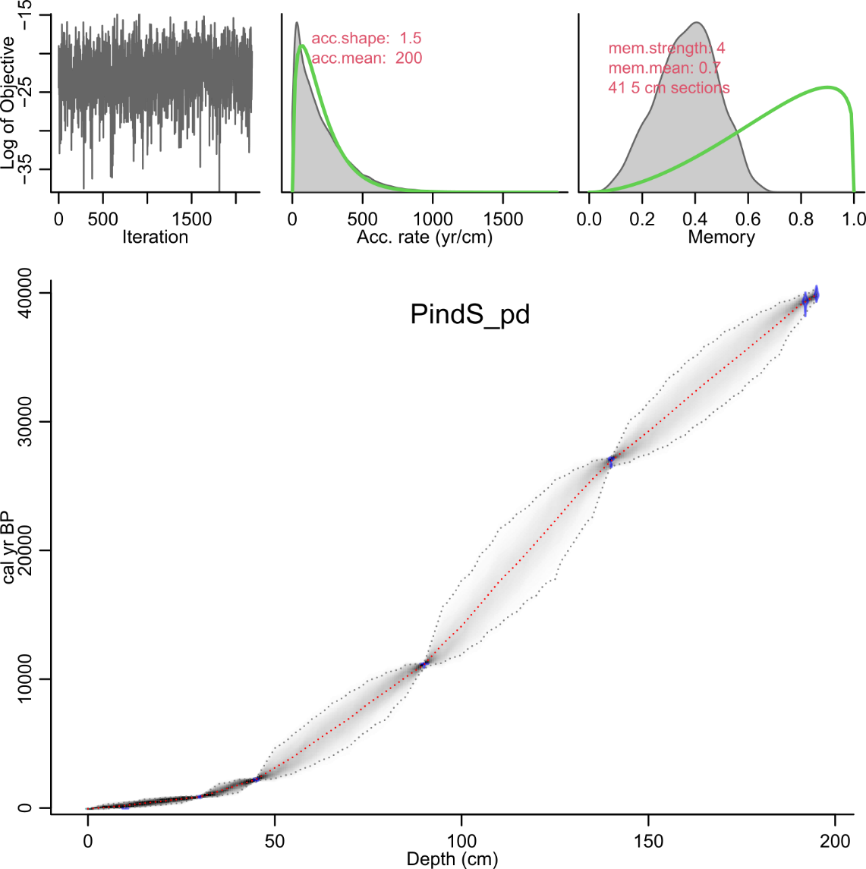

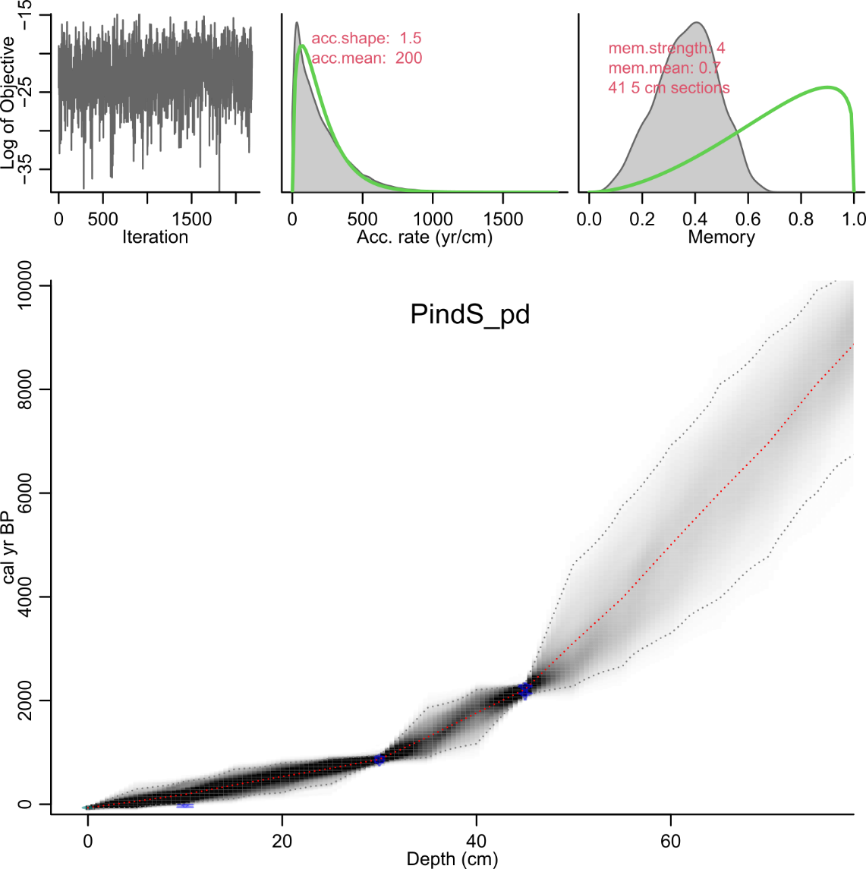


*Fig. S3.2.3.1. Age-depth model for Pinhal da Serra (full depth and top 75 cm only)*

*
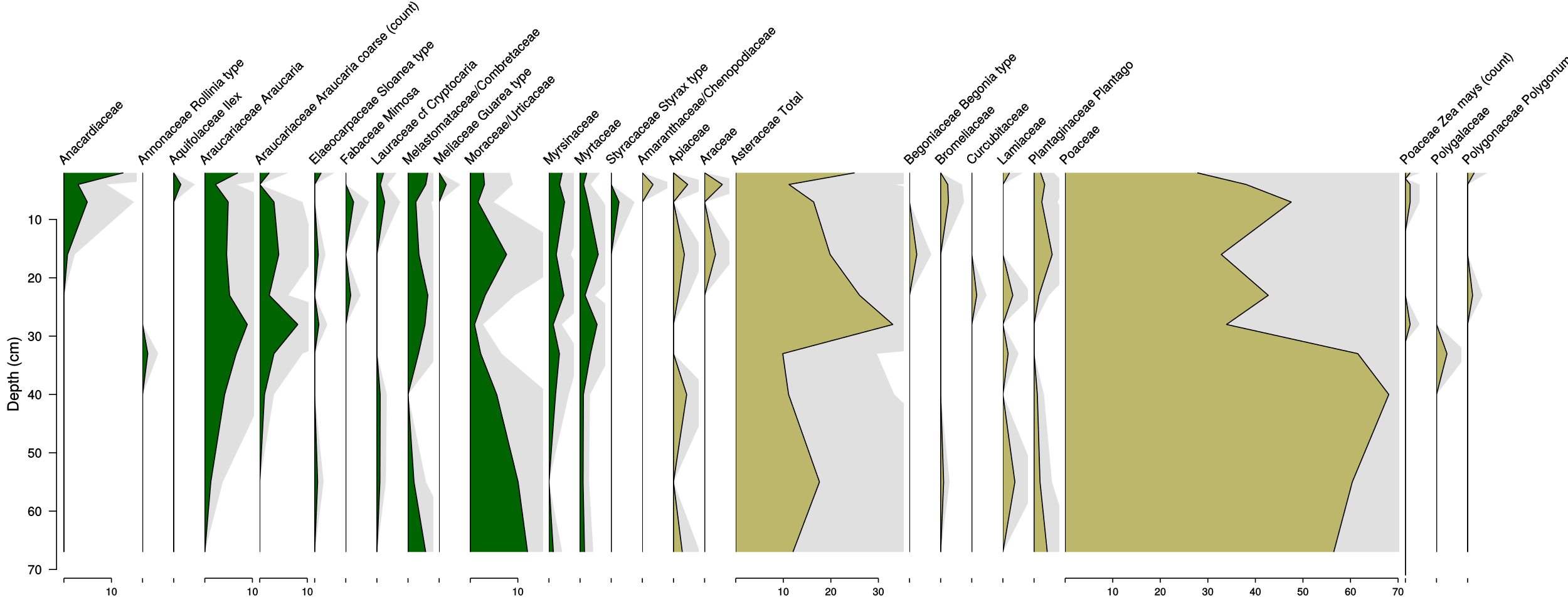
* *
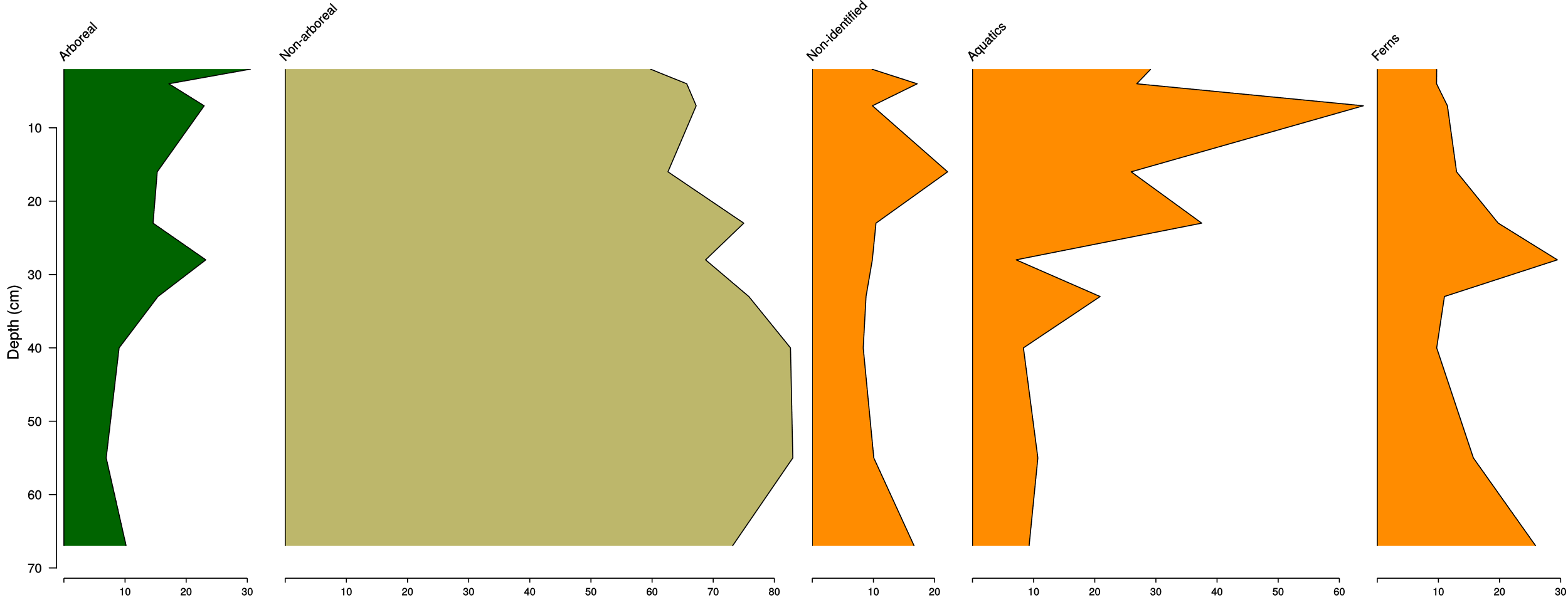
*
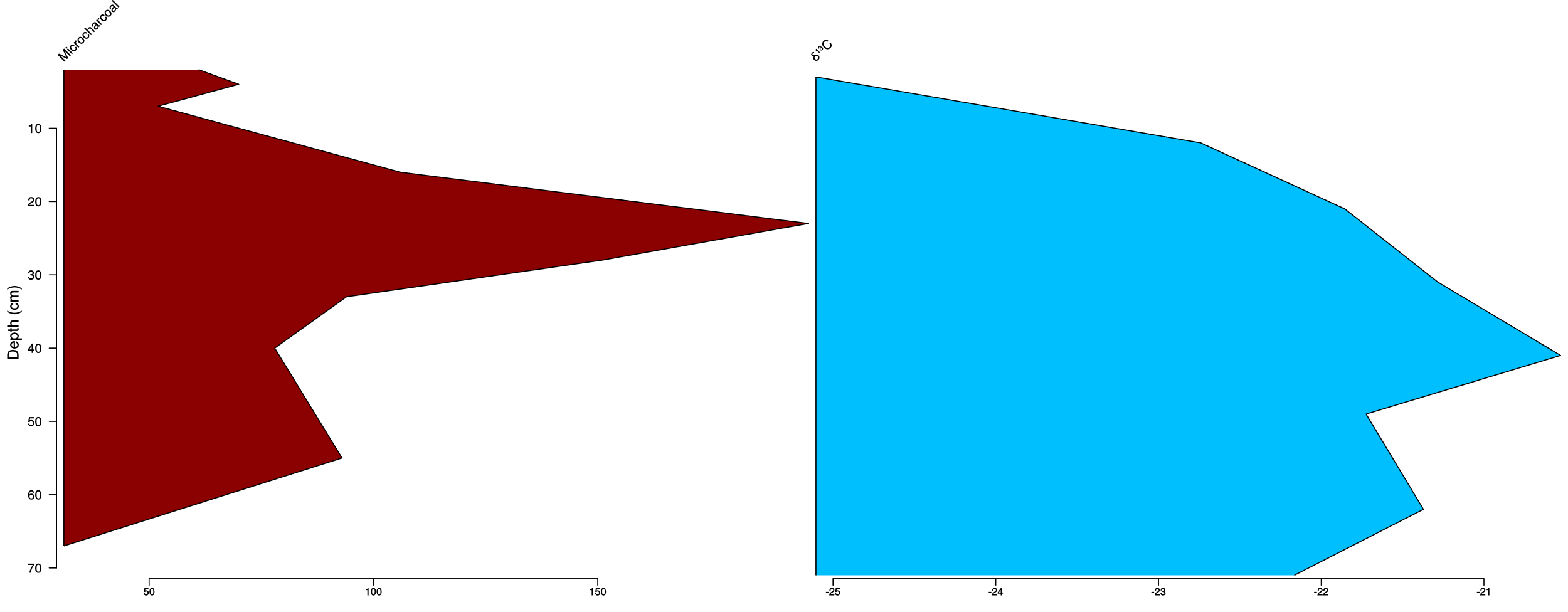
 *Fig. S3.2.3.2. Expanded proxy diagrams from Pinhal da Serra. All pollen taxa which exceed 1% of the terrestrial sum are shown. Exaggeration is 3x. Aquatics and ferns are plotted as percentages of the terrestrial pollen sum (arboreal + non-arboreal + non-identified). Pine and maize pollen are counts, not percentages; a count of Araucaria pollen from the coarse fraction is also shown – this is independent of the percentage in the fine fraction.*

## S3.3. Additional ENM results

| **Ecosystem** | **Model** | **AUC cross-validation** | **AUC set-aside evaluation** | **TSS cross-validation** | **TSS set-aside evaluation** | **Boyce Index (Spearman correlation)** |
| --- | --- | --- | --- | --- | --- | --- |
| Araucaria Forest | Maxent | 0.910 | 0.897 | - | - | 0.969 |
|  | RF all absences | 0.947 | 0.946 | 0.818 | 0.814 | 0.994 |
|  | RF natural absences | 0.954 | 0.977 | 0.845 | 0.881 | 0.999 |
| Campos | Maxent | 0.988 | 0.987 | - | - | 0.986 |
|  | RF all absences | 0.993 | 0.978 | 0.970 | 0.924 | 0.975 |
|  | RF natural absences | 0.997 | 0.990 | 0.986 | 0.903 | 0.982 |

*Table S3.3.1: evaluation metrics for ecosystem-level ENMs.*


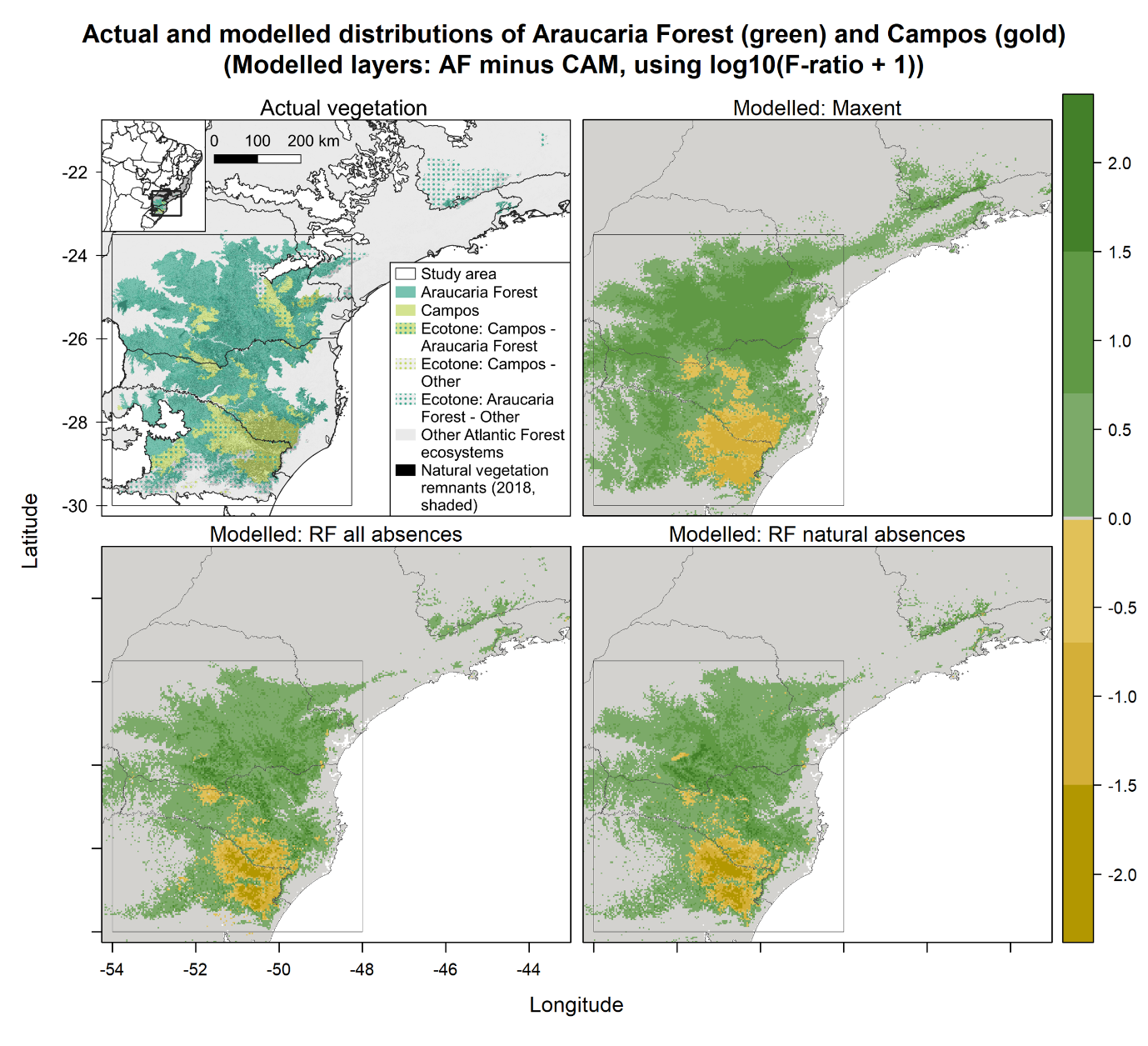


*Fig. S3.3.1. Actual* (IBGE - Instituto Brasileiro de Geografia e Estatística, 2019; Souza et al., 2020) *and modelled present-day distributions of Araucaria Forest and Campos. Green areas are more likely to be Araucaria Forest, gold areas are more likely to be Campos, and grey areas are either finely balanced between the two or both are absent (further details can be found in S2.2.3).*


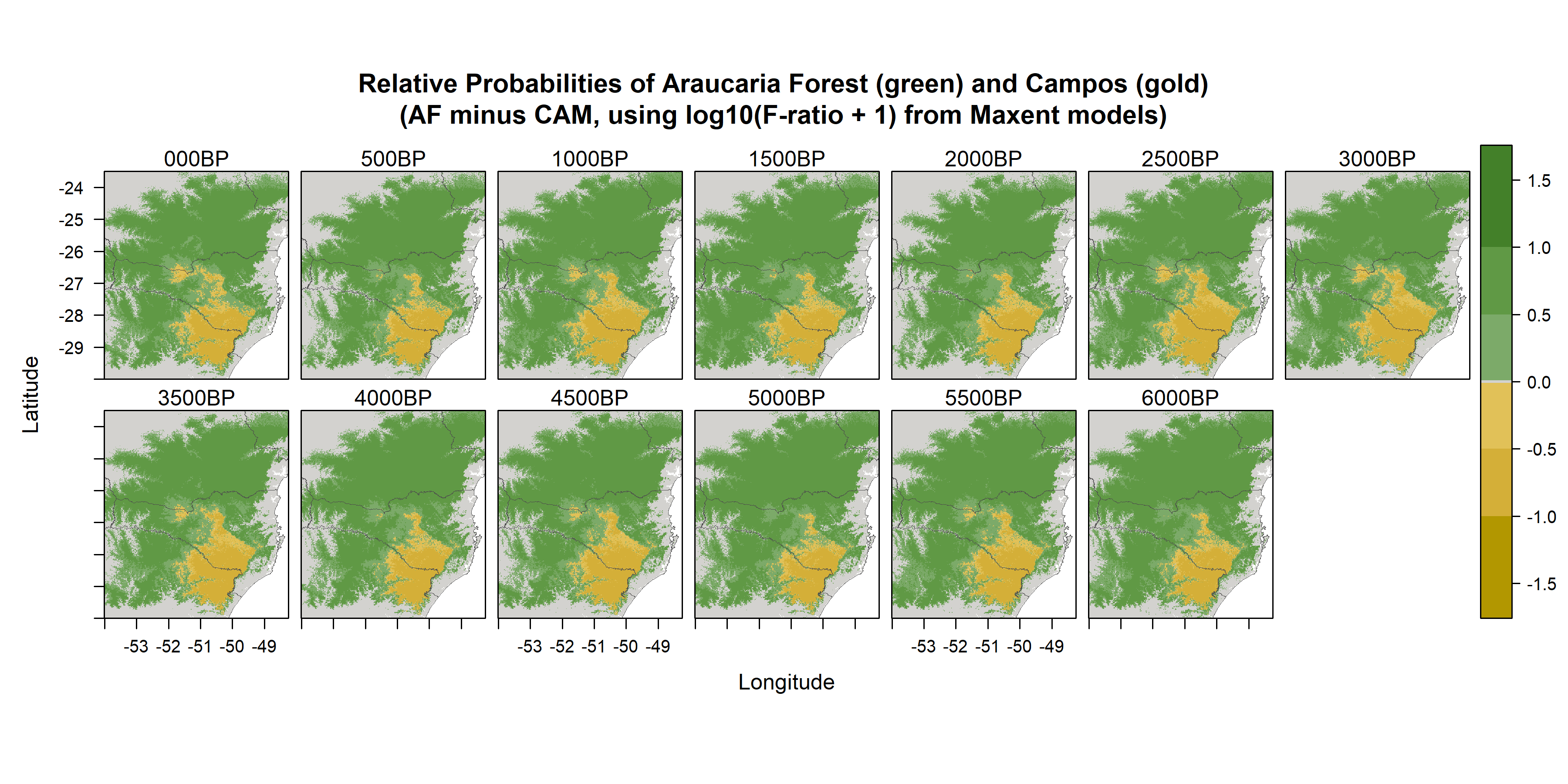

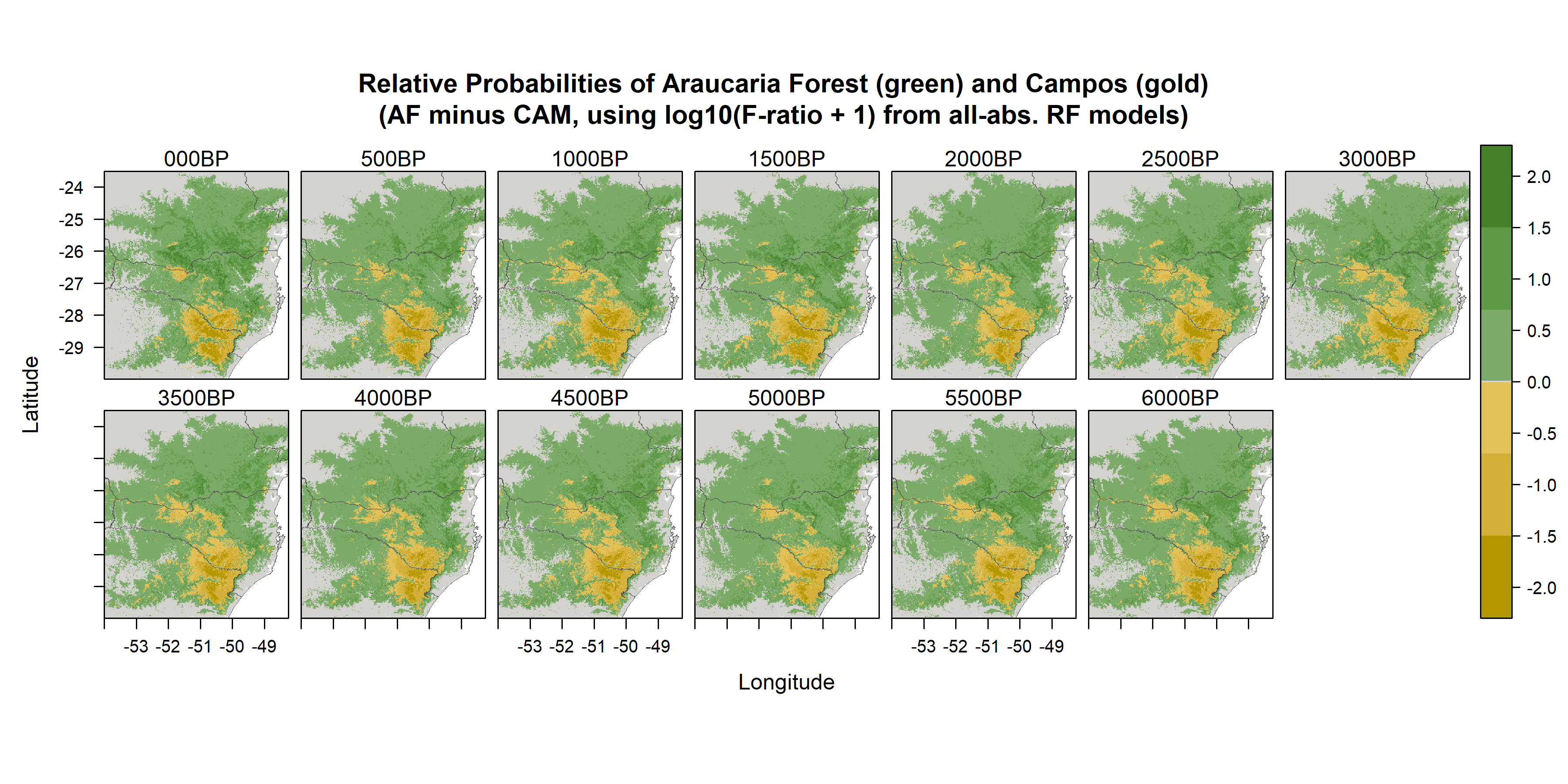

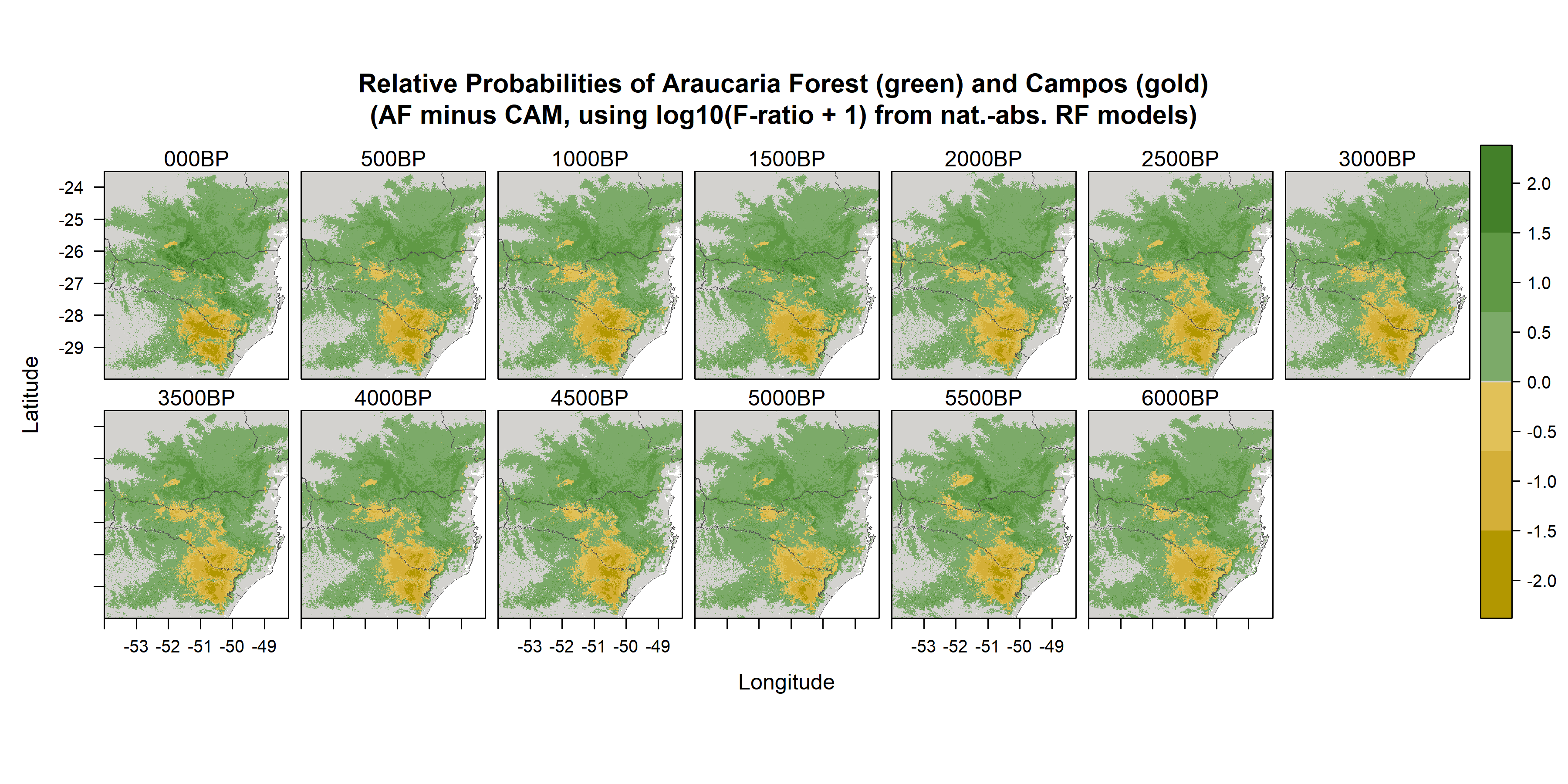


*Fig. S3.3.2. Ecosystem-level ENM results showing the predicted relative probability of Araucaria Forest and Campos in space and time (top-bottom: Maxent, all-absence RF, natural-absence RF).*

*
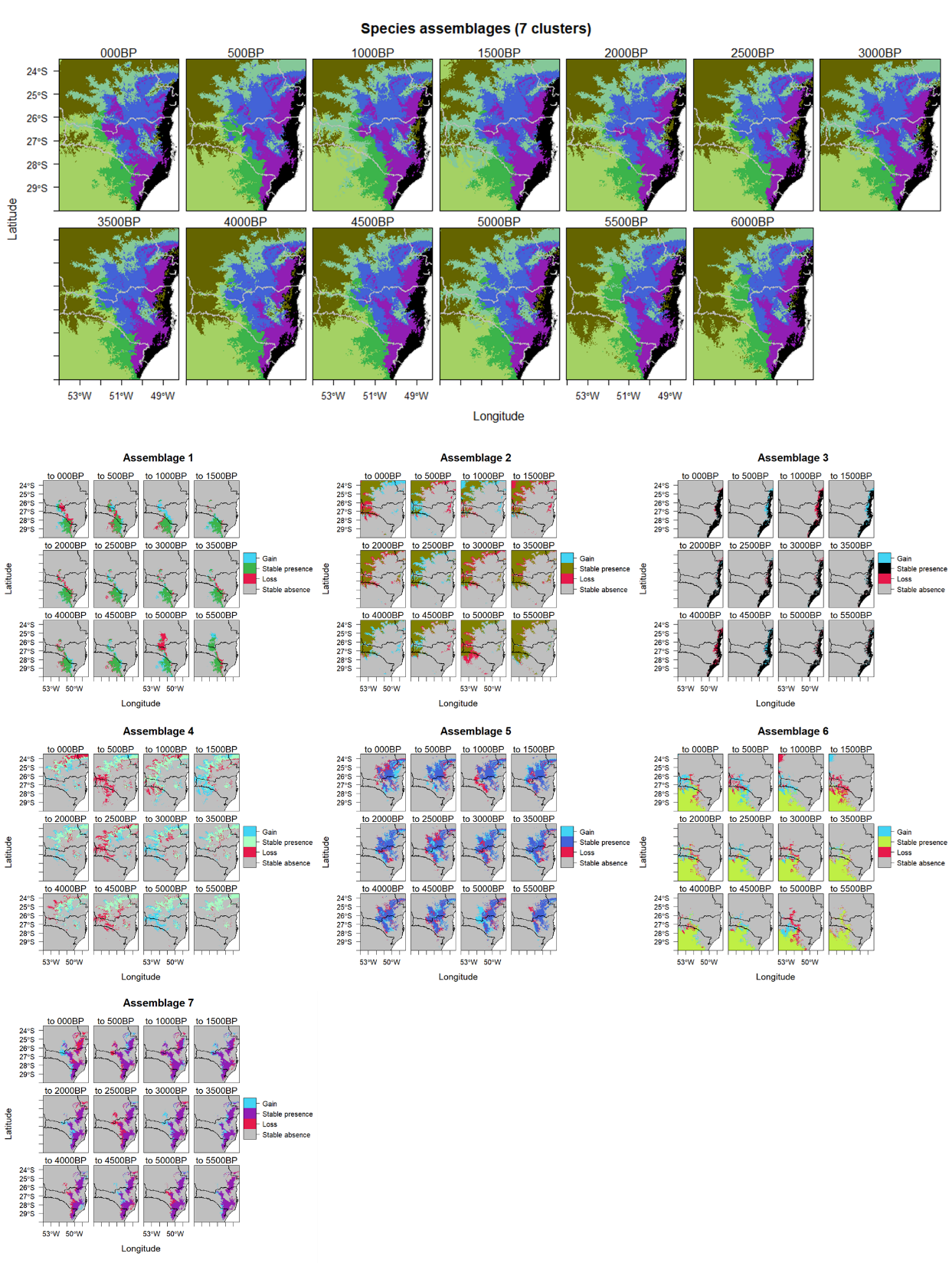
*

*Fig. S3.3.3. Predicted compositional change through space and time. For interpretation of the colours, see fig. S2.2.3.1. In the lower half of the plot, each assemblage’s colour represents stable presence and grey means stable absence, while red represents area lost from the previous time point and light blue represents gained area.*

# S4. Supplementary discussion

## S4.1. Identifying evidence of pre-colonial Indigenous impacts on Araucaria Forest

The three new palaeoecological sites studied in this paper – Abreu e Garcia, Amaral and Pinhal da Serra – are all within 1km of known archaeological sites in an area with significant archaeological evidence of pre-colonial southern Jê occupation (see S2.4 and main text fig. 1d). They are therefore well placed to identify signals of land use in more intensely occupied parts of the landscape. In all three records we find increases in micro- and macrocharcoal with maize pollen around the time radiocarbon dates from nearby archaeological sites have shown southern Jê occupation. In Amaral and Pinhal da Serra, cultivation and increases in fire co-occur with relatively high percentages of Araucaria Forest pollen, of which *A. angustifolia* makes up a relatively high proportion (fig. S3.1.5-6). Together, these are strong indicators of human influence on Araucaria Forest.

Unexpectedly, we also identified the same pattern in the Serra Campos Gerais record (Behling, 1997b), 350 km away from our new study sites. Before about 700 cal BP, the site follows expected natural dynamics, with declining charcoal occurring with increases in Araucaria Forest and *A. angustifolia* pollen. After about 700 cal BP, however, the trend in charred particles reverses course and increases, cultigen pollen (maize and *Phaseolus*) appears, and Araucaria Forest (most of it *A. angustifolia*) pollen increases. Serra Campos Gerais is not near to any known southern Jê archaeological sites but the combination of these signals does suggest past human land use.

Some other sites (fig. S3.1.6) have sustained periods where *A. angustifolia’s* pollen percentages are high in absolute terms (Buriti (Bertoldo et al., 2014) before about 2,000 cal BP) or relative to the forest pollen sum (Águas dos Papagaios (Ladchuk et al., 2016) 4,000-2,000 cal BP), but the combination of both seems to be predominantly found in human-shaped sites. It is important to note, though, that the attribution of this pattern to human influence should not be automatic. These areas with distinctive Araucaria Forest pollen signatures are associated with more evidence of past human occupation as well as other, potentially confounding, explanatory variables – they are generally further inland at lower elevations (with attendant climatic differences), further from Campos and closer to contiguous Araucaria Forest, any of which could naturally result in floristic differences compared to the more Campos-dominated fossil pollen sites at higher elevations closer to the escarpment. Therefore, although high *A. angustifolia* pollen percentages do often appear to be associated with more intense pre-colonial Indigenous occupation, the link is not necessarily causal.

Cambará do Sul (Behling et al., 2004) has both these features – for almost the entire period since 1,000 cal BP, *A. angustifolia* pollen was 20-40% of the terrestrial sum and 40-60% of the Araucaria Forest total – but the absence of fire, nearby archaeology or evidence of cultivation, and the record’s congruence with natural drivers (see main text results section), suggests these are natural in this instance. Caçapava do Sul (Behling et al., 2016) experiences a sudden doubling or tripling of its forest pollen percentage (7-13% to 20-36%) around 250 or 200 cal BP, driven largely by abrupt increases in *A. angustifolia* pollen (rising to make up 40-65% of the forest pollen sum). These changes are unlikely to be related to the southern Jê (the site is in the lowlands 70 km south of the plateau), and their connection to human activity is not clear: the site’s fire dynamics are not recorded, and while the surrounding area had already been occupied by Guarani groups for a significant time (Bonomo et al., 2015; Iriarte et al., 2017), the timing of the changes also places them close to the onset of the colonial period. The only other similar site is Morro da Igreja (Behling, 1995), where, for a prolonged period, *A. angustifolia* pollen made up 3-9% of all terrestrial pollen and 38-71% of Araucaria Forest (though, as with Serra Campos Gerais, some other trees are assigned to different pollen groups), including at 8cm depth where a grain of cultivated *Zea mays* pollen was found. However, the record’s age uncertainties are so great in this period (1,000-2,900 years between maximum and minimum estimates) that it is not possible to ascribe this pattern to Indigenous land use.

## S4.2. Potential Araucaria Forest transformations from low-intensity pre-colonial land use

Our study provides clear evidence of southern Jê actions transforming Araucaria Forest in intensely occupied areas close to settlements and cultivated areas, approximating the ‘home’ and ‘clean space’ domains in contemporary southern Jê ethnolandscapes (Corteletti et al., 2023; Moura, 2021; Réus Gonçalves Da Rosa, 2005). However, the design of our study does not allow us to resolve the question of how human actions affected less intensively occupied or used landscapes, such as the ‘virgin forest’ (‘floresta virgem’) ethno-landscape domain. (N.B., this term is used by contemporary southern Jê groups but is problematic in wider discourse; see e.g. Fletcher et al., 2021.) There are exceedingly few Araucaria Forest palaeoecological proxy sites further inland than those we introduce in this paper, so insights into potential management in more contiguous forest areas are limited; they may have differed substantially from the better studied higher-elevation areas around Araucaria Forest-Campos ecotones and mosaics. In any case, it can be inherently difficult to determine the impacts of low-intensity human land use on forest composition.

Nonetheless, there are good reasons to expect that the southern Jê influenced areas of Araucaria Forest they did not use or occupy intensively (dos Reis et al., 2014; Souza, 2021). Various sources of evidence show that southern Jê groups travelled significant distances to collect, transport, trade and store pinhão (*A. angustifolia* seeds) (e.g. Corteletti et al., 2015; Cruz, 2014; Fernandes and Piovezana, 2015; Gomes, 2018; Henry, 1964; Moura, 2021; Scheel-Ybert and Boyadjian, 2020; Wesolowski et al., 2010). Such actions could easily start, spread or enrich Araucaria populations (for a detailed discussion of potential mechanisms, see Levis et al., 2018). In this, *A*. *angustifolia* has a clear analogue in Amazonia’s Brazil Nut tree, *Bertholletia excelsa* (an analogy also noted by Lauterjung et al., 2018).

Both *A. angustifolia* and *B. excelsa* are floristically dominant, tall, canopy-emergent trees with significant cultural importance to Indigenous groups, who do and/or did travel long distances to collect the trees’ abundant, large and nutritious seeds, which are otherwise largely dispersed by gravity and scatter-hoarding rodents (and, to an extent for *A. angustifolia,* birds) (dos Reis et al., 2014; Ribeiro et al., 2014; Shepard and Ramirez, 2011; Souza, 2021; Tella et al., 2016; Thomas et al., 2015). Low-intensity harvesting by contemporary Kayapó Brazil Nut collectors has shaped *B. excelsa* distributions, with deliberately planted and unintentionally dropped seeds increasing recruitment within *B. excelsa* groves and along paths that lead back to Kayapó villages (Ribeiro et al., 2014). At a regional-scale, *B. excelsa*’s distribution appears to have been significantly shaped by pre-colonial Indigenous people (Shepard and Ramirez, 2011; Thomas et al., 2015). The similarities between the two species’ socio-ecological traits strongly suggests the southern Jê could have similarly influenced *A. angustifolia* in the pre-colonial era. For example, although increases in C3 contributions to soil carbon in the δ^13^C record at Baggio (Robinson et al., 2018) are compatible with climatic drivers (see main text and S3), these increases in forest in the last 500 years could still be connected to the establishment of the Baggio 1 pit-house village (de Souza, 2018; Robinson et al., 2018). Araucaria pollen declined in the nearby Amaral site after the settlement was founded so people probably had to travel further afield to collect pinhão; if the Baggio valley held riverine forests (plausible but not made explicit in Robinson et al., 2018), decades of travel to and from the village and this area could have led to rapidly expanded or newly established Araucaria Forest areas.

Finally, some contemporary ethnoecological research and Indigenous knowledge suggests the southern Jê may have deliberately manipulated *A. angustifolia* populations in areas with less intense occupation (as well as, e.g., planting Araucaria trees in villages; Gomes, 2018). Gomes (2018, p. 237) reports two isolated adult Araucarias near to an old road and gardens, which a Xokleng-Laklãnõ elder indicated may have been connected with past pinhão-collecting expeditions. In a media interview (<https://web.archive.org/web/20210814124219/https://www.plural.jor.br/noticias/vizinhanca/povos-indigenas-lutam-pela-permanencia-na-floresta-estadual-metropolitana-em-piraquara/>), Kretã Kaingang, a Kaingang political leader, stated that ‘The araucaria is a territorial symbol because, in Brazil, they were planted by our Kaingang ancestors.‘ (Original: ‘A araucária é um símbolo territorial porque, no Brasil, elas foram plantadas pelos nossos ancestrais Kaingang.’) The elaboration of such understandings of past human-environment relationships could serve to advance our knowledge of how southern Jê (and Guarani) people shaped Araucaria Forests and Campos before European colonisation. External interpretations of southern Jê ethnoecology are useful – as in this study and others (e.g. de Souza et al., 2016a; Robinson et al., 2017), they provide a valuable lens for interpreting archaeological and palaeoecological evidence – but an additional promising avenue for future research would be the collaborative combination of ‘classically scientific’ and Indigenous ecological knowledge systems to improve our understanding of past vegetation dynamics in southern Brazil (cf. Richer and Gearey, 2017; Wilson and Marchant, 2025; Zurita-Benavides et al., 2016), as with Machado et al. (2020) and Xokleng-Laklãnõ archaeological artifacts.

# References

Aiello-Lammens, M.E., Boria, R.A., Radosavljevic, A., Vilela, B., Anderson, R.P., 2015. spThin: an R package for spatial thinning of species occurrence records for use in ecological niche models. Ecography 38, 541–545. https://doi.org/10.1111/ECOG.01132

Andrade, B.O., Bonilha, C.L., Overbeck, G.E., Vélez‐Martin, E., Rolim, R.G., Bordignon, S.A.L., Schneider, A.A., Vogel Ely, C., Lucas, D.B., Garcia, É.N., dos Santos, E.D., Torchelsen, F.P., Vieira, M.S., Silva Filho, P.J.S., Ferreira, P.M.A., Trevisan, R., Hollas, R., Campestrini, S., Pillar, V.D., Boldrini, I.I., 2019. Classification of South Brazilian grasslands: Implications for conservation. Applied Vegetation Science 22, 168–184. https://doi.org/10.1111/avsc.12413

Ashcroft, M.B., Chisholm, L.A., French, K.O., 2008. The effect of exposure on landscape scale soil surface temperatures and species distribution models. Landscape Ecology 23, 211–225. https://doi.org/10.1007/s10980-007-9181-8

Aubreville, A., 1948. Quelques problèmes forestiers du Brésil. La forêt de pins de Parana, les plantations d’eucalyptus. Bois et Forêts des Tropiques 6, 102–117.

Bauermann, S.G., Macedo, R.B., Behling, H., Pillar, V. de P., Neves, P.C.P. das, 2008. Dinâmicas vegetacionais, climáticas e do fogo com base em palinologia e análise multivariada no Quaternário tardio do sul do Brasil. Revista Brasileira de Paleontologia 11, 87–96.

Behling, H., 2006. Late Quaternary vegetation, fire and climate dynamics of Serra do Araçatuba in the Atlantic coastal mountains of Paraná State, southern Brazil. Vegetation History and Archaeobotany 16, 77–85. https://doi.org/10.1007/s00334-006-0078-2

Behling, H., 1997a. Late Quaternary vegetation, climate and fire history from the tropical mountain region of Morro de Itapeva, SE Brazil. Palaeogeography, Palaeoclimatology, Palaeoecology 129, 407–422. https://doi.org/10.1016/S0031-0182(97)88177-1

Behling, H., 1997b. Late Quaternary vegetation, climate and fire history of the Araucaria forest and campos region from Serra Campos Gerais, Paraná State (South Brazil). Review of Palaeobotany and Palynology 97, 109–121. https://doi.org/10.1016/S0034-6667(96)00065-6

Behling, H., 1995. Investigations into the late Pleistocene and Holocene history of vegetation and climate in Santa Catarina (S Brazil). Vegetation History and Archaeobotany 4, 127–152. https://doi.org/10.1007/BF00203932

Behling, H., 1993. Untersuchungen zur Spatpleistozänen und Holozänen Vegetations- und Klimageschichte der Tropischen Kustenwalder und der Araukarienwälder in Santa Catarina (Sudbrasilien). Dissertationes Botanicae. J. Cramer, Stuttgart, Germany.

Behling, H., Bauermann, S.G., Neves, P.C.P. das, 2001. Holocene environmental changes in the Sao Francisco de Paula region, southern Brazil. Journal of South American Earth Sciences 14, 631–639. https://doi.org/10.1016/S0895-9811(01)00040-2

Behling, H., de Oliveira, M.A.T., 2018. Evidence of a late glacial warming event and early Holocene cooling in the southern Brazilian coastal highlands. Quaternary Research 89, 90–102. https://doi.org/10.1017/qua.2017.87

Behling, H., Dupont, L., DeForest Safford, H., Wefer, G., 2007. Late Quaternary vegetation and climate dynamics in the Serra da Bocaina, southeastern Brazil. Quaternary International 161, 22–31. https://doi.org/10.1016/j.quaint.2006.10.021

Behling, H., Jantz, N., Safford, H.D.F., 2020. Mid- and late Holocene vegetation, climate and fire dynamics in the Serra do Itatiaia, Rio de Janeiro State, southeastern Brazil. Review of Palaeobotany and Palynology 274, 104152. https://doi.org/10.1016/j.revpalbo.2019.104152

Behling, H., Pillar, V. de P., 2007. Late Quaternary vegetation, biodiversity and fire dynamics on the southern Brazilian highland and their implication for conservation and management of modern Araucaria forest and grassland ecosystems. Philosophical Transactions of the Royal Society B 362, 243–251. https://doi.org/10.1098/rstb.2006.1984

Behling, H., Pillar, V.D., Bauermann, S.G., 2005. Late Quaternary grassland (Campos), gallery forest, fire and climate dynamics, studied by pollen, charcoal and multivariate analysis of the São Francisco de Assis core in western Rio Grande do Sul (southern Brazil). Review of Palaeobotany and Palynology 133, 235–248. https://doi.org/10.1016/j.revpalbo.2004.10.004

Behling, H., Pillar, V.D., Orlóci, L., Bauermann, S.G., 2004. Late Quaternary Araucaria forest, grassland (Campos), fire and climate dynamics, studied by high-resolution pollen, charcoal and multivariate analysis of the Cambará do Sul core in southern Brazil. Palaeogeography, Palaeoclimatology, Palaeoecology 203, 277–297. https://doi.org/10.1016/S0031-0182(03)00687-4

Behling, H., Safford, H.D., 2010. Late-glacial and Holocene vegetation, climate and fire dynamics in the Serra dos Órgãos, Rio de Janeiro State, southeastern Brazil. Global Change Biology 16, 1661–1671. https://doi.org/10.1111/j.1365-2486.2009.02029.x

Behling, H., Verissimo, N., Bauermann, S., Bordignon, S., Evaldt, A., 2016. Late Holocene Vegetation History and Early Evidence of Araucaria angustifolia in Caçapava do Sul in the Lowland Region of Rio Grande do Sul State, Southern Brazil. Brazilian Archives of Biology and Technology 59. https://doi.org/10.1590/1678-4324-2016150264

Bernal, J.P., Cruz, F.W., Stríkis, N.M., Wang, X., Deininger, M., Catunda, M.C.A., Ortega-Obregón, C., Cheng, H., Edwards, R.L., Auler, A.S., 2016. High-resolution Holocene South American monsoon history recorded by a speleothem from Botuverá Cave, Brazil. Earth and Planetary Science Letters 450, 186–196. https://doi.org/10.1016/j.epsl.2016.06.008

Bertoldo, É., 2010. Registro paleoambiental em cabeceira de drenagem inscrita no remanescente de Superfície Aplainada VIII (A.R.I.E. do Buriti - SW PR). Universidade Estadual do Oeste do Paraná.

Bertoldo, É., Paisani, J.C., Oliveira, P.E. de, 2014. Registro de Floresta Ombrófila Mista nas regiões sudoeste e sul do Estado do Paraná, Brasil, durante o Pleistoceno / Holoceno. Hoehnea 1, 1–8.

Bissa, W.M., de Toledo, M.B., 2015. Late Quaternary Vegetational Changes in a Marsh Forest in Southeastern Brazil with Comments on Prehistoric Human Occupation. Radiocarbon 57, 737–753. https://doi.org/10.2458/azu_rc.57.18198

Bitencourt, A.L.V., Krauspenhar, P.M., 2006. Possible prehistoric anthropogenic effect on Araucaria angustifolia (Bert.) O. Kuntze expansion during the late Holocene. Revista Brasileira de Paleontologia 9, 109–116. https://doi.org/10.4072/rbp.2006.1.12

Blaauw, M., Christen, J.A., 2011. Flexible paleoclimate age-depth models using an autoregressive gamma process. Bayesian Analysis 6, 457–474. https://doi.org/10.1214/11-BA618

Blaauw, M., Christen, J.A., Aquino L., M.A., Esquivel Vazquez, J., Gonzalez, O.M.V., Belding, T., Theiler, J., Gough, B., Karney, C., 2020. rbacon: Age-Depth Modelling using Bayesian Statistics.

Bogoni, J.A., Batista, G.O., Graipel, M.E., Peroni, N., 2020a. Good times, bad times: Resource pulses influence mammal diversity in meridional Brazilian highlands. Science of the Total Environment 734, 139473. https://doi.org/10.1016/j.scitotenv.2020.139473

Bogoni, J.A., Graipel, M.E., Peroni, N., 2018. The ecological footprint of Acca sellowiana domestication maintains the residual vertebrate diversity in threatened highlands of Atlantic Forest. PLoS ONE 13, e0195199. https://doi.org/10.1371/journal.pone.0195199

Bogoni, J.A., Muniz-Tagliari, M., Peroni, N., Peres, C.A., 2020b. Testing the keystone plant resource role of a flagship subtropical tree species (Araucaria angustifolia) in the Brazilian Atlantic Forest. Ecological Indicators 118, 106778. https://doi.org/10.1016/j.ecolind.2020.106778

Bonomo, M., Costa Angrizani, R., Apolinaire, E., Noelli, F.S., 2015. A model for the Guaraní expansion in the La Plata Basin and littoral zone of southern Brazil. Quaternary International 356, 54–73. https://doi.org/10.1016/j.quaint.2014.10.050

Brown, J.L., Paz, A., Reginato, M., Renata, C.A., Assis, C., Lyra, M., Caddah, M.K., Aguirre-Santoro, J., d’Horta, F., Raposo do Amaral, F., Goldenberg, R., Lucas Silva-Brandão, K., Freitas, A.V.L., Rodrigues, M.T., Michelangeli, F.A., Miyaki, C.Y., Carnaval, A.C., 2020. Seeing the forest through many trees: Multi-taxon patterns of phylogenetic diversity in the Atlantic Forest hotspot. Diversity and Distributions 26, 1160–1176. https://doi.org/10.1111/ddi.13116

Clement, C.R., Casas, A., Parra-Rondinel, F.A., Levis, C., Peroni, N., Hanazaki, N., Cortés-Zárraga, L., Rangel-Landa, S., Alves, R.P., Ferreira, M.J., Cassino, M.F., Coelho, S.D., Cruz-Soriano, A., Pancorbo-Olivera, M., Blancas, J., Martínez-Ballesté, A., Lemes, G., Lotero-Velásquez, E., Bertin, V.M., Mazzochini, G.G., 2021. Disentangling Domestication from Food Production Systems in the Neotropics. Quaternary 4. https://doi.org/10.3390/quat4010004

Corteletti, R., Dickau, R., DeBlasis, P., Iriarte, J., 2015. Revisiting the economy and mobility of southern proto-Jê (Taquara-Itararé) groups in the southern Brazilian highlands: Starch grain and phytoliths analyses from the Bonin site, Urubici, Brazil. Journal of Archaeological Science 58, 46–61. https://doi.org/10.1016/j.jas.2015.03.017

Corteletti, R., Labrador, B., DeBlasis, P., 2023. An Archaeology of Social Jê Landscapes at Urubici, Santa Catarina, in: Historical Ecology and Landscape Archaeology in Lowland South America, Interdisciplinary Contributions to Archaeology. Springer, pp. 151–179. https://doi.org/10.1007/978-3-031-32284-6_7

Cruz, T.M.S., 2014. Etnoecologia de paisagens na Terra Indígena Ibirama Laklãnõ, Santa Catarina, Brasil. Universidade Federal de Santa Catarina.

de Azevedo, L.W., Scheel-Ybert, R., 2020. Contributions to Proto-Jê Archaeology in the Southern Brazilian Highlands: Wood, Fire, and Landscape. Latin American Antiquity 31, 325–341. https://doi.org/10.1017/laq.2020.1

de Lima, R.A.F., Mori, D.P., Pitta, G., Melito, M.O., Bello, C., Magnago, L.F., Zwiener, V.P., Saraiva, D.D., Marques, M.C.M., de Oliveira, A.A., Prado, P.I., 2015. How much do we know about the endangered Atlantic Forest? Reviewing nearly 70 years of information on tree community surveys. Biodiversity and Conservation 24, 2135–2148. https://doi.org/10.1007/s10531-015-0953-1

de Oliveira Portes, M.C.G., Behling, H., Montade, V., Safford, H.D., 2020. Holocene vegetation, climate and fire dynamics in the Serra dos Órgãos, Rio de Janeiro State, southeastern Brazil. Acta Palaeobotanica 438–453. https://doi.org/10.35535/acpa-2020-0019

de Oliveira Portes, M.C.G., Safford, H., Behling, H., 2018. Humans and climate as designers of the landscape in Serra da Bocaina National Park, southeastern Brazil, over the last seven centuries. Anthropocene 24, 61–71. https://doi.org/10.1016/j.ancene.2018.11.004

de Souza, J.G., 2018. Rethinking households, communities and status in the southern Brazilian highlands. Journal of Anthropological Archaeology 52, 44–58. https://doi.org/10.1016/j.jaa.2018.08.006

de Souza, J.G., Corteletti, R., Robinson, M., Iriarte, J., 2016a. The genesis of monuments: Resisting outsiders in the contested landscapes of southern Brazil. Journal of Anthropological Archaeology 41, 196–212. https://doi.org/10.1016/j.jaa.2016.01.003

de Souza, J.G., Robinson, M., Corteletti, R., Cárdenas, M.L., Wolf, S., Iriarte, J., Mayle, F., DeBlasis, P., 2016b. Understanding the chronology and occupation dynamics of oversized pit houses in the southern Brazilian highlands. PLoS ONE 11, 1–24. https://doi.org/10.1371/journal.pone.0158127

dos Reis, M.S., Ladio, A., Peroni, N., 2014. Landscapes with Araucaria in South America: Evidence for a cultural dimension. Ecology and Society 19, 43. https://doi.org/10.5751/ES-06163-190243

dos Reis, M.S., Montagna, T., Mattos, A.G., Filippon, S., Ladio, A.H., Marques, A. da C., Zechini, A.A., Peroni, N., Mantovani, A., 2018. Domesticated Landscapes in Araucaria Forests, Southern Brazil: A Multispecies Local Conservation-by-Use System. Frontiers in Ecology and Evolution 6, 11. https://doi.org/10.3389/fevo.2018.00011

dos Santos, K.L., Peroni, N., Guries, R.P., Nodari, R.O., 2009. Traditional Knowledge and Management of Feijoa (Acca sellowiana) in Southern Brazil. Economic Botany 63, 204–214.

Dümig, A., Schad, P., Rumpel, C., Dignac, M.F., Kögel-Knabner, I., 2008. Araucaria forest expansion on grassland in the southern Brazilian highlands as revealed by 14C and δ13C studies. Geoderma 145, 143–157. https://doi.org/10.1016/j.geoderma.2007.06.005

Fernandes, R.C., Piovezana, L., 2015. The Kaingang perspectives on land and environmental rights in the south of Brazil. Ambiente & Sociedade 18, 111–128. https://doi.org/10.1590/1809-4422ASOCEx07V1822015en

Fernandes, R.S., 2009. Reconstrução Paleoambiental da Lagoa Fazenda Durante o Neopleistoceno e Holoceno na Região de Jussara, Estado do Paraná, com Ênfase em Estudos Palinológicos. Universidade Gaurulhos.

Flantua, S.G.A., Blaauw, M., Hooghiemstra, H., 2016. Geochronological database and classification system for age uncertainties in Neotropical pollen records. Climate of the Past 12, 387–414. https://doi.org/10.5194/cp-12-387-2016

Fletcher, M.-S., Hamilton, R., Dressler, W., Palmer, L., 2021. Indigenous knowledge and the shackles of wilderness. Proceedings of the National Academy of Sciences 118, e2022218118. https://doi.org/10.1073/pnas.2022218118

Fourcade, Y., Besnard, A.G., Secondi, J., 2017. Paintings predict the distribution of species, or the challenge of selecting environmental predictors and evaluation statistics. Global Ecology and Biogeography. https://doi.org/10.1111/geb.12684

Garcia, M.J., De Oliveira, P.E., de Siqueira, E., Fernandes, R.S., 2004. A Holocene vegetational and climatic record from the Atlantic rainforest belt of coastal State of São Paulo, SE Brazil. Review of Palaeobotany and Palynology 131, 181–199. https://doi.org/10.1016/j.revpalbo.2004.03.007

Gessert, S., Iriarte, J., Ríos, R.C., Behling, H., 2011. Late Holocene vegetation and environmental dynamics of the Araucaria forest region in Misiones Province, NE Argentina. Review of Palaeobotany and Palynology 166, 29–37. https://doi.org/10.1016/j.revpalbo.2011.04.006

Gomes, T.C.C., 2018. Paisagens culturais e biodiversidade: Mudanças socioecológicas e estratégias locais para conservação na Terra Indígena Laklãnõ, Santa Catarina, Brasil 82.

Grantham, H.S., Duncan, A., Evans, T.D., Jones, K.R., Beyer, H.L., Schuster, R., Walston, J., Ray, J.C., Robinson, J.G., Callow, M., Clements, T., Costa, H.M., DeGemmis, A., Elsen, P.R., Ervin, J., Franco, P., Goldman, E., Goetz, S., Hansen, A., Hofsvang, E., Jantz, P., Jupiter, S., Kang, A., Langhammer, P., Laurance, W.F., Lieberman, S., Linkie, M., Malhi, Y., Maxwell, S., Mendez, M., Mittermeier, R., Murray, N.J., Possingham, H., Radachowsky, J., Saatchi, S., Samper, C., Silverman, J., Shapiro, A., Strassburg, B., Stevens, T., Stokes, E., Taylor, R., Tear, T., Tizard, R., Venter, O., Visconti, P., Wang, S., Watson, J.E.M., 2020. Anthropogenic modification of forests means only 40% of remaining forests have high ecosystem integrity. Nature Communications 11, 1–10. https://doi.org/10.1038/s41467-020-19493-3

Gu, F., Chiessi, C.M., Zonneveld, K.A.F., Behling, H., 2018. Late Quaternary environmental dynamics inferred from marine sediment core GeoB6211-2 off southern Brazil. Palaeogeography, Palaeoclimatology, Palaeoecology 496, 48–61. https://doi.org/10.1016/j.palaeo.2018.01.015

Gu, F., Zonneveld, K.A.F., Chiessi, C.M., Arz, H.W., Pätzold, J., Behling, H., 2017. Long-term vegetation, climate and ocean dynamics inferred from a 73,500 years old marine sediment core (GeoB2107-3) off southern Brazil. Quaternary Science Reviews 172, 55–71. https://doi.org/10.1016/j.quascirev.2017.06.028

Heaton, T.J., Köhler, P., Butzin, M., Bard, E., Reimer, R.W., Austin, W.E.N., Bronk Ramsey, C., Grootes, P.M., Hughen, K.A., Kromer, B., Reimer, P.J., Adkins, J., Burke, A., Cook, M.S., Olsen, J., Skinner, L.C., 2020. Marine20—The Marine Radiocarbon Age Calibration Curve (0–55,000 cal BP). Radiocarbon 62, 779–820. https://doi.org/10.1017/RDC.2020.68

Henry, J., 1964. Jungle people: A Kaingáng tribe of the highlands of Brazil. Random House, New York, NY.

Hogg, A.G., Heaton, T.J., Hua, Q., Palmer, J.G., Turney, C.S., Southon, J., Bayliss, A., Blackwell, P.G., Boswijk, G., Bronk Ramsey, C., Pearson, C., Petchey, F., Reimer, P., Reimer, R., Wacker, L., 2020. SHCal20 Southern Hemisphere Calibration, 0–55,000 Years cal BP. Radiocarbon 62, 759–778. https://doi.org/10.1017/RDC.2020.59

IBGE - Instituto Brasileiro de Geografia e Estatística, 2019. Macrocaracterização dos recursos naturais do Brasil: províncias estruturais, compartimentos de relevo, tipos de solos, regiões fitoecológicas e outras areas.

IBGE - Instituto Brasileiro de Geografia e Estatística, 2018. Mapeamento de Recurso Naturais do Brasil: Escala 1:250.000.

Iriarte, J., Moehlecke Copé, S., Fradley, M., Lockhart, J.J., Gillam, J.C., 2013. Sacred landscapes of the southern Brazilian highlands: Understanding southern proto-Je mound and enclosure complexes. Journal of Anthropological Archaeology 32, 74–96. https://doi.org/10.1016/j.jaa.2012.10.003

Iriarte, J., Smith, R.J., de Souza, J.G., Mayle, F.E., Whitney, B.S., Cárdenas, M.L., Singarayer, J., Carson, J.F., Roy, S., Valdes, P., 2017. Out of Amazonia: Late-Holocene climate change and the Tupi–Guarani trans-continental expansion. The Holocene 27, 967–975. https://doi.org/10.1177/0959683616678461

Jeske-Pieruschka, V., Behling, H., 2012. Palaeoenvironmental history of the São Francisco de Paula region in southern Brazil during the late Quaternary inferred from the Rincão das Cabritas core. The Holocene 22, 1251–1262. https://doi.org/10.1177/0959683611414930

Jeske-Pieruschka, V., Fidelis, A., Bergamin, R.S., Vélez, E., Behling, H., 2010. Araucaria forest dynamics in relation to fire frequency in southern Brazil based on fossil and modern pollen data. Review of Palaeobotany and Palynology 160, 53–65. https://doi.org/10.1016/j.revpalbo.2010.01.005

Jeske-Pieruschka, V., Pillar, V.D., de Oliveira, M.A.T., Behling, H., 2013. New insights into vegetation, climate and fire history of southern Brazil revealed by a 40,000 year environmental record from the State Park Serra do Tabuleiro. Vegetation History and Archaeobotany 22, 299–314. https://doi.org/10.1007/s00334-012-0382-y

Karger, D.N., 2021. Downscaled transient temperature and precipitation data since the last glacial maximum. CHELSA-TraCE21k v1.0: Technical specification.

Karger, D.N., Conrad, O., Böhner, J., Kawohl, T., Kreft, H., Soria-Auza, R.W., Zimmermann, N.E., Linder, H.P., Kessler, M., 2017. Climatologies at high resolution for the earth’s land surface areas. Scientific Data 4, 170122. https://doi.org/10.1038/sdata.2017.122

Karger, D.N., Nobis, M., Normand, S., Graham, C., Zimmermann, N., 2021. CHELSA-TraCE21k v1.0. Downscaled transient temperature and precipitation data since the last glacial maximum. Climate of the Past Discussions 1–27. https://doi.org/10.5194/cp-2021-30

Karger, D.N., Zimmermann, N.E., 2019. Climatologies at High resolution for the Earth Land Surface Areas CHELSA V1.2: Technical specification v1.1. https://doi.org/10.5061/dryad.kd1d4

Kern, A., 1998. Antecedentes Indígenas : Problemáticas teórico-metodológicas das sínteses sobre a pré-história regional. Revista do Museu de Arqueologia e Etnologia 15–24.

Klein, R.M., 1975. Southern Brazilian phytogeographic features and the probable influence of upper Quaternary climatic changes in the floristic distribution. Boletim Paranaense de Geociências 33, 67–88.

Ladchuk, D.P.P.T., Parolin, M., Bauermann, S.G., 2016. Recuperação de palinomorfos e dados isotópicos (δ13C e δ15N) em sedimentos turfosos e seu significado paleoambiental para a região de Campo Mourão-PR. Revista Brasileira de Geografia Física 09, 1183–1196.

Lauterjung, M.B., Bernardi, A.P., Montagna, T., Candido-Ribeiro, R., da Costa, N.C.F., Mantovani, A., dos Reis, M.S., 2018. Phylogeography of Brazilian pine (Araucaria angustifolia): integrative evidence for pre-Columbian anthropogenic dispersal. Tree Genetics & Genomes 14, 36. https://doi.org/10.1007/s11295-018-1250-4

Ledru, M.-P., Mourguiart, P., Riccomini, C., 2009. Related changes in biodiversity, insolation and climate in the Atlantic rainforest since the last interglacial. Palaeogeography, Palaeoclimatology, Palaeoecology 271, 140–152. https://doi.org/10.1016/j.palaeo.2008.10.008

Leonhardt, A., Lorscheitter, M.L., 2010. The last 25,000 years in the Eastern Plateau of Southern Brazil according to Alpes de São Francisco record. Journal of South American Earth Sciences 29, 454–463. https://doi.org/10.1016/j.jsames.2009.09.003

Levis, C., Flores, B.M., Moreira, P.A., Luize, B.G., Alves, R.P., Franco-Moraes, J., Lins, J., Konings, E., Peña-Claros, M., Bongers, F., Costa, F.R.C., Clement, C.R., 2018. How People Domesticated Amazonian Forests. Frontiers in Ecology and Evolution 5, 1–21. https://doi.org/10.3389/fevo.2017.00171

Luz, L.D., Parolin, M., Pessenda, L.C.R., Rasbold, G.G., Lo, E., 2019. Multiproxy analysis (Phytoliths, stable isotopes, and C/N) as indicators of paleoenvironmental changes in a Cerrado site, southern Brazil. Revista Brasileira de Paleontologia 22, 15–29. https://doi.org/10.4072/rbp.2019.1.02

Machado, J.S., Tschucambang, C., Fonseca, J.R., 2020. Stones, Clay and People Among the Laklãnõ Xokleng Indigenous People in Southern Brazil. Archaeologies 1–32. https://doi.org/10.1007/s11759-020-09405-8

McCune, B., 2007. Improved estimates of incident radiation and heat load using non-parametric regression against topographic variables. Journal of Vegetation Science 18, 751–754. https://doi.org/10.1658/1100-9233(2007)18%5B751:IEOIRA%5D2.0.CO;2

McCune, B., Keon, D., 2002. Equations for Potential Annual Direct Incident Radiation and Heat Load. Journal of Vegetation Science 13, 603–606.

Mello, A.J.M., Peroni, N., 2015. Cultural landscapes of the Araucaria Forests in the northern plateau of Santa Catarina, Brazil. Journal of Ethnobiology and Ethnomedicine 11, 51. https://doi.org/10.1186/s13002-015-0039-x

Moura, D.A.S. de, 2021. Etnopaisagem Jê e reterritorialização do Brasil Meridional (1768-1773). Revista Brasileira de História 42, 187–212. https://doi.org/10.1590/1806-93472021v42n87-10

Naimi, B., Hamm, N.A.S., Groen, T.A., Skidmore, A.K., Toxopeus, A.G., 2014. Where is positional uncertainty a problem for species distribution modelling? Ecography 37, 191–203. https://doi.org/10.1111/j.1600-0587.2013.00205.x

NASA, METI, AIST, Spacesystems, J., Team, U.S. /Japan A.S., 2019. ASTER Global Digital Elevation Model V003. https://doi.org/10.5067/ASTER/ASTGTM.003

Noelli, F.S., 2000. A ocupação humana na Região Sul do Brasil: Arqueologia, debates e perspectivas 1872-2000. Revista da Universidade da São Paulo 44, 218–269.

Oliveira-Filho, A.T., Budke, J.C., Jarenkow, J.A., Eisenlohr, P.V., Neves, D.R.M., 2014. Delving into the variations in tree species composition and richness across South American subtropical Atlantic and Pampean forests. Journal of Plant Ecology 8, 242–260. https://doi.org/10.1093/jpe/rtt058

Olson, D.M., Dinerstein, E., Wikramanayake, E.D., Burgess, N.D., Powell, G.V.N., Underwood, E.C., D’Amico, J.A., Itoua, I., Strand, H.E., Morrison, J.C., Loucks, C.J., Allnutt, T.F., Ricketts, T.H., Kura, Y., Lamoreux, J.F., Wettengel, W.W., Hedao, P., Kassem, K.R., 2001. Terrestrial ecoregions of the world: A new map of life on Earth. BioScience 51, 933–938. https://doi.org/10.1641/0006-3568(2001)051[0933:TEOTWA]2.0.CO;2

Pereira Cruz, A., Giehl, E.L.H., Levis, C., Machado, J.S., Bueno, L., Peroni, N., 2020. Pre-colonial Amerindian legacies in forest composition of southern Brazil. PLOS ONE 15, e0235819. https://doi.org/10.1371/journal.pone.0235819

Pessenda, L.C.R., De Oliveira, P.E., Mofatto, M., de Medeiros, V.B., Francischetti Garcia, R.J., Aravena, R., Bendassoli, J.A., Zuniga Leite, A., Saad, A.R., Lincoln Etchebehere, M., 2009. The evolution of a tropical rainforest/grassland mosaic in southeastern Brazil since 28,000 14 C yr BP based on carbon isotopes and pollen records. Quaternary Research 71, 437–452. https://doi.org/10.1016/j.yqres.2009.01.008

Rasbold, G.G., Parolin, M., Caxambu, M.G., 2016. Reconstrução paleoambiental de um depósito sedimentar por análises multiproxy, Turvo, estado do Paraná, Brasil. Revista Brasileira de Paleontologia 19, 315–324. https://doi.org/10.4072/rbp.2016.2.13

Réus Gonçalves Da Rosa, R., 2005. O Território Xamânico Kaingang Vinculado às Bacias Hidrográficas e à Floresta de Araucária. Cadernos do LEPAARQ (UFPEL) 2, 99–116. https://doi.org/10.15210/lepaarq.v2i4.888

Ribeiro, M.B.N., Jerozolimski, A., De Robert, P., Salles, N.V., Kayapó, B., Pimentel, T.P., Magnusson, W.E., 2014. Anthropogenic landscape in southeastern Amazonia: Contemporary impacts of low-intensity harvesting and dispersal of Brazil nuts by the Kayapó indigenous people. PLoS ONE 9, e102187. https://doi.org/10.1371/journal.pone.0102187

Richer, S., Gearey, B., 2017. The Medicine Tree: Unsettling palaeoecological perceptions of past environments and human activity. Journal of Social Archaeology 17, 239–262. https://doi.org/10.1177/1469605317731013

Riris, P., Arroyo-Kalin, M., 2019. Widespread population decline in South America correlates with mid-Holocene climate change. Scientific Reports 9, 6850. https://doi.org/10.1038/s41598-019-43086-w

Robinson, M., de Souza, J.G., Maezumi, S.Y., Cárdenas, M., Pessenda, L., Prufer, K., Corteletti, R., Scunderlick, D., Mayle, F.E., De Blasis, P., Iriarte, J., 2018. Uncoupling human and climate drivers of late Holocene vegetation change in southern Brazil. Scientific Reports 8, 7800. https://doi.org/10.1038/s41598-018-24429-5

Robinson, M., Iriarte, J., de Souza, J.G., Corteletti, R., Ulguim, P., Fradley, M., Cárdenas, M., De Blasis, P., Mayle, F., Scunderlick, D., 2017. Moieties and Mortuary Mounds: Dualism at a Mound and Enclosure Complex in the Southern Brazilian Highlands. Latin American Antiquity 28, 232–251. https://doi.org/10.1017/laq.2017.11

Roderjan, C.V., Galvão, F., Kuniyoshi, Y.S., Hatschbach, G.G., 2002. As unidades fitogeográficas do estado do Paraná, Brasil. Ciência & Ambiente 24, 75–92.

Saia, S.E.M.G., 2006. Reconstrução paleoambiental (vegetação e clima) no quartenário tardio com base em estudo multi/interdisciplinar no vale do Ribeira (sul do estado de São Paulo). Universidade de São Paulo.

Scheel-Ybert, R., Boyadjian, C., 2020. Gardens on the coast: Considerations on food production by Brazilian shellmound builders. Journal of Anthropological Archaeology 60, 101211. https://doi.org/10.1016/j.jaa.2020.101211

Scheer, M.B., Pereira, N.V., Behling, H., Curcio, G.R., Roderjan, C.V., 2014. Nine thousand years of upper montane soil/vegetation dynamics from the summit of Caratuva Peak, Southern Brazil. Journal of South American Earth Sciences 56, 365–375. https://doi.org/10.1016/j.jsames.2014.09.019

Scherer, C., Lorscheitter, M.L., 2014. Vegetation dynamics in the southern Brazilian highlands during the last millennia and the role of bogs in Araucaria forest formation. Quaternary International 325, 3–12. https://doi.org/10.1016/j.quaint.2014.01.010

Schindelin, J., Arganda-Carreras, I., Frise, E., Kaynig, V., Longair, M., Pietzsch, T., Preibisch, S., Rueden, C., Saalfeld, S., Schmid, B., Tinevez, J.Y., White, D.J., Hartenstein, V., Eliceiri, K., Tomancak, P., Cardona, A., 2012. Fiji: An open-source platform for biological-image analysis. Nature Methods 9, 676–682. https://doi.org/10.1038/nmeth.2019

Schorn, L.A., de Gasper, A.L., Meyer, L., Vibrans, A.C., 2012. Síntese da estrutura dos remanescentes florestais em Santa Catarina, in: Vibrans, A.C., Sevegnani, L., de Gasper, A.L., Lingner, D.V. (Eds.), Inventário Florístico Florestal de Santa Catarina Volume I - Diversidade e Conservação Dos Remanescentes Florestais. Edifurb, Blumenau, pp. 125–140.

Shepard, G., Ramirez, H., 2011. “Made in Brazil”: Human Dispersal of the Brazil Nut (Bertholletia excelsa, Lecythidaceae) in Ancient Amazonia. Economic Botany 65, 44–65. https://doi.org/10.1007/s12231-011-9151-6

Silva, D.W. da, 2018. Caracterização paleoclimática do quaternário tardio em áreas planálticas do Estado do Paraná 126.

Silva, L.C.R., Anand, M., 2011. Mechanisms of Araucaria (Atlantic) Forest Expansion into Southern Brazilian Grasslands. Ecosystems 14, 1354–1371. https://doi.org/10.1007/s10021-011-9486-y

Smith, R.J., Mayle, F.E., 2017. Impact of mid- to late Holocene precipitation changes on vegetation across lowland tropical South America: a paleo-data synthesis. Quaternary Research 89, 1–22. https://doi.org/10.1017/qua.2017.89

Souza, A.F., 2021. A review of the structure and dynamics of araucaria mixed forests in southern Brazil and northern Argentina. New Zealand Journal of Botany 59, 2–54. https://doi.org/10.1080/0028825X.2020.1810712

Souza, C.M., Z. Shimbo, J., Rosa, M.R., Parente, L.L., A. Alencar, A., Rudorff, B.F.T., Hasenack, H., Matsumoto, M., G. Ferreira, L., Souza-Filho, P.W.M., de Oliveira, S.W., Rocha, W.F., Fonseca, A. V., Marques, C.B., Diniz, C.G., Costa, D., Monteiro, D., Rosa, E.R., Vélez-Martin, E., Weber, E.J., Lenti, F.E.B., Paternost, F.F., Pareyn, F.G.C., Siqueira, J. V., Viera, J.L., Neto, L.C.F., Saraiva, M.M., Sales, M.H., Salgado, M.P.G., Vasconcelos, R., Galano, S., Mesquita, V. V., Azevedo, T., 2020. Reconstructing Three Decades of Land Use and Land Cover Changes in Brazilian Biomes with Landsat Archive and Earth Engine. Remote Sensing 12, 2735. https://doi.org/10.3390/rs12172735

Spalding, B.B. da C., Lorscheitter, M.L., 2015. Dry and humid phases in the highlands of southern Brazil during the last 34,000 years, and their influence on the paleoenvironments of the region. Quaternary International 377, 102–111. https://doi.org/10.1016/j.quaint.2014.11.057

Stefenon, V.M., Klabunde, G., Lemos, R.P.M., Rogalski, M., Nodari, R.O., 2019. Phylogeography of plastid DNA sequences suggests post-glacial southward demographic expansion and the existence of several glacial refugia for Araucaria angustifolia. Scientific Reports 9, 2752. https://doi.org/10.1038/s41598-019-39308-w

Sühs, R.B., Rosa, F.S., Silveira, J., Peroni, N., Giehl, E.L.H., 2021. The influence of fire and cattle grazing on Araucaria population structure in forest-grasslands mosaics. Flora 281, 151853. https://doi.org/10.1016/j.flora.2021.151853

Tella, J.L., Dénes, F.V., Zulian, V., Prestes, N.P., Martínez, J., Blanco, G., Hiraldo, F., 2016. Endangered plant-parrot mutualisms: seed tolerance to predation makes parrots pervasive dispersers of the Parana pine. Sci Rep 6, 31709. https://doi.org/10.1038/srep31709

Thomas, E., Alcázar Caicedo, C., Mcmichael, C.H., Corvera, R., Loo, J., 2015. Uncovering spatial patterns in the natural and human history of Brazil nut (Bertholletia excelsa) across the Amazon Basin. Journal of Biogeography 42, 1367–1382. https://doi.org/10.1111/jbi.12540

Universidade Federal de Santa Maria, 2001. Inventário Florestal Contínuo do Rio Grande do Sul - http://coralx.ufsm.br/ifcrs/frame.htm.

Vasconcellos, M.M., Varela, S., Reginato, M., Gehara, M., Carnaval, A.C., Michelangeli, F.A., 2024. Evaluating the impact of historical climate and early human groups in the Araucaria Forest of eastern South America. Ecography 2024, e06756. https://doi.org/10.1111/ecog.06756

Vibrans, A.C., De Gasper, A.L., Moser, P., Oliveira, L.Z., Lingner, D.V., Sevegnani, L., 2020. Insights from a large-scale inventory in the southern Brazilian Atlantic Forest. Scientia Agricola 77. https://doi.org/10.1590/1678-992x-2018-0036

Vibrans, A.C., Sevegnani, L., Lingner, D.V., de Gasper, A.L., Sabbagh, S., 2010. Inventário florístico florestal de Santa Catarina (IFFSC): aspectos metodológicos e operacionais. Pesquisa Florestal Brasileira 30, 291–302. https://doi.org/10.4336/2010.pfb.64.291

Wesolowski, V., Ferraz Mendonça de Souza, S.M., Reinhard, K.J., Ceccantini, G., 2010. Evaluating microfossil content of dental calculus from Brazilian sambaquis. Journal of Archaeological Science 37, 1326–1338. https://doi.org/10.1016/j.jas.2009.12.037

Wilson, O.J., Marchant, R.A., 2025. Using 3D pollen models and participatory palaeoecology to connect people with intangible palaeoenvironmental records. Quaternary Newsletter 165, 41–47.

Wilson, O.J., Mayle, F.E., Walters, R.J., Lingner, D. V., Vibrans, A.C., 2021. Floristic change in Brazil’s southern Atlantic Forest biodiversity hotspot: From the Last Glacial Maximum to the late 21st Century. Quaternary Science Reviews 264, 107005. https://doi.org/10.1016/j.quascirev.2021.107005

Wilson, O.J., Walters, R.J., Mayle, F.E., Lingner, D.V., Vibrans, A.C., 2019. Cold spot microrefugia hold the key to survival for Brazil’s Critically Endangered Araucaria tree. Global Change Biology 25, 4339–4351. https://doi.org/10.1111/gcb.14755

Yannic, G., Hagen, O., Leugger, F., Karger, D.N., Pellissier, L., 2020. Harnessing paleo‐environmental modeling and genetic data to predict intraspecific genetic structure. Evolutionary Applications 13, 1526–1542. https://doi.org/10.1111/eva.12986

Zeder, M.A., 2016. Domestication as a model system for niche construction theory. Evolutionary Ecology 2015 30:2 30, 325–348. https://doi.org/10.1007/S10682-015-9801-8

Zizka, A., Silvestro, D., Andermann, T., Azevedo, J., Ritter, C.D., Edler, D., Farooq, H., Herdean, A., Ariza, M., Scharn, R., Svantesson, S., Wengström, N., Zizka, V., Antonelli, A., 2019. CoordinateCleaner: Standardized cleaning of occurrence records from biological collection databases. Methods in Ecology and Evolution 10, 744–751. https://doi.org/10.1111/2041-210X.13152

Zurita-Benavides, M.G., Jarrín–V, P., Rios, M., 2016. Oral History Reveals Landscape Ecology in Ecuadorian Amazonia: Time Categories and Ethnobotany among Waorani People. Economic Botany 70, 1–14. https://doi.org/10.1007/s12231-015-9330-y

Zwiener, V.P., Padial, A.A., Marques, M.C.M., Faleiro, F. V., Loyola, R., Peterson, A.T., 2017. Planning for conservation and restoration under climate and land use change in the Brazilian Atlantic Forest. Diversity and Distributions 23, 955–966. https://doi.org/10.1111/ddi.12588
