# Supplementary figures and images for: How climate, Indigenous people, and fire shaped Brazil’s Araucaria Forests through the Late Holocene
Source: Sci Rep. 2026 Mar 28;16:10810. doi: 10.1038/s41598-026-41607-y (PMC13039178; doi:10.1038/s41598-026-41607-y)

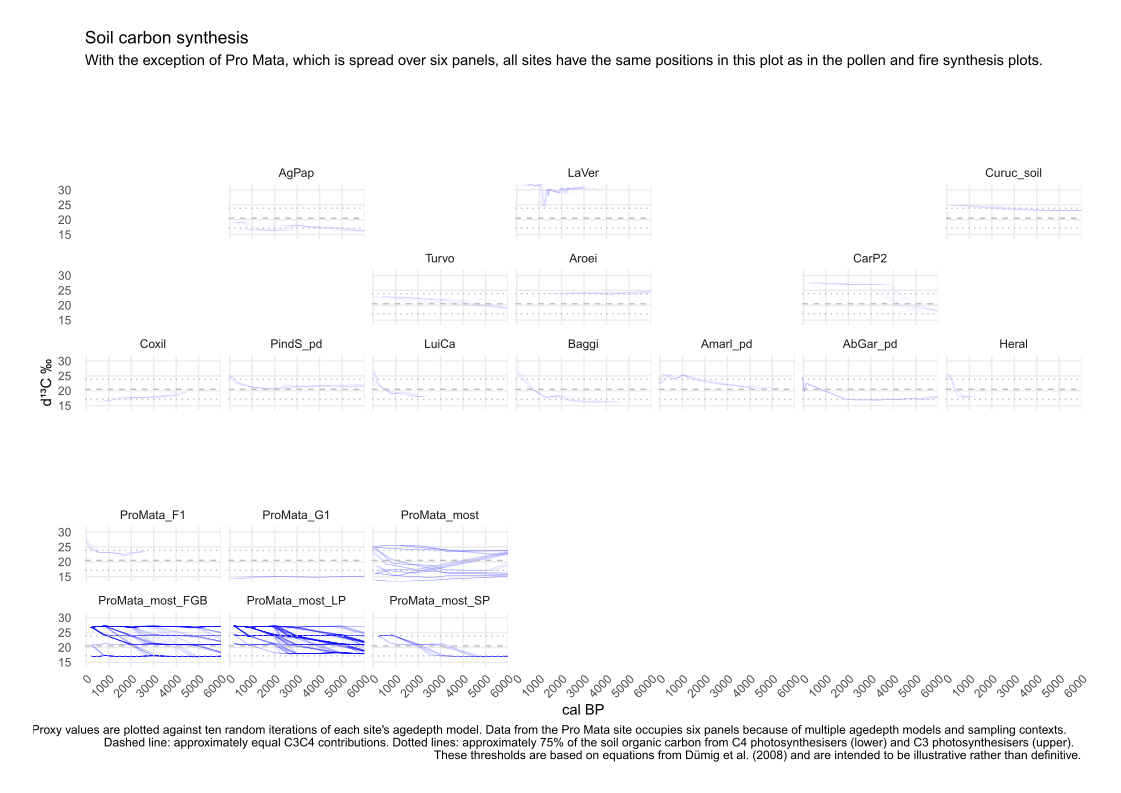

Supplement: Supplementary file 2 — Supplementary Material 2 [file 41598_2026_41607_MOESM2_ESM.zip › All proxies grids - d13C edited-1.png]

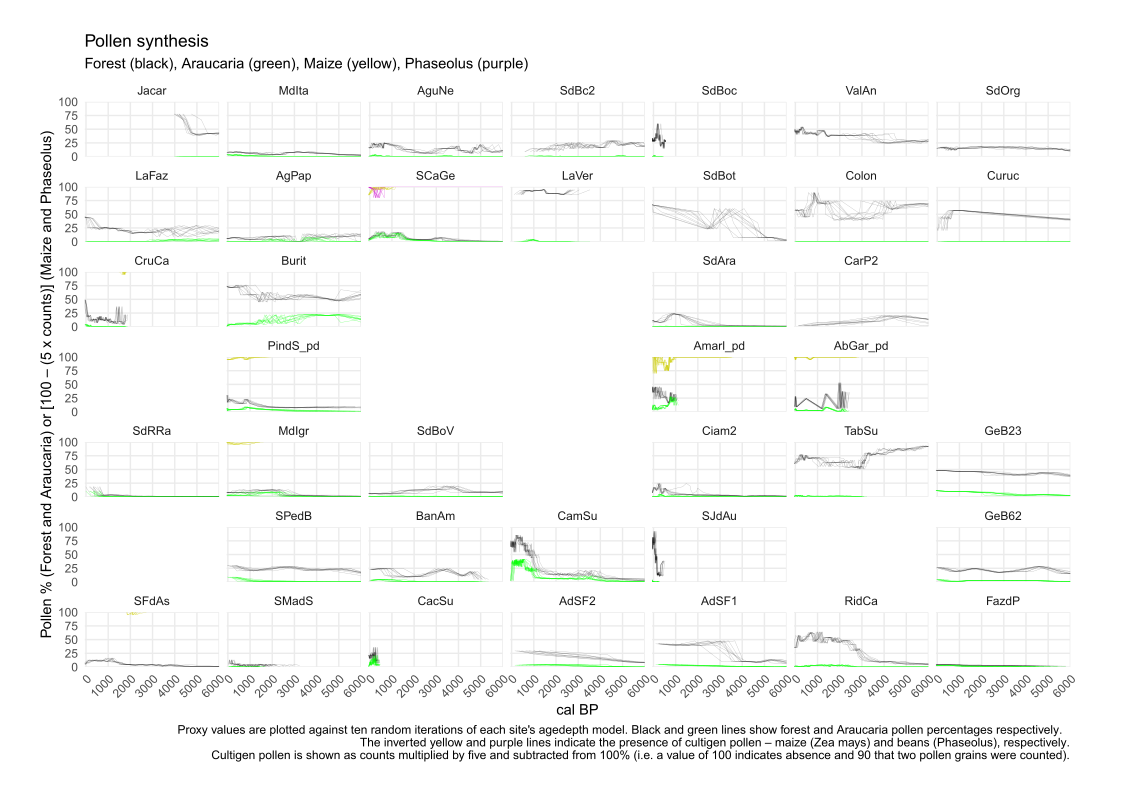

Supplement: Supplementary file 2 — Supplementary Material 2 [file 41598_2026_41607_MOESM2_ESM.zip › All proxies grids - pollen edited-1.png]

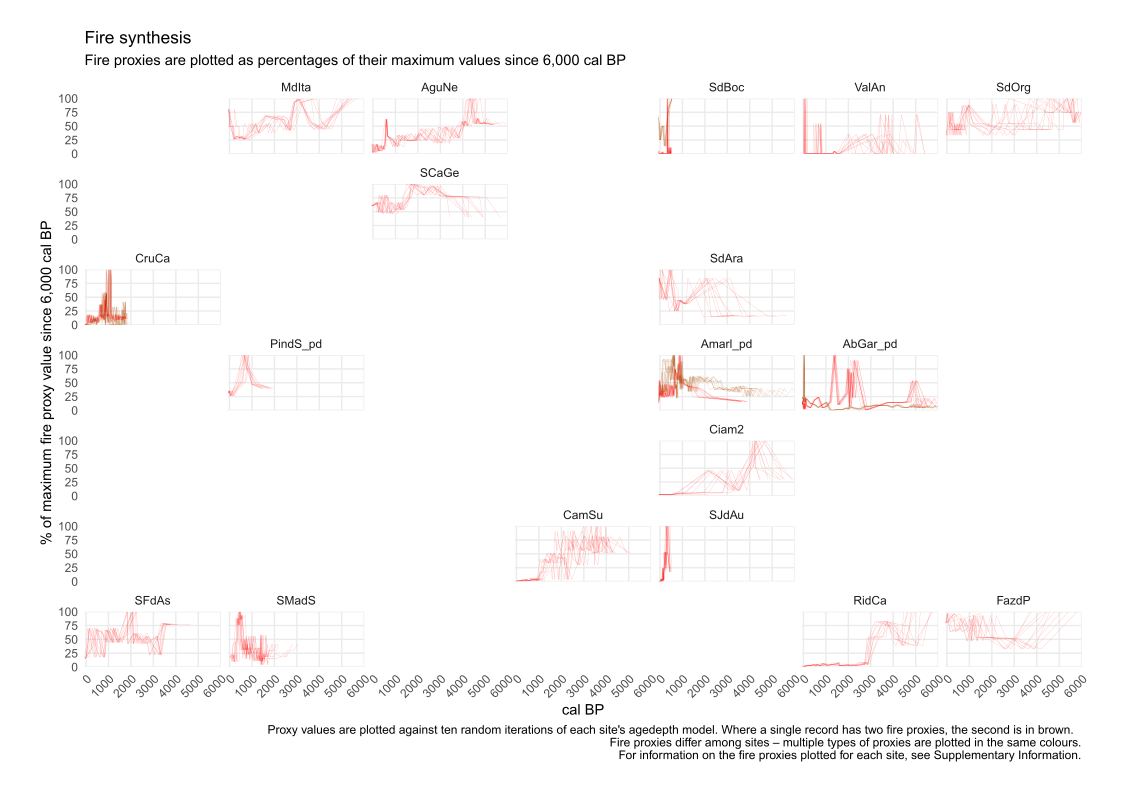

Supplement: Supplementary file 2 — Supplementary Material 2 [file 41598_2026_41607_MOESM2_ESM.zip › All proxies grids - fire edited-1.png]
